# Novel Alkyl(aryl)-Substituted 2,2-Difluoro-6-(trichloromethyl)-2*H*-1,3,2-oxazaborinin-3-ium-2-uides: Synthesis, Antimicrobial Activity, and CT-DNA Binding Evaluations

**DOI:** 10.3389/fphar.2020.01328

**Published:** 2020-09-02

**Authors:** Wilian C. Rosa, Inaiá O. Rocha, Melissa B. Rodrigues, Helena S. Coelho, Laura B. Denardi, Pauline C. Ledur, Nilo Zanatta, Thiago V. Acunha, Bernardo A. Iglesias, Helio G. Bonacorso

**Affiliations:** ^1^Núcleo de Química de Heterociclos (NUQUIMHE), Departamento de Química, Universidade Federal de Santa Maria, Santa Maria, Brazil; ^2^Laboratório de Pesquisas Micológicas (LAPEMI), Departamento de Microbiologia e Parasitologia, Universidade Federal de Santa Maria, Santa Maria, Brazil; ^3^Instituto Federal de Educação, Ciência e Tecnologia Farroupilha, Santa Maria, Brazil; ^4^Laboratório de Bioinorgânica e Materiais Porfirínicos, Departamento de Química, Universidade Federal de Santa Maria, Santa Maria, Brazil

**Keywords:** β-enaminoketones, difluoro-organoboron complexes, antimicrobial agents, DNA-binding assays, photophysical properties

## Abstract

The synthesis, antimicrobial activity evaluations, biomolecule-binding properties (DNA), and absorption and emission properties of a new series of (*Z*)-1,1,1-trichloro-4-alkyl(aryl)amino-4-arylbut-3-en-2-ones **(4, 5)** and 2,2-difluoro-3-alkyl(aryl)amino-4-aryl-6-(trichloromethyl)-2*H*-1,3,2-oxazaborinin-3-ium-2-uides **(6, 7)** in which 3(4)-alkyl(aryl) = H, Me, *iso*-propyl, *n*-butyl, C_6_H_5_, 4-CH_3_C_6_H_4_, 4-CH_3_OC_6_H_4_, 4-NO_2_C_6_H_4_, 4-FC_6_H_4_, 4-BrC_6_H_4_, 2-naphthyl, is reported. A series of β-enaminoketones **(4, 5)** is synthesized from the O,N-exchange reaction of some amines **(3)** with (*Z*)-1,1,1-trichloro-4-methoxy-4-aryl-but-3-en-2-ones **(1, 2)** at 61–90% yields. Subsequently, reactions of the resulting β-enaminoketones with an appropriate source of boron (BF_3_.OEt_2_) gave the corresponding oxazaborinine derivatives **(6, 7)** at 50–91% yields. UV-Vis and emission properties of biomolecule-binding properties for the DNA of these new BF_2_-β-enamino containing CCl_3_ units were also evaluated. Some compounds from the present series also exhibited potent antimicrobial effects on various pathogenic microorganisms at concentrations below those that showed cytotoxic effects. Compounds **4d, 4e, 6e**, and **6f** showed the best results and are very significant against *P. zopfii*, which causes diseases in humans and animals.

**Graphical Abstract:**
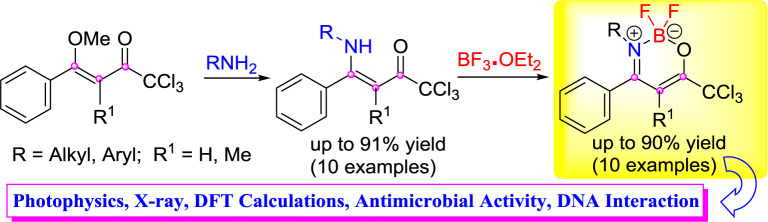


## Introduction

**Scheme 1:**
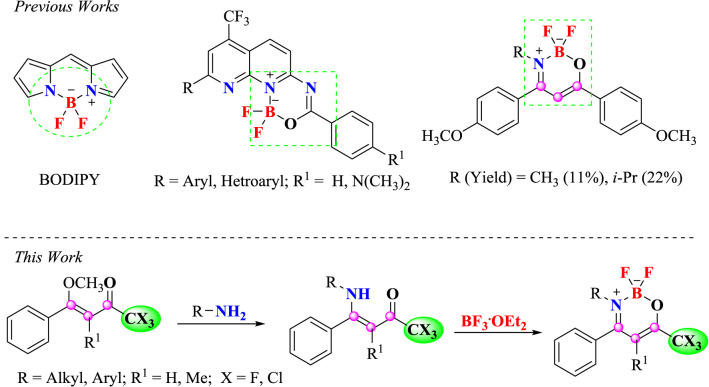
Structure of BODIPY and nitrogenated heterocyclic containing a BF_2_ core, examples of β-iminoenolate boron complexes and the synthetic route for β-enamino trihalomethyl ketones and the corresponding oxazaborinine derivatives.

Unsaturated heterocyclic compounds containing a boron atom have increased interest in recent years as a result of their potential in basic research and applications (Loudet and Burgess, 2007). Stands out BODIPY core (4,4-difluoro-4-bora-3a-azonia-4a-aza-s-indacenes) due to your small absorption, sharp emission bands with strong peak intensities, high values fluorescent quantum yields (Buyukcakir et al., 2009; Nagai and Chujo, 2010; Cakmak et al., 2011; He et al., 2011; Niu et al., 2011; Sozmen et al., 2014) and antimicrobial activity (Tekdaş et al., 2016) (**Scheme 1**).

Indeed, these properties promote the use of these structures in several applications such as, light emitting diodes (Wang et al., 2013) and solar cells (Kolemen et al., 2010). Nevertheless, besides structure of the BODIPY have high planarity, also shows very small Stokes’ shifts (Zhang et al., 2008; Kubota et al., 2010). So, synthesis novel boron analogous compounds with designed structural variations are relevant for interesting fluorescence materials (Poon et al., 2010).

Recently, our research group developed several difluoro-organoboron analogues. Nitrogenated heterocyclic scaffolds such as pyridines, (Bonacorso et al., 2018a). pyrimidines (Bonacorso et al., 2019), naphthyridines (Bonacorso et al., 2016), quinoxalines (Calheiro, 2018) have been studied for the synthesis of analogous novel structures (**Scheme 1**).

On the other hand, β-enaminoketones are the isosteric analogues of 1,3-enolic ketones and have been used in recent years as N,O double-dentate ligands to form 1,3,2-oxazaborines. These difluoroboron complexes of β-enaminones, which belong to the family of β-iminoenolate boron complexes (Kubota et al., 2011), have attracted attention as a promising class of fluorophores (Josefik et al., 2012).

Shankarling et al. described the synthesis and spectral and electrochemical characterization of some boron difluoride complexes of benzoindoline-based β-enaminones (Kumbhar et al., 2015). In 2008, Xia et al. reported excellent solution-state fluorescence for some heterocyclic β-iminoenolates (Xia et al., 2008). Later in 2013, Yoshii et al. described the synthesis of boron-ketoiminate derivatives that showed aggregation-induced emission properties and were prepared by the reactions of β-enaminoketones derivatives with boron trifluoride–diethyl etherate (BF_3_.Et_2_O) (**Scheme 1**) (Yoshii et al., 2013).

On the other hand, β-enaminoketones are interesting and easy obtainable synthetic intermediary. Enaminoketones have received attention due to their ambident nucleophilicity characteristics of amines, else with the ambident electrophilicity of enones (Bonacorso et al., 2002a; Bonacorso et al., 2010; Bonacorso et al., 2013).

β-enaminoketones and β-enaminoketo-esters are synthesized through the condensation reactions between carbonyl compounds and primary or secondary amines acid-catalysed (Ferraz and Pereira, 2004; Ferraz and Gonçalo, 2007). Due to its acidic nature, citrus juice is considered a good catalyst. Recently, Marvi et al. reported an efficient and green method using citrus juice as natural catalyst (Marvi and Fekri, 2018).

β-enaminones they have been also used to prepare different important antibacterial, (Mahmud et al., 2011). anti-inflammatories (Michael et al., 2001), and antitumor agents (Boger et al., 1989). Moreover, β-enaminones are widely used in the preparation of γ-amino-alcohols, which are structural units present in various compounds with pharmacological properties and natural products (Harris and Braga, 2004). There are many protocols well-established for the preparation of the γ-amino-alcohols. Nevertheless, reduction of 1,3-difunctionalized unsaturated structures [β-enaminoketones (Barluenga et al., 1992; Bartoli et al., 1994; Bartoli et al., 2002), or β-aminoketones (Katritzky and Harris, 1990; Pilli et al., 1990)] in which they present nitrogen and oxygen are more frequently.

Moreover, trihalomethyl groups in heterocyclic structures may drastically amend their chemicals, physical and pharmacological characteristics. The construction of trihalomethylated structures is done through already halogenated building blocks (Martins et al., 1999; Bonacorso et al., 2000; Martins et al., 2004). Trihalomethylated β-enaminoketones have been synthetized from the reaction of β-alkoxyvinyl trihalomethyl ketones (Hojo et al., 1986; Gerus et al., 1991) or acetylenes (Linderman and Kirollos, 1990) with amines.

On this line, fluorinated natural compounds are present in several drug classes. The main progress refers to research in the area of steroids, alkaloids, nucleosides, macrolides, prostaglandins, and amino acids (Kumar et al., 2008). *Quinine* isolated from chinchona bark led successive and innovative class of antimalarials (Kumar et al., 2003) such as chloroquine, mefloquine, and primaquine. Another example is the fluorocorticoid and fluorouracil derivatives, these drugs are still clinically used (Bégué and Bonnet-Delpon, 2006).

Following our studies in this area of interest, we decided to investigate the synthesis of novel boron complexes from β-enaminoketones trihalomethyl that contain the CF_3_ or CCl_3_ group bonded at the 6-position, N-alkyl(aryl) substituents with electron-donating or electron-withdrawing effect at the 3-position and a phenyl group at the 4-position. Furthermore, in view of the biological potential of molecules containing trihalomethyl groups, the aim of this paper was also to report the antimicrobial screening and cytotoxicity analysis of the compounds synthetized here (**Scheme 1**). Moreover, UV-Vis and emission spectroscopy was employed and DNA-binding properties were investigated of these new BF_2_-β-enamino derivatives in the present work.

## Experimental

Unless otherwise indicated, all common reagents and solvents were used as obtained from commercial suppliers and without further purification. ^1^H and ^13^C NMR spectra were acquired in Bruker Avance III 400 MHz or Bruker Avance III 600 MHz spectrometers for one-dimensional experiments, with 5 mm sample tubes, 298 K, and digital resolution of 0.01 ppm, in CDCl_3_ as solvent, and using TMS as the internal reference. The ^19^F and ^11^B-NMR spectra were acquired in a Bruker Avance III (^19^F at 564 MHz and ^11^B at 192 MHz) equipped with a 5-mm PABBO probe, 5-mm sample tubes at 298 K, and digital resolution of 0.01 ppm, in CDCl_3_, and using CFCl_3_ and BF_3_·OEt_2_, respectively, as the external reference. All spectra can be found at the **Supplementary Information File**—**Figures S1**–**S30**.

All results were reported with the chemical shift (*δ*), multiplicity, integration, and coupling constant (Hz). The following abbreviations were used to explain multiplicities: s = singlet, d = doublet, t = triplet, q = quartet, m = multiplet, and dd = double doublet. All NMR chemical shifts were reported in parts per million and relative to the internal reference.

All melting points were determined using coverslips in a Microquímica MQAPF-302 apparatus and are uncorrected.

The CHN elemental analyses were performed in a Perkin–Elmer 2400 CHN elemental analyzer (University of São Paulo, Brazil).

High resolution mass spectra (HRMS) were obtained for all compounds in an LTQ Orbitrap Discovery mass spectrometer (Thermo Fisher Scientific). This hybrid system combines the LTQ XL linear ion trap mass spectrometer and an Orbitrap mass analyzer. Experiments were performed *via* direct infusion of the sample (flow: 10 µl min^-1^) in positive-ion mode using electrospray ionization. Elemental composition calculations for comparison were executed using the specific tool included in the Qual Browser module of Xcalibur (Thermo Fisher Scientific, release 2.0.7) software. The spectra can be found in the **Supplementary Information File**—**Figures S30**–**S33**.

Single crystals of compounds 4g and 6e were obtained by slow evaporation of EtOH at 25°C. Compounds **4g** and **6e** was measured using a Bruker D8 QUEST diffractometer using Cu Ka radiation (l = 1.54080 Å) with a KAPPA four-circle goniometer equipped with a PHOTON II CPAD area detector. Absorption corrections were performed using the multi-scan method. Anisotropic displacement parameters for non-hydrogen atoms were applied. Most hydrogen atom positions were calculated geometrically and refined using the riding model, although some hydrogen atoms were refined freely. The structure was solved and refined using the WinGX software package (Farrugia, 2012).

The structures were refined based on the full-matrix least-squares method using the SHELXL program (Sheldrick, 2008). The ORTEP projections of the molecular structures were generated using the ORTEP-3 program. Crystallographic information files (CIFs) for the novel structures were deposited at the Cambridge Crystallographic Data Centre (CCDC) under identification number 1810806 (4g) and 1855153 (6e). Crystallographic data can be observed in the ESI (**Supplementary Information File**).

Ultraviolet/visible absorption spectra were recorded using Shimadzu UV2600 spectrophotometer and dichloromethane (DCM), methanol (MeOH), and dimethyl sulfoxide (DMSO) as solvents and concentration solution at 10–5 M range. Steady-state emission fluorescence analysis of samples in the same solvents used in absorption analysis were measured with a Varian Cary 50 fluorescence spectrophotometer (emission; 300–700 nm range; slit 2.0 mm). Due to the absence of emission intensity of all derivatives in all solvents, it was not possible to determine fluorescence quantum yield values.

For DNA interactions, boron complexes titrations with calf-thymus DNA (CT-DNA) were performed by UV-vis analysis at room temperature in DMSO (2%)-Tris-HCl buffer mixture (pH 7.2) using DMSO stock solution of derivatives (10^-4^ M range). The DNA pair base concentrations of low molecular weight DNA from calf thymus (CT-DNA) were determined by UV-vis absorption spectroscopy using ϵ = 6.600 M^−1^cm^−1^ (per base pair) at λ_max_ = 260 nm. Derivatives solutions in DMSO with Tris-HCl were titrated with increasing concentrations of CT-DNA (ranging from 0 to 100 μM). The absorption spectra of the compounds were acquired in the wavelength range of 250–700 nm. The binding constants (K_b_) of derivatives were calculated according to the decay of the absorption bands of compounds using Equation 1 through a plot of [DNA]/(ϵ_a_ − ϵ_f_) *versus* [DNA],

(1) $$\frac{\left[\text{DNA}\right]}{\left|\left({\text{ϵ}}_{\text{a}}-{\text{ ϵ}}_{f}\right)\right|}=\;\frac{\left[\text{DNA}\right]}{\left|\left({\text{ϵ}}_{\text{b}}-{\text{ ϵ}}_{f}\right)\right|}+\;\frac{1}{{\text{K}}_{\text{b}}\left|\left({\text{ϵ}}_{\text{b}}-{\text{ ϵ}}_{f}\right)\right|}$$

where [DNA] is the concentration of DNA in the base pairs, ϵ_a_ is the extinction coefficient (A_obs_/[compound]), and ϵ_b_ and ϵ_f_ are the extinction coefficients of free and fully bound forms, respectively. In the plots of [DNA]/(ϵ_a_ − ϵ_f_) *versus* [DNA], Kb is given by the ratio of the slope for the interception.

Competitive EB-binding studies were performed using steady-state emission fluorescence assay with CT-DNA. Compounds were dissolved in DMSO and through the gradual addition of the stock solution of the boron complexes to the quartz cuvette (1.0 cm path length) containing ethidium bromide dye (EB, 2.0 × 10^-7^ M) and DNA (1.0 × 10^-5^ M) in a Tris-HCl pH 7.4 buffer solution. Derivative concentration ranged from 0 to 100 µM. All samples were excited at λ_exc_ = 510 nm and emission fluorescence spectra were recorded at the range of 550–800 nm, 5 min after each addition of the complex solution in order to allow incubation to occur (incubation time). The fluorescence quenching Stern-Volmer constants (K_SV_) of compounds were calculated according to the decay of the emission bands of EB-DNA using Equation 2 through a plot of F_0_/F versus [DNA],

(2) $$\frac{F_{0}}{F}=1+{\text{ K}}_{\text{sv}}[Q]=1+k_{q}\tau_{0}[Q]$$

where F0 and F are the steady-state fluorescence intensities of EB-DNA adduct in the absence and presence of each derivative, respectively. K_sv_ and *k*_q_ are the Stern-Volmer quenching constant and the bimolecular quenching rate constant, respectively. The [Q] and τ_o_ are the derivative concentration and fluorescence lifetime of EB-DNA without the quencher (23.0 × 10^-9^ s) (Nafisi et al., 2007).

The standard Gibbs free energy (ΔG°) of derivative-DNA complex was calculated from the values of binding constant (K_b_) using Equation 3:

(3) $$\Delta G^{\circ }=-\text{RT }\ln{\text{ K}}_{b}$$

### General Procedure for the Synthesis of (*Z*)-1,1,1-trichloro-4-alkyl(aryl)amino -4-phenylbut-3-en-2-ones (4a-i, 5e)

To magnetically stirred solutions of (*Z*)-1,1,1-trichloro-4-methoxy-4-phenylbut-3-en-2-one (**1**) (5 mmol) in ethanol (20 ml), the respective amines (**3a-i, 5e**) (1.5 eq, 7.5 mmol) were added and the resulting mixtures were refluxed for 24 h. After that, the solutions were cooled at –10°C, resulting in yellow or white solids. The solids were filtered under atmospheric pressure, washed with cold ethanol, and dried under reduced pressure.


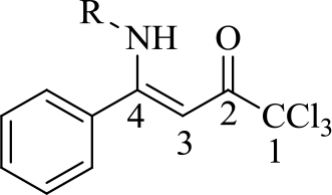


#### (Z)-1,1,1-Trichloro-4-(iso-propylamino)-4-phenylbut-3-en-2-one (4a)

Physical aspect: white solid. Yield: 61%. Melting point: 84–87°C.

^1^H NMR (400 MHz, CDCl_3_) δ: 10.65 (s, 1H, NH), 7.50–7.45 (m, 3H, Ph), 7.38–7.36 (m, 2H, Ph), 5.68 (s, 1H, H–3), 3.77–3.65 (m, 1H, CH), 1.23 (s, 3H, CH_3_), 1.21 (s, 3H, CH_3_). ^13^C NMR (101 MHz, CDCl_3_) δ: 180.5 (C-2), 169.1 (C-4), 134.9 (Ph), 130.0 (Ph), 128.7 (Ph), 127.1 (Ph), 97.2 (CCl_3_), 87.3 (C-3), 46.8 (CH), 23.9 (CH_3_). MS (EI, 70 eV), *m/z* (%) = 305 (M^+^, 5), 270 (1), 242 (9), 188 (100), 146 (19), 103 (14), 77 (7). Anal. Cal. for C_13_H_14_Cl_3_NO (306.62): C, 50.92; H, 4.60; N, 4.57. Found: C, 50.89; H, 4.40; N, 4.59.

#### (Z)-4-(Butylamino)-1,1,1-trichloro-4-phenylbut-3-en-2-one (4b)

Physical aspect: brown oil. Yield: 90%.

^1^H NMR (400 MHz, CDCl_3_) δ: 10.74 (s, 1H, NH), 7.49–7.45 (m, 3H, Ph), 7.39–7.36 (m, 2H, Ph), 5.75 (s, 1H, H–3), 3.26 (q, *J* = 13.0 Hz, 2H, CH_2_–N), 1.57 (quint, *J* = 14.7 Hz, 2H, CH_2_), 1.37 (sext, *J* = 14.5 Hz, 2H, CH_2_), 0.88 (t, *J* = 7.3 Hz, 3H, CH_3_). ^13^C NMR (101 MHz, CDCl_3_) δ: 180.6 (C-2), 170.2 (C-4), 134.6 (Ph), 130.1 (Ph), 128.7 (Ph), 127.4 (Ph), 97.2 (CCl_3_), 87.4 (C-3), 45.0 (CH_2_), 32.3 (CH_2_), 19.8 (CH_2_), 13.5 (CH_3_). MS (EI, 70 eV), *m/z* (%) = 319 (M^+^, 4), 256 (7), 202 (100), 184 (6), 103 (9). Anal. Cal. for C_14_H_16_Cl_3_NO (320.64): C, 52.44; H, 5.03; N, 4.37. Found: C, 52.46; H, 5.03; N, 4.44.

#### (Z)-1,1,1-Trichloro-4-phenyl-4-(phenylamino)but-3-en-2-one (4c)

Physical aspect: yellow solid. Yield: 76%. Melting point: 123–124°C.

^1^H NMR (400 MHz, CDCl_3_) δ: 12.02 (s, 1H, NH), 7.41–7.39 (m, Ph), 7.36–7.33 (m, Ph), 7.16 (tt, *J* = 7.6, *J* = 2 Hz, Ph), 7.07 (tt, *J* = 7.3, *J* = 1.3 Hz, Ph), 6.81 (dd, *J* = 8.5, *J* = 1.1 Hz, Ph), 6.04 (s, 1H, H–3). ^13^C NMR (101 MHz, CDCl_3_) δ: 181.7 (C-2), 165.7 (C-4), 138.3 (Ph), 134.6 (Ph), 130.4 (Ph), 128.9 (Ph), 128.7 (Ph), 128.4 (Ph), 125.5 (Ph), 123.7 (Ph), 96.9 (CCl_3_), 90.9 (C-3). MS (EI, 70 eV), *m/z* (%) = 339 (M^+^, 4), 269 (12), 222 (100), 180 (5), 77 (16). HRMS (ESI-TOF): C_16_H_12_Cl_3_NO (M + H), requires 340.0057. Found 340.0069.

#### (Z)-1,1,1-Trichloro-4-phenyl-4-(p-tolylamino)but-3-en-2-one (4d)

Physical aspect: yellow solid. Yield: 85%. Melting point: 112–113°C.

^1^H NMR (400 MHz, CDCl_3_) δ: 12.04 (s, 1H, NH), 7.42–7.32 (m, 4H, Ph), 6.95 (d, *J* = 8.2 Hz, Ph), 6.70 (d, *J* = 8.3 Hz, Ph), 6.02 (s, 1H, H–3), 2.25 (s, 3H, CH_3_). ^13^C NMR (101 MHz, CDCl_3_) δ: 181.5 (C-2), 165.8 (C-4), 135.6 (Ph), 135.4 (Ph), 134.7 (Ph), 130.3 (Ph), 129.5 (Ph), 128.6 (Ph), 128.4 (Ph), 123.6 (Ph), 97.0 (CCl_3_), 90.4 (C-3), 20.8 (CH_3_). MS (EI, 70 eV), *m/z* (%) = 353 (M^+^, 4), 319 (1), 236 (100), 91 (6). HRMS (ESI-TOF): C_17_H_14_Cl_3_NO (M + H), requires 354.0214. Found 354.0226.

#### (Z)-1,1,1-Trichloro-4-((4-methoxyphenyl)amino)-4-phenylbut-3-en-2-one (4e)

Physical aspect: yellow solid. Yield: 88%. Melting point: 144–145°C.

^1^H NMR (400 MHz, CDCl_3_) δ: 12.06 (s, 1H, NH), 7.41–7.32 (m, 5H, Ph), 6.76 (d, *J* = 9.0 Hz, 1H, Ph), 6.69 (d, *J* = 9.1 Hz, 1H, Ph), 6.68 (s, 1H, H–3), 3.73 (s, 3H, CH_3_). ^13^C NMR (101 MHz, CDCl_3_) δ: 181.5 (C-2), 166.0 (C-4), 157.5 (Ph), 134.7 (Ph), 131.2 (Ph), 130.3 (Ph), 128.6 (Ph), 128.4 (Ph), 125.3 (Ph), 114.2 (Ph), 97.0 (CCl_3_), 90.0 (C-3), 55.4 (CH_3_). MS (EI, 70 eV), *m/z* (%) = 369 (M^+^, 9), 334 (2), 252 (100), 209 (26). HRMS (ESI-TOF): C_17_H_14_Cl_3_NO_2_ (M + H), requires 370.0163. Found 370.0173.

#### (Z)-1,1,1-Trichloro-4-((4-nitrophenyl)amino)-4-phenylbut-3-en-2-one (4f)

Physical aspect: yellow solid. Yield: 65%. Melting point: 107–109°C.

^1^H NMR (400 MHz, CDCl_3_) δ: 11.83 (s, 1H, NH), 8.03 (d, *J* = 9.1 Hz, 1H, Ph), 7.53–7.49 (m, 1H, Ph), 7.44–7.37 (m, 4H, Ph), 6.85 (d, *J* = 9.0 Hz, 1H, Ph), 6.16 (s, 1H, H–3). ^13^C NMR (101 MHz, CDCl_3_) δ: 182.6 (C-2), 163.8 (C-4), 144.4 (Ph), 144.0 (Ph), 133.9 (Ph), 131.2 (Ph), 129.3 (Ph), 128.2 (Ph), 124.8 (Ph), 122.4 (Ph), 96.5 (CCl_3_), 94.1 (C-3). Anal. Cal. for C_16_H_11_Cl_3_N_2_O_3_ (385.63): C, 49.83; H, 2.88; N, 7.26. Found: C, 49.78; H, 2.85; N, 7.26.

#### (Z)-1,1,1-Trichloro-4-((4-fluorophenyl)amino)-4-phenylbut-3-en-2-one (4g)

Physical aspect: solid beige. Yield: 87%. Melting point: 115–116°C.

^1^H NMR (400 MHz, CDCl_3_) δ: 11.95 (s, 1H, NH), 7.42–7.30 (m, 5H, Ph), 6.88–6.77 (m, 4 H, Ph), 6.04 (s, 1 H, H–3). ^13^C NMR (101 MHz, CDCl_3_) δ: 181.9 (C-2), 165.8 (C-4), 160.3 (d, *J* = 246.4 Hz, Ph), 134.4 (d, *J* = 3.2 Hz, Ph), 134.4 (Ph), 130.5 (Ph), 128.8 (Ph), 128.4 (Ph), 125.5 (d, *J* = 8.2 Hz, Ph), 115.9 (d, *J* = 22.9 Hz, Ph), 96.9 (CCl_3_), 90.8 (C-3). MS (EI, 70 eV), *m/z* (%) = 358 (M^+^, 3), 294 (4), 269 (13), 240 (100), 198 (4), 95 (8). HRMS (ESI-TOF): C_16_H_11_Cl_3_FNO (M + H), requires 357.9963. Found 357.9976.

#### (Z)-4-((4-Bromophenyl)amino)-1,1,1-trichloro-4-phenylbut-3-en-2-one (4h)

Physical aspect: yellow solid. Yield: 80%. Melting point: 142–144°C.

^1^H NMR (400 MHz, CDCl_3_) δ: 11.92 (s, 1H, NH), 7.46–7.42 (m, 1H, Ph), 7.39–7.32 (m, 4H, Ph), 7.28 (d, *J* = 8.8 Hz, 2H, Ph), 6.67 (d, *J* = 8.7 Hz, 2H, Ph), 6.06 (s, 1H, H–3). ^13^C NMR (101 MHz, CDCl_3_) δ: 181.9 (C-2), 165.2 (C-4), 137.4 (Ph), 134.1 (Ph), 132.0 (Ph), 130.7 (Ph), 128.9 (Ph), 128.3 (Ph), 125.0 (Ph), 118.7 (Ph), 96.7 (CCl_3_), 91.5 (C-3).

MS (EI, 70 eV), *m/z* (%) = 418 (M^+^, 10), 301 (M^+^, 100), 301 (M^+2^, 20), 302 (M^+3^, 97), 269 (27), 271 (7), 234 (14), 221 (51), 193 (41), 192 (7), 165 (14), 102 (15), 76 (16). HRMS (ESI-TOF): C_16_H_11_BrCl_3_NO (M + H), C, 45.81; H, 2.64; N, 3.34. Found: C, 45.53; H, 2.54; N, 3.27.

#### (Z)-1,1,1-Trichloro-4-(naphthalen-2-ylamino)-4-phenylbut-3-en-2-one (4i)

Physical aspect: yellow solid. Yield: 85%. Melting point: 149–151°C.

^1^H NMR (400 MHz, CDCl_3_) δ: 12.39, 8.28 (d, *J* = 8.6 Hz, 1H), 7.85 (d, *J* = 8.1 Hz, 1H), 7.62 (ddd, *J* = 6.9, 4.7, 1.4 Hz, 2H), 7.55 (ddd, *J* = 8.0, 6.9, 1.2 Hz, 1H), 7.34–7.20 (m, 5 H, Ph), 7.12 (t, *J* = 7.9 Hz, 1H), 6.71 (d, *J* = 7.5 Hz, 1H), 6.19 (s, 1H, H–3). ^13^C NMR (101 MHz, CDCl_3_) δ: 182.1 (C-2), 167.1 (C-4), 134.7 (Ph), 134.3 (Ph), 134.0 (Ph), 130.3 (Ph), 128.6 (Ph), 128.4 (Ph), 128.0 (Ph), 127.2 (Ph), 126.7 (Ph), 126.5 (Ph), 124.9 (Ph), 123.2 (Ph), 121.9 (Ph), 96.9 (CCl_3_), 91.2 (C-3). MS (EI, 70 eV), *m/z* (%) = 391 (M^+^, 7), 355 (2), 290 (3), 272 (100), 273 (21), 244 (9), 166 (4), 127 (14), 77 (5). HRMS (ESI-TOF): C_20_H_14_Cl_3_NO (M + H), requires 390.0217. Found 390.0214.

#### (Z)-1,1,1-Trichloro-4-((4-methoxyphenyl)amino)-3-methyl-4-(p-tolyl)but-3-en-2-one (5e)

Physical aspect: yellow solid. Yield: 80%. Melting point: 115–116°C.

^1^H NMR (400 MHz, CDCl_3_) δ: 13.11 (s, 1H, NH), 7.14 (d, *J* = 7.9 Hz, 1H), 7.05 (d, *J* = 8.0 Hz, 1H), 6.66 (d, *J* = 9.0 Hz, 1H), 6.61 (d, *J* = 9.1 Hz, 1H), 3.70 (s, 3H, OCH_3_), 2.34 (s, 3H, PhCH_3_), 1.95 (s, 3H, CH_3_). ^13^C NMR (100 MHz, CDCl_3_) δ: 179.7 (C-2), 168.2 (C-4), 156.9 (Ph), 139.1 (Ph), 131.9 (Ph), 131.3 (Ph), 129.3 (Ph), 128.6 (Ph), 125.4 (Ph), 113.9 (Ph), 98.8 (CCl_3_), 94.8 (C-3), 55.3 (OCH_3_), 21.3 (CH_3_), 16.8 (CH_3_). Anal. Cal. for C_19_H_18_Cl_3_NO_2_ (385.63): C, 57.24; H, 4.55; N, 3.51. Found: C, 57.18; H, 4.57; N, 3.51.

### General Procedure for the Synthesis of 2,2-difluoro-3-alkyl(aryl)-4-phenyl-6-(trichloromethyl)-2*H*-1,3,2-oxazaborinin-3-ium-2-uides (6a-i, 7e):

In 50 ml round bottom flasks equipped with reflux condenser and drying tube, the respective (*Z*)-1,1,1-trichloro-4-alkyl(aryl)amino-4-phenylbut-3-en-2-ones (**4a-i**, **5e**) (1 mmol) were dissolved in anhydrous CHCl_3_ (15 ml), followed by the addition of BF_3_·OEt_2_ (1 ml of 48% ether sol.) and anhydrous Et_3_N (1 ml). The mixtures were stirred at temperature of reflux for 18 h, then diluted with CHCl_3_, washed with water (3× 20 ml) and the organic phase was dried with Na_2_SO_4_, filtered, and then the solvent (CHCl_3_) was removed under reduced pressure. The resulting oils were solubilized in EtOH (15 ml) at ambient temperature and stored overnight in the freezer for precipitation of the products **6a-i** and **7e**. The resulting solids were filtered under reduced pressure and washed with cold ethanol. The pure products **6** and **7** were obtained at 50–91% yields.


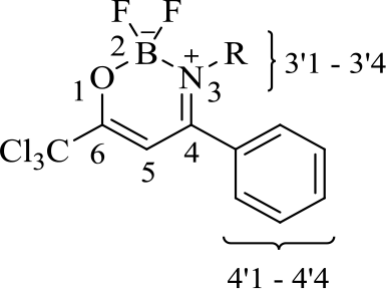


#### 2,2-Difluoro-3-isopropyl-4-phenyl-6-(trichloromethyl)-2H-1,3,2-oxazaborinin-3-ium-2-uide (6a)

Physical aspect: brown solid. Yield: 74%. Melting point: 121–123°C.

^1^H NMR (400 MHz, CDCl_3_) δ: 7.59–7.57 (m, 3H, Ph), 7.34–7.32 (m, 2H, Ph), 6.23 (s, 1H, H–5), 4.19–4.14 (m, 1H, CH), 1.44 (t, *J* = 1.3 Hz, 1H, CH_3_), 1.42 (t, *J* = 1.3 Hz, 1H, CH_3_). ^13^C NMR (101 MHz, CDCl_3_) δ: 172.30 (C-4), 165.39 (C-6), 134.20 (C-4’1), 130.79 (C-4’4), 129.41 (C-4’2), 125.74 (C-4’3), 96.86 (C-5), 91.70 (CCl_3_), 54.20 (CH), 21.83 (CH_3_). ^11^B NMR (193 MHz, CDCl_3_) δ: 1.24 (t, *J* = 17.5 Hz). ^19^F NMR (565 MHz, CDCl_3_) δ: −134.64 – −134.99 (m). Anal. Cal. For C_13_H_13_BCl_3_F_2_NO (354.42): C, 44.06; H, 3.70; N, 3.95. Found: C, 44.08; H, 3.85; N, 4.02.

#### 3-Butyl-2,2-difluoro-4-phenyl-6-(trichloromethyl)-2H-1,3,2-oxazaborinin-3-ium-2-uide (6b)

Physical aspect: brown solid. Yield: 80%. Melting point: 85–86°C.

^1^H NMR (400 MHz, CDCl_3_) δ: 7.60–7.55 (m, 3H, Ph), 7.38–7.36 (m, 2H, Ph), 6.29 (s, 1H, H–5), 3.58 (t, 2H CH_2_), 1.73 (quint, 2H, CH_2_), 1.19 (sext, 2H, CH_2_), 0.79 (t, 3H, CH_3_). ^13^C NMR (101 MHz, CDCl_3_) δ: 172.7 (C-4), 165.7 (C-6), 133.3 (C-4’1), 131.1 (C-4’4), 129.3 (C-4’2), 126.3 (C-4’3), 96.4 (C-5), 91.8 (CCl_3_), 48.8 (NCH_2_), 32.3 (CH_2_), 20.1 (CH_2_), 13.4 (CH_3_). ^11^B NMR (193 MHz, CDCl_3_) δ: 1.13 (t, *J* = 15.8 Hz). ^19^F NMR (565 MHz, CDCl_3_) δ: −138.13 – −138.36 (m). Anal. Cal. For C_14_H_15_BCl_3_F_2_NO (368.44): C, 45.64; H, 4.10; N, 3.80. Found: C, 46.16; H, 3.84; N, 3.93.

#### 2,2-Difluoro-3,4-diphenyl-6-(trichloromethyl)-2H-1,3,2-oxazaborinin-3-ium-2-uide (6c)

Physical aspect: yellow solid. Yield: 65%. Melting point: 198–199°C.

^1^H NMR (400 MHz, CDCl_3_) δ: 7.41–7.14 (m, 10H, Ph), 6.60 (s, 1H, H–5). ^13^C NMR (101 MHz, CDCl_3_): 172.3 (C-4), 167.9 (C-6), 139.6 (C-3’1), 133.5 (C-4’1), 131.5 (C-4’4), 129.0 (C-4’2),128.7 (Ph), 128.6 (Ph), 128.2 (Ph), 126.4 (Ph), 96.3 (C-5), 91.8 (CCl_3_). ^11^B NMR (193 MHz, CDCl_3_) δ: 1.34 (t, *J* = 12.7 Hz). ^19^F NMR (565 MHz, CDCl_3_) δ: −132.97 – −134.25 (m). Anal. Cal. For C_16_H_11_BCl_3_F_2_NO (388.43): C, 49.47; H, 2.85; N, 3.61. Found: C, 49.41; H, 2.96; N, 3.61.

#### 2,2-Difluoro-4-phenyl-3-(p-tolyl)-6-(trichloromethyl)-2H-1,3,2-oxazaborinin-3-ium-2-uide (6d)

Physical aspect: yellow solid. Yield: 70%. Melting point: 173–175°C.

^1^H NMR (600 MHz, CDCl_3_) δ: 7.39 (t, *J* = 7.4 Hz, 1H, Ph), 7.31 (t, *J* = 7.7 Hz, 2H, Ph), 7.23 (d, *J* = 7.7 Hz, 2H, Ph), 7.04 (q, *J* = 8.4 Hz, 4H, Ph), 6.58 (s, 1H, H–5), 2.28 (s, 3H, CH_3_). ^13^C NMR (151 MHz, CDCl_3_) δ: 172.0 (C-4), 167.7 (C-6), 138.3 (C-3’1), 137.1 (C-3’4), 133.7 (C-4’1), 131.4 (C-4’4), 129.6 (C-4’2), 128.7 (Ph), 128.6 (Ph), 126.1 (C-4’3), 96.3 (C-5), 91.9 (CCl_3_), 21.0 (CH_3_). ^11^B NMR (193 MHz, CDCl_3_) δ: 1.31 (t, *J* = 12.4 Hz). ^19^F NMR (565 MHz, CDCl_3_) δ: −133.84 – −133.90 (m). Anal. Cal. For C_17_H_13_BCl_3_F_2_NO (402.46): C, 50.73; H, 3.26; N, 3.48. Found: C, 50.57; H, 3.32; N, 3.52.

#### 2,2-Difluoro-3-(4-methoxyphenyl)-4-phenyl-6-(trichloromethyl)-2H-1,3,2-oxazaborinin-3-ium-2-uide (6e)

Physical aspect: yellow solid. Yield: 91%. Melting point: 152–153°C.

^1^H NMR (400 MHz, CDCl_3_) δ: 7.42–7.38 (m, 1H, Ph), 7.35–7.30 (m, 2H, Ph), 7.25–7.22 (m, 2H, Ph), 7.06 (d, *J* = 9.0 Hz, 1H, Ph), 6.76 (d, *J* = 9.1 Hz, 1H, Ph), 6.58 (s, 1H, H–5), 3.75 (s, 3H, CH_3_). ^13^C NMR (101 MHz, CDCl_3_) δ: 171.8 (C-4), 167.5 (C-6), 159.1 (C-3’4), 133.7 (C-4’1), 132.4 (C-3’1), 131.3 (C-4’4), 128.8 (C-4’2), 128.6 (C-3’2), 127.4 (C-4’3), 114.2 (C-3’3), 96.3 (C-5), 91.8 (CCl_3_), 55.4 (OCH_3_). ^11^B NMR (193 MHz, CDCl_3_) δ: 1.29 (t, *J* = 12.2 Hz). ^19^F NMR (565 MHz, CDCl_3_) δ: −131.70 – −136.45 (m). Anal. Cal. For C_17_H_13_BCl_3_F_2_NO_2_ (418.46): C, 48.79; H, 3.13; N, 3.35. Found: C, 48.64; H, 3.22; N, 3.24.

#### 2,2-Difluoro-3-(4-nitrophenyl)-4-phenyl-6-(trichloromethyl)-2H-1,3,2-oxazaborinin-3-ium-2-uide (6f)

Physical aspect: yellow solid. Yield: 60%. Melting point: 201–202°C.

^1^H NMR (400 MHz, CDCl_3_) δ: 8.13 (d, *J* = 9.2 Hz, 1H, Ph), 7.48–7.43 (m, 1H, Ph), 7.38–7.33 (m, 4H, Ph), 7.23 (dd, *J* = 8.4, 1.3 Hz, 1H), 6.66 (s, 1H, H–5). ^13^C NMR (101 MHz, CDCl_3_) δ: 173.6 (C-4), 169.7 (C-6), 147.1 (C-3’1), 145.3 (C-3’4), 132.9 (C-4’1), 132.3 (C-4’4), 129.3 (C-4’3), 128.6 (C-3’2), 127.8 (C-4’2), 124.4 (C-3’3), 96.5 (C-5), 91.6 (CCl_3_). ^11^B NMR (193 MHz, CDCl_3_) δ: 1.30. ^19^F NMR (565 MHz, CDCl_3_) δ −132.18 – −132.59 (m). Anal. Cal. For C_16_H_10_BCl_3_F_2_N_2_O_3_ (433.43); C, 44.34; H, 2.33; N, 6.46. Found: C, 44.33; H, 2.20; N, 6.38.

#### 2,2-Difluoro-3-(4-fluorophenyl)-4-phenyl-6-(trichloromethyl)-2H-1,3,2-oxazaborinin-3-ium-2-uide (6g)

Physical aspect: yellow solid. Yield: 67%. Melting point: 169–170°C.

^1^H NMR (400 MHz, CDCl_3_) δ: 7.45–7.40 (m, 1H, Ph), 7.36–7.32 (m, 2H, Ph), 7.23–7.21 (m, 2H, Ph), 7.15–7.12 (m, 2H, Ph), 6.99–6.94 (m, 2H, Ph), 6.60 ^13^C NMR (101 MHz, CDCl_3_) δ: 172.7 (C-4), 168.1 (C-6), 161.9 (d, *J* = 249.4 Hz, C-3’4), 135.6 (d, *J* = 3.1 Hz, C-3’1), 133.3 (C-4’1), 131.6 (C-4’4), 128.9 (C-4’2), 128.5 (C-4’3), 128.2 (d, *J* = 8.6 Hz, C-3’2), 116.1 (d, *J* = 23.1 Hz, C-3’3), 96.3 (C-5), 91.7 (CCl_3_). ^11^B NMR (193 MHz, CDCl_3_) δ: 1.28 (t, *J* = 12.5 Hz). ^19^F NMR (565 MHz, CDCl_3_) δ: −112.39 (s); −133,37 – −133,43 (m). Anal. Cal. For C_16_H_10_BCl_3_F_3_NO (406.42): C, 47.28; H, 2.48; N, 3.45. Found: C, 46.97; H, 2.30; N, 3.59.

#### 3-(4-Bromo-phenyl)-2,2-difluoro-4-phenyl-6-(trichloromethyl)-2H-1,3,2-oxazaborinin-3-ium-2-uide (6h)

Physical aspect: yellow solid. Yield: 50%. Melting point: 174–175°C.

^1^H NMR (400 MHz, CDCl_3_) δ: 7.46–7.33 (m, 5H, Ph), 7.23 (d, *J* = 7.3 Hz, 1H, Ph), 7.03 (d, *J* = 8.6 Hz, 1H, Ph), 6.61 (s, 1H, H–5). ^13^C NMR (101 MHz, CDCl_3_) δ: 172.6 (C-4), 168.4 (C-6), 138.7 (C-3’1), 133.2 (C-4’1), 132.3 (C-4’4), 131.8 (C-3’4), 129.0 (C-4’2), 128.6 (C-4’3), 128.0 (C-3’2), 122.3 (C-3’3), 94.7 (C-5), 91.7 (CCl_3_). ^11^B NMR (193 MHz, CDCl_3_) δ 1.25 (t, *J* = 12.0 Hz). ^19^F NMR (565 MHz, CDCl_3_) δ: −133.21 (s). Anal. Cal. For C_16_H_10_BBrCl_3_F_2_NO (467.33): C, 41.12; H, 2.16; N, 3.00. Found; C, 41.29; H, 2.00; N, 3.05.

#### 2,2-Difluoro-3-(naphthalen-2-yl)-4-phenyl-6-(trichloromethyl)-2H-1,3,2-oxazaborinin-3-ium-2-uide (6i)

Physical aspect: yellow solid. Yield: 74%. Melting point: 197–198°C.

^1^H NMR (400 MHz, CDCl_3_) δ: 7.77–7.74 (m, 3H, Ph), 7.49–7.37 (m, 4H, Ph), 7.21–7.17 (m, 3H, Ph), 7.10–7.06 (m, 2H, Ph), 6.69 (s, 1H, H–5). ^13^C NMR (101 MHz, CDCl_3_) δ: 174.9 (C-4), 168.5 (C-6), 135.9 (Ph), 133.8 (Ph), 133.5 (Ph), 131.4 (Ph), 129.0 (Ph), 128.6 (Ph), 128.3 (Ph), 128.2 (Ph), 127.4 (Ph), 127.2 (Ph), 126.5 (Ph), 125.0 (Ph), 124.7 (Ph), 122.8 (Ph), 96.2 (C-5), 91.9 (CCl_3_). ^11^B NMR (193 MHz, CDCl_3_) δ: 1.75–1.16 (m). ^19^F NMR (565 MHz, CDCl_3_) δ: −132.66 – −137.64 (m). Anal. Cal. For C_20_H_13_BCl_3_F_2_NO (438.49): C, 54.78; H, 2.99; N, 3.19. Found; C, 54.72; H, 3.17; N, 3.31.

#### 2,2-Difluoro-3-(4-methoxyphenyl)-5-methyl-4-(p-tolyl)-6-(trichloromethyl)-2H-1,3,2-oxazaborinin-3-ium-2-uide (7e)

Physical aspect: yellow solid. Yield: 69%. Melting point: 171–173°C.

^1^H NMR (400 MHz, CDCl_3_) δ: 7.10 (d, *J* = 8.0 Hz, 2H, Ar), 6.95–6.91 (m, 4H, Ar), 6.69 (d, *J* = 8.9 Hz, 2H, Ar), 3.72 (s, 3H, OCH_3_), 2.30 (s, 3H, PhCH_3_), 1.96 (s, 3H, CH_3_). ^13^C NMR (100 MHz, CDCl_3_) δ: 177.0 (C-4), 164.1 (C-6), 158.7 (C-3’4), 140.3 (C-3’1), 133.0 (C-4’1), 130.0 (C-4’4), 129.3 (C-4’2), 127.9 (C-4’3), 127.2 (C-3’2), 113.9 (C-3’3), 103.4 (C-5), 94.0 (CCl_3_), 55.3 (q, *J* = 6.6 Hz, OCH_3_), 21.3 (q, *J* = 4.9 Hz, PhCH_3_). 16.7 (CH_3_). ^11^B NMR (193 MHz, CDCl_3_) δ: 0.66 – 0.53 (m). ^19^F NMR (565 MHz, CDCl_3_) δ: −138.63 – −138.74 (m). Anal. Cal. For C_19_H_17_BCl_3_F_2_NO_2_ (446.51): C, 51.11; H, 3.84; N, 3.14. Found; C, 51.11, H, 3.87, N, 3.12.

## Results and Discussion

### Synthesis

The starting materials (*Z*)-1,1,1-trichloro-4-methoxy-4-phenylbut-3-en-2-one (1) and (*Z*)-1,1,1-trichloro-4-methoxy-3-methyl-4-(*p*-tolyl)but-3-en-2-one (**2**) were synthetized according to the methodology reported by our group in previous works. Compounds **1** and **2** were synthesized in two steps from reactions of acetophenone or propiophenone using an excess amount of trimethyl orthoformate. Finally, the acylation reaction of the dimethyl acetal intermediates with trichloroacetyl chloride conducted to the compounds **1** and **2** (Colla et al., 1999; Bonacorso et al., 1999; Martins et al., 2004).

**Scheme 2:**
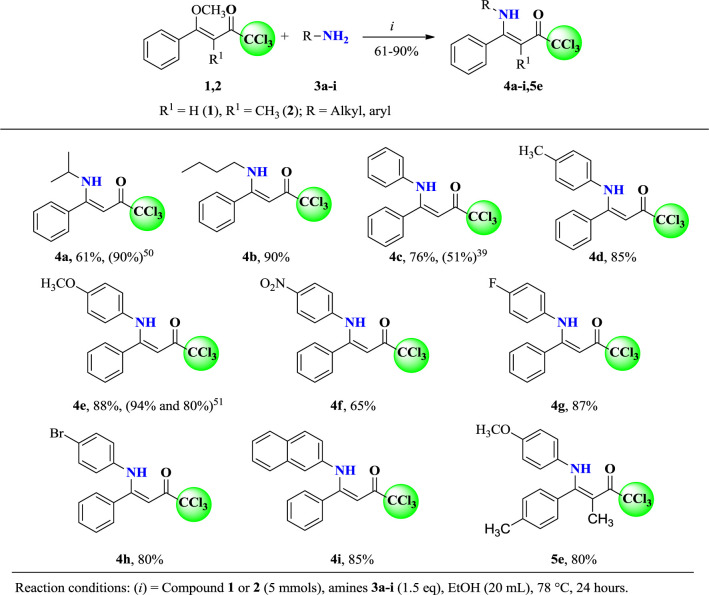
Synthesis of (*Z*)-1,1,1-trichloro-4-alkyl(aryl)amino-4-phenylbut-3-en-2-ones (**4a-i** and **5e**).

In order to obtain a novel series of (*Z*)-1,1,1-trichloro-4-alkyl(aryl)amino)-4-phenylbut-3-en-2-ones (**4a-e** and **5e**), the methodology described in the literature was employed because the O,N-exchange reaction was already well established by our research group. In the present work, we reacted (*Z*)-1,1,1-trichloro-4-methoxy-4-phenylbut-3-en-2-one (**1**) (5 mmol) and several amines (**3a-i**) (7.5 mmol) at a molar ratio of 1:1.5, in ethanol (20 ml) for 24 h at reflux temperature (**Scheme 2**), according to similar procedures described in the literature (Bonacorso et al., 2002b; Martins et al., 2007).

The products **4** and **5** were obtained by simple precipitation at low temperature. Subsequently, the products were filtered, washed with cold ethanol, and dried under reduced pressure. This procedure allowed to isolate **4a-i** and **5e** in 61 to 90% of yield (**Scheme 2**). Compounds **4a** (Sosnovskikh et al., 2002), **4c** (Hojo et al., 1986), and **4e** (Toshiaki and Minoru, 1977)). are already described in the literature, but they were obtained through different precursors and procedures.

In order to explore the synthetic potential of 4a-i and 5e, (Z)-1,1,1-trichloro-4-phenyl-4-(*p*-tolylamino)but-3-en-2-one (**4d**) was employed to evaluate the influence of different reaction conditions such as solvent, temperature, time, and volume of BF_3_.OEt_2_ or Et_3_N to obtain the oxazaboron complexes **6**, **7** (**Table 1**).

**Table 1:** Optimization of the reaction condition for the synthesis of 2,2-difluoro-3-*p*-tolyl-4-phenyl-6-(trichloromethyl)-2*H*-1,3,2-oxazaborinin-3-ium-2-uide (**6d**).

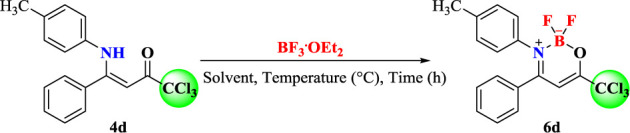						
Entry	Solvent	BF_3_.OEt_2_ (ml)	Et_3_N (ml)	Temp. (°C)	Time (h)	Yield 6d (%)
1	CHCl_3_	4	4	r.t.	24 or 48	[a]
2	CHCl_3_	4	4	61	18	68
3	CH_2_Cl_2_	4	4	40	24	[a]
4	DCE	4	4	83	24	46
5	CHCl_3_	2	2	61	18	63
6	CHCl_3_	1	1	61	18	65
7	CHCl_3_	1	–	61	18	[a]

Reaction condition: (Z)-1,1,1-trichloro-4-phenyl-4-(p-tolylamino)but-3-en-2-one (**4d**) (1 mmol), Solvent (15 ml), anhydrous system. [a] Recovery of starting material.

Optimization of the reaction began based on the methodology described by Bonacorso et al. in 2016. The reaction employs 1 mmol of the ligand precursor, 15 ml of anhydrous CHCl_3_, 4 ml of BF_3_.OEt_2_ (~16 mmol), and 4 ml of Et_3_N (~28 mmol) at room temperature, but this attempt did not lead to product formation after 24–48 h (**Table 1** – Entry 1). The reactions were monitored by TLC. However, under the same conditions at the reflux temperature of CHCl_3_, product **6d** was formed in 68% yield after 18 h of reaction (**Table 1** – Entry 2). Based on the work described by Yoshii et al. in 2013 (Yoshii et al., 2013), the reaction was refluxed in anhydrous CH_2_Cl_2_ and the desired product was not obtained after 24 h of reaction (**Table 1** – Entry 3). In this condition, a higher reflux temperature solvent was evaluated, which was ClCH_2_CH_2_Cl (DCE), although the result was only 46% yield (**Table 1** – Entry 4).

From these results, the volume of BF_3_.OEt_2_ and Et_3_N for the reaction was also evaluated. By reducing the volume of BF_3_.OEt_2_ and Et_3_N from 4 to 2 ml, 63% yield of **6d** was observed after 18 h (**Table 1** – Entry 5). The reaction was still efficient and led to 65% yield (**Table 1** – Entry 6) when the volumes of BF_3_.OEt_2_ and Et_3_N were reduced to 1 ml each. No formation of product was observed in the absence of triethylamine as base (**Table 1** – Entry 7).

Thus, the results obtained by the optimization study showed that the best condition for the synthesis of the boron complexes (**6a-i** and **7e**) was with 1 mmol of β-enaminoketone, 1 ml of Et_3_N (~7 mmol), and 1 ml of BF_3_.OEt_2_ (~4 mmol) in 15 ml of anhydrous CHCl_3_ under reflux for 18 h (**Table 1** – Entry 6).

**Scheme 3:**
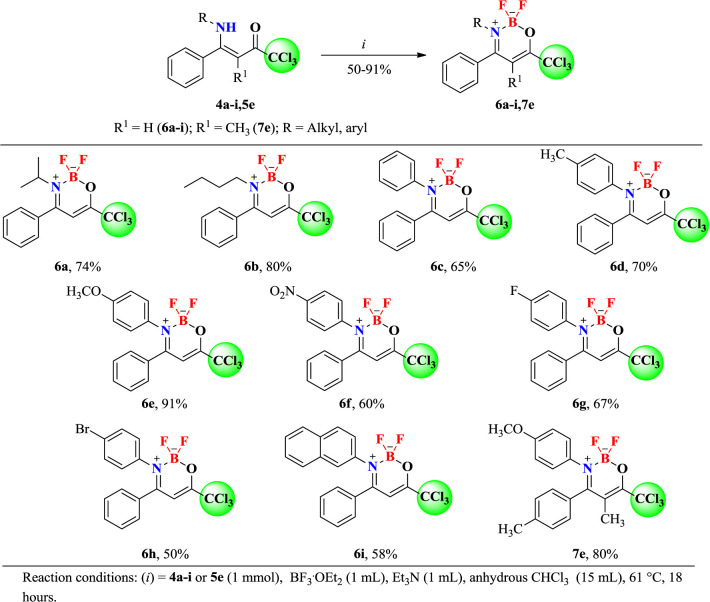
Synthesis of 2,2-difluoro-3-alkyl(aryl)amino-4-phenyl-6-(trichloromethyl)-2*H*-1,3,2-oxazaborinin-3-ium-2-uide (**6a-i** and **7e**).

After the reaction optimization, we investigate the substrate scope of (*Z*)-1,1,1-trichloro-4-alkyl(aryl)amino-4-phenylbut-3-en-2-ones (**4a-i** and **5e**) in order to expand the structural diversity of the corresponding boron complexes 6a-i, 7e (**Scheme 3**). As shown in **Scheme 3**, the (*Z*)-1,1,1-trichloro-4-alkyl(aryl)amino-4-phenylbut-3-en-2-ones (**4a-i** and **5e**) were submitted to target BF_2_-coordination, where the alkyl and aryl derivatives furnished the desired products 6a-i and 7e in moderate to excellent yields (50–91%).

Furthermore, 4-*N*-alkyl-substituted β-enaminoketones such as *N*-*iso*-propyl **6a** (74%) and *N*-butyl 6b (80%), which were isolated in good yields, were also successfully included in the reaction scope. Similar conditions were also found for 4-substituted aryl-amines, including aniline **6c** (65%) and 2-naphthylamine **6i** (58%). Electron-donating 4-methylphenylamino and 4-methoxyphenylamino substituents from anilines **3d** and **3e** performed very well with this procedure and the desired complexes 6d and 6e were obtained in 70 and 91% yields, respectively. Electron-withdrawing groups, such as 4-nitrophenylamino from aniline **3f**, also afforded the respective coordination complex **6f** in 60% yield. 4-phenylhalogenated β-enaminoketones were also submitted to the target BF_2_-coordination. Thus, derivatives of 4-fluoro- and 4-bromo-substituted anilines presented good reactivities and allowed also the isolation of complexes **6g** (67%) and **6h** (50%).

**Scheme 4:**
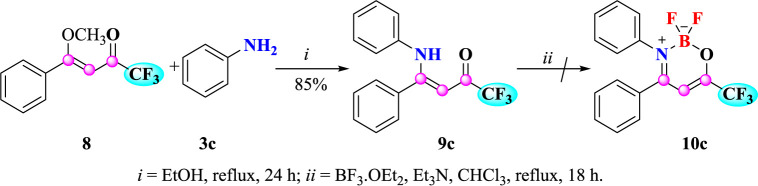
Attempt to synthesize 2,2-difluoro-3,4-diphenyl-6-(trifluoromethyl)-2*H*-1,3,2-oxazaborinin-3-ium-2-uide (**10c**).

In view of the differentiated properties that the trifluoromethyl substituent confers in various molecules in both chemical materials and medicinal chemistry, we also synthesized an example of substituted trifluoromethyl β-enaminoketone **9c** to study its reactivity against BF_3_.OEt_2_ (**Scheme 4**). Compound **9c** was synthesized using the methodology described using (*Z*)-1,1,1-trifluoro-4-methoxy-4-phenylbut-3-en-2-one (8) with aniline (**3c**). The compound **9c** was obtained after recrystallization in 85% yield.

In order to investigate the synthetic reactivity of trifluoromethyl β-enaminoketone **9c** against BF_2_.OEt_2_ complexation, the optimized reaction condition carried out for the (*Z*)-1,1,1-trichloro-4-alkyl(aryl)amino-4-phenylbut-3-en-2-ones (**4a-i** and **5e**) was attempted for **9c**. However, the formation of trifluoromethylated complex 10c was not possible because the high electron-withdrawing effect of the trifluoromethyl group at position 6, which hinders a stable N-B-O coordination.

### Structural Elucidation

The new structures of **4a-i**, **5e**, 6a-i, and **7e** were confirmed and characterized by ^1^H-, ^13^C-, ^11^B-, and ^19^F-NMR spectroscopy, CHN elemental analysis or high-resolution mass spectra (HRMS).

The structures of compounds **4a-i**, **5e** were deduced on the basis of the NMR data of other β-enaminones previously synthesized in literature.^50,51^ The ^1^H NMR chemical shifts of the β-enaminoketone hydrogens (NH) for **4a-i**, **5e** showed chemical shifts at an average of 11.87 ppm, which suggests to us that the compounds **4a-i**, **5e** are in the *Z*,*Z*-conﬁguration in solution (CDCl_3_), which is favored by an hydrogen interaction (N–H···OC). The vinyl hydrogen (H-3) was observed in the form of a singlet at an average of 6.06 ppm for all compounds **4**, **5**. The ^13^C NMR spectra of all compounds **4a-i** and **5e** showed, on average, chemical shifts at 181.4 ppm C-2, 166.7 ppm for C-4, 90.8 ppm for C-3, and 97.1 ppm for CCl_3_.

A comparison between the ^1^H-NMR data of compounds **4a-i**, **5e** and **6a-i**, **7e** showed clearly that complexes **6a-i** and **7e** do not show chemical shift signals (N–H) around at 12 ppm. Furthermore, an alteration in the chemical shifts of the H-3 signals from 6.06 ppm in average for the β-enaminones **4**, **5** to 6.53 ppm in the boron complexes 6, 7 was also observed.

Upon comparing the ^13^C NMR data of the β-enaminone precursors **4a-i**, **5e** and the boron complexes **6a-i**, **7e**, the major evidence for the formation of **6a-i**, **7e** was the alteration in the chemical shifts at average of the carbons C-4, C-6, CCl_3_, and C-5 for 173.1, 167.3, 96.9, and 92.0 ppm, respectively.

Moreover, in order to demonstrate boron complex formation, ^19^F- and ^11^B-NMR experiments were also performed. ^19^F-NMR spectra of compounds **6a-i**, **7e** were performed using chloroform-*d* and, the signals were observed in the form of a multiplet for the compounds in the range of -131.7 ppm to -138.7 ppm. For the ^11^B-NMR spectra, the chemical shifts were observed in the form of a triplet at 1.26 ppm on average for each compound due to the coupling of both ^19^F nuclei. However, for compounds 6a-i and 7e, the ^19^F-NMR spectra showed a multiplet for each compound at -134.1ppm and -135.59 ppm, respectively.

**Figure 1:**
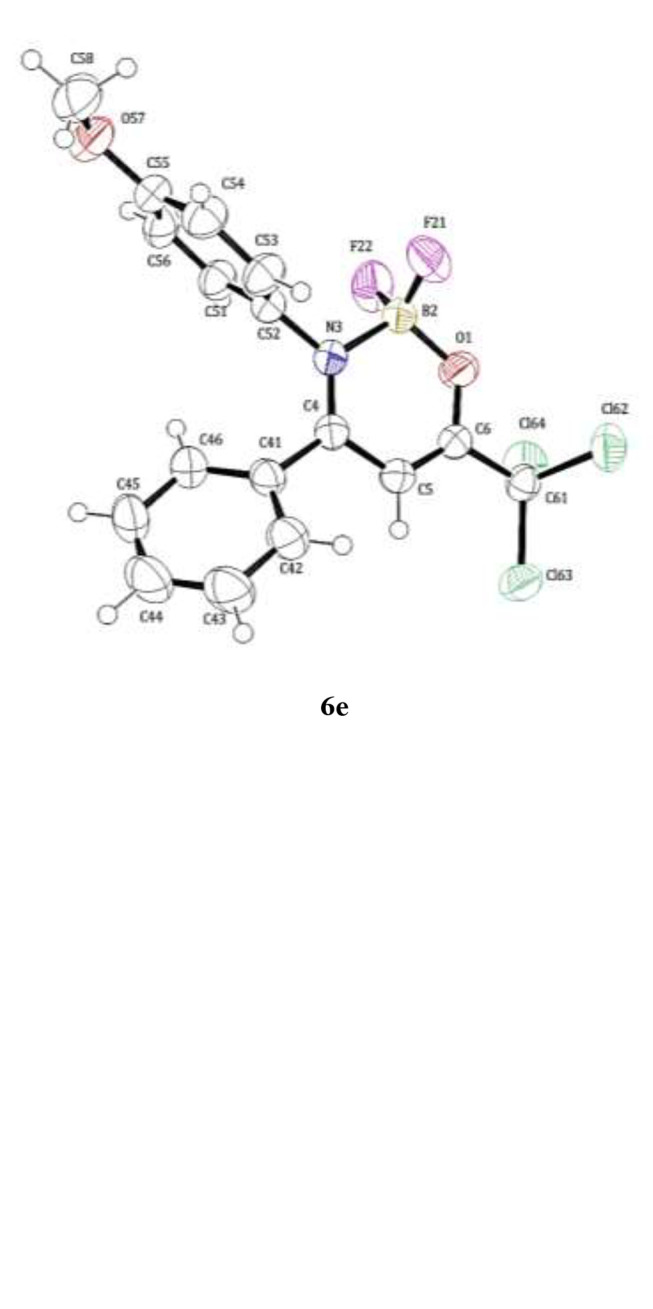
ORTEP of the crystal structure of (*Z*)-1,1,1-trichloro-4-((4-fluorophenyl)amino)-4-phenylbut-3-en-2-one (**4g**) (CCDC 1810806) and ORTEP of the crystal structure of 2,2-difluoro-3-(4-methoxyphenyl)-4-phenyl-6-(trichloromethyl)-2*H*-1,3,2-oxazaborinin-3-ium-2-uide (**6e**) (CCDC 1855153). Displacement ellipsoids are drawn at the 50% probability level and the H atoms are represented by circles with arbitrary radii.

The molecular structure of the compounds of series **4a-i**, **5e**, **6a-i**, and **7e** was determined and confirmed by single-crystal X-ray diffraction of the representative β-enaminone 4g and the boron complex **6e** (**Figure 1**). For the compound **6e** was observed that crystallization occurred at the monoclinic solvates-free form (**Figure 1**). The boron-atom has a tetrahedral geometry where the B-F, B-N, and B-O distances are 1.372, 1.590, and 1.452 Å, respectively and the bond angles around the B-atom are the 108.7° (F11-B1-N9) to 111.0° (F11-B1-F12). As observed, the O-B bond length is significantly shorter than the N-B one. The complete data of the X-ray diffraction are found in the **Supplementary Information File**.

### Absorption and Emission Properties of Boron Complexes

**Figure 2:**
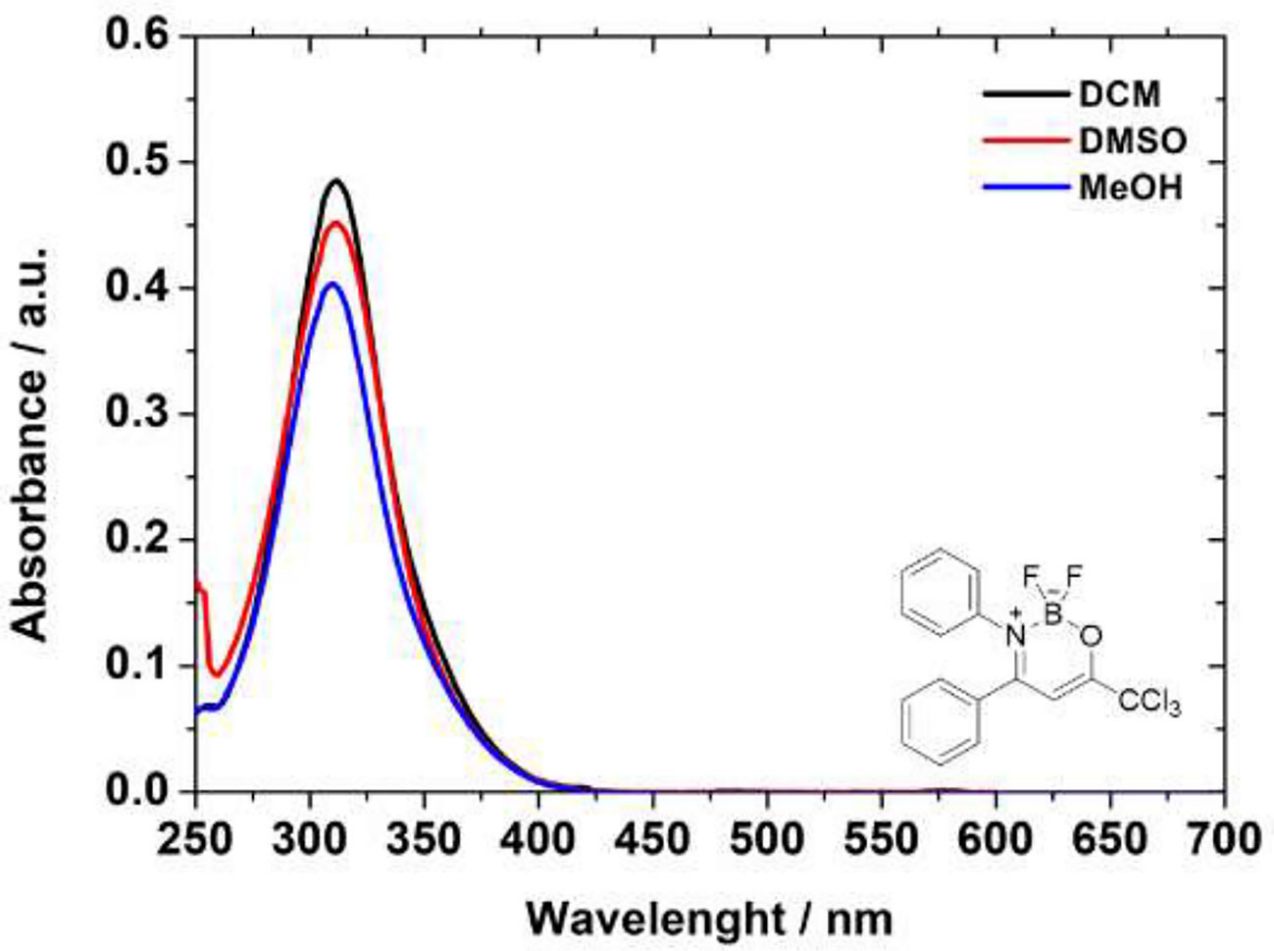
Comparative absorption spectra in solution of the BF_2_-complex compound **6c** in DCM (black solid line), DMSO (red solid line), and MeOH (blue solid line), respectively.

The UV-Vis analyses of the compounds use dichloromethane, dimethyl sulfoxide, and methanol as solvents are showed in the **Figure 2**. All absorption spectra are listed in the **Supplementary Information File** (**Figures S35**–**37**). The UV-Vis data are presented in **Table 2**, where the solvents are presented according to polarity parameters (Moyon and Mitra, 2011).

**Table 2:** UV-Vis absorption data and solvent parameters.

Comp.	Solvent	Δ*f* (ϵ, *n*)^a^	Abs (ϵ; M^-1^ cm^-1^)^b^
**6a**	DCM	0.22	301 (43,250)
	DMSO	0.26	300 (27,583)
	MeOH	0.31	299 (31,333)
**6b**	DCM	0.22	302 (41,416)
	DMSO	0.26	301 (36,000)
	MeOH	0.31	299 (28,500)
**6c**	DCM	0.22	311 (40,416)
	DMSO	0.26	311 (37,666)
	MeOH	0.31	310 (33,583)
**6d**	DCM	0.22	310 (39,500)
	DMSO	0.26	311 (32,833)
	MeOH	0.31	309 (28,000)
**6e**	DCM	0.22	309 (43,750), 372 (12,000)
	DMSO	0.26	309 (23,333), 375 (5,916)
	MeOH	0.31	307 (28,666), 370 (8,500)
**6f**	DCM	0.22	320 (43,083)
	DMSO	0.26	318 (27,166)
	MeOH	0.31	315 (19,416), 378 (28,333)
**6g**	DCM	0.22	312 (45,583)
	DMSO	0.26	311 (29,666)
	MeOH	0.31	310 (32,666)
**6h**	DCM	0.22	314 (34,500)
	DMSO	0.26	314 (30,166)
	MeOH	0.31	313 (29,583)
**6i**	DCM	0.22	309 (38,000), 380 (3,500)
	DMSO	0.26	307 (33,666)
	MeOH	0.31	308 (34,916), 373 (3,666)
**7e**	DCM	0.22	326 (43,333)
	DMSO	0.26	325 (35,833)
	MeOH	0.31	324 (31,416)

Solvents: DCM, Dichlorometahne; DMSO, Dimethylsulfoxide; MeOH, Methanol.

In general, all BF_2_-derivatives have absorption bands at UV nm region, with transitions according to the structure of these heterocycles. From the relatively large absorption coefficient (**Table 2**) it is plausible that this transition is S_1_(π) → S_0_(π) in nature. Yoshii et al. also reported and attributed the same nature transition in their compounds, although the authors measured in THF solution (Yoshii et al., 2013). Additionally, slightly solvatochromic behavior indicates no dependence of solvent properties. Unfortunately, all the BF_2_-derivatives studied here did not have luminescent properties in organic solution (Yoshii et al., 2013), regardless of the solvent used in the experiments (**Supplementary Information File**—**Figure S38**).

### TD-DFT—Theoretical Calculations

For a better insight into the frontier orbitals and the observed spectroscopic properties of compounds **6a-i** and **7e**, TD-DFT (Time-Dependent Density-Functional Theory) theoretical calculations were performed for compounds of series **6a-i** and 7e using the Gaussian 09 package of programs (Frisch et al., 2016). All geometrical structures were optimized at the SCRF(PCM)-B3LYP/cc-pVTZ level of theory. The values calculate for the maximum absorption wavelengths were closed and in agreement with the experimental results in DCM, DMSO, and MeOH (solvent effect). As example, **Table 3** highlights the electron distribution of the HOMO and LUMO for compound **6c**. It is found that the HOMO and LUMO densities in **6c** were delocalized over the whole molecule. The same behavior could be observed for all compounds studied (**Supplementary Information File**—**Figures S58**–**S88**).

**Table 3:** Molecular orbital amplitude plots (generated with 0.02 au isovalue), excitation energy (E), and oscillator strengths (*f*) for HOMO-LUMO orbitals, calculated at the TD-DFT (SCRF(PCM))-B3LYP/cc-pVTZ level in DCM, DMSO, and MeOH for 2,2-difluoro-3,4-diphenyl-6-(trichloromethyl)-2*H*-1,3,2-oxazaborinin-3-ium-2-uide (**6c**).

Solvent	HOMO	E/λ/*f*	LUMO
DCM	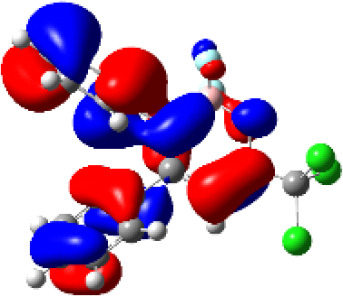	3.4565 eV 358.70 nm 0.1148	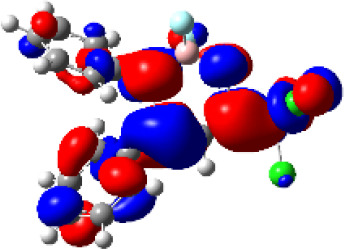
DMSO	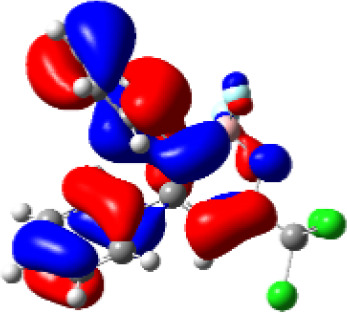	3.4611 eV 358.22 nm 0.1023	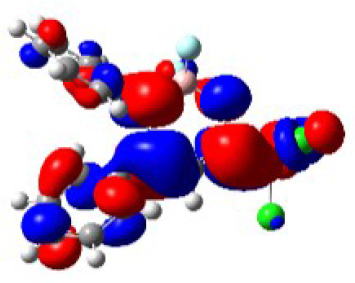
MeOH	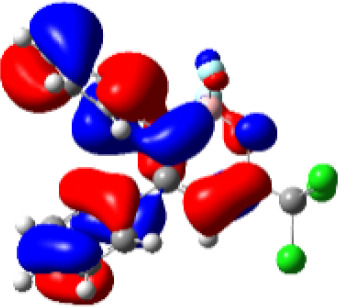	3.4639 eV 357.93 nm 0.0995	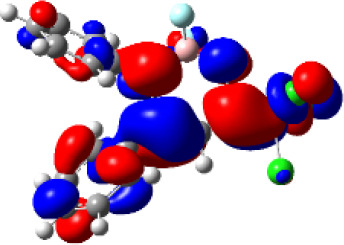

Solvents: DCM, Dichlorometahne; DMSO, Dimethylsulfoxide; MeOH, Methanol.

### CT-DNA Binding Experiments by Absorption UV-Vis Analysis

To investigate the mentioned interactions, UV-Vis and emission fluorescence analysis are preferred because small molecule-DNA interactions may be experimentally monitored by changes in the intensity and position of the spectroscopic peak responses or changes in the dynamic viscosity of DNA (Chen et al., 1999; Ni et al., 2006).

**Figure 3:**
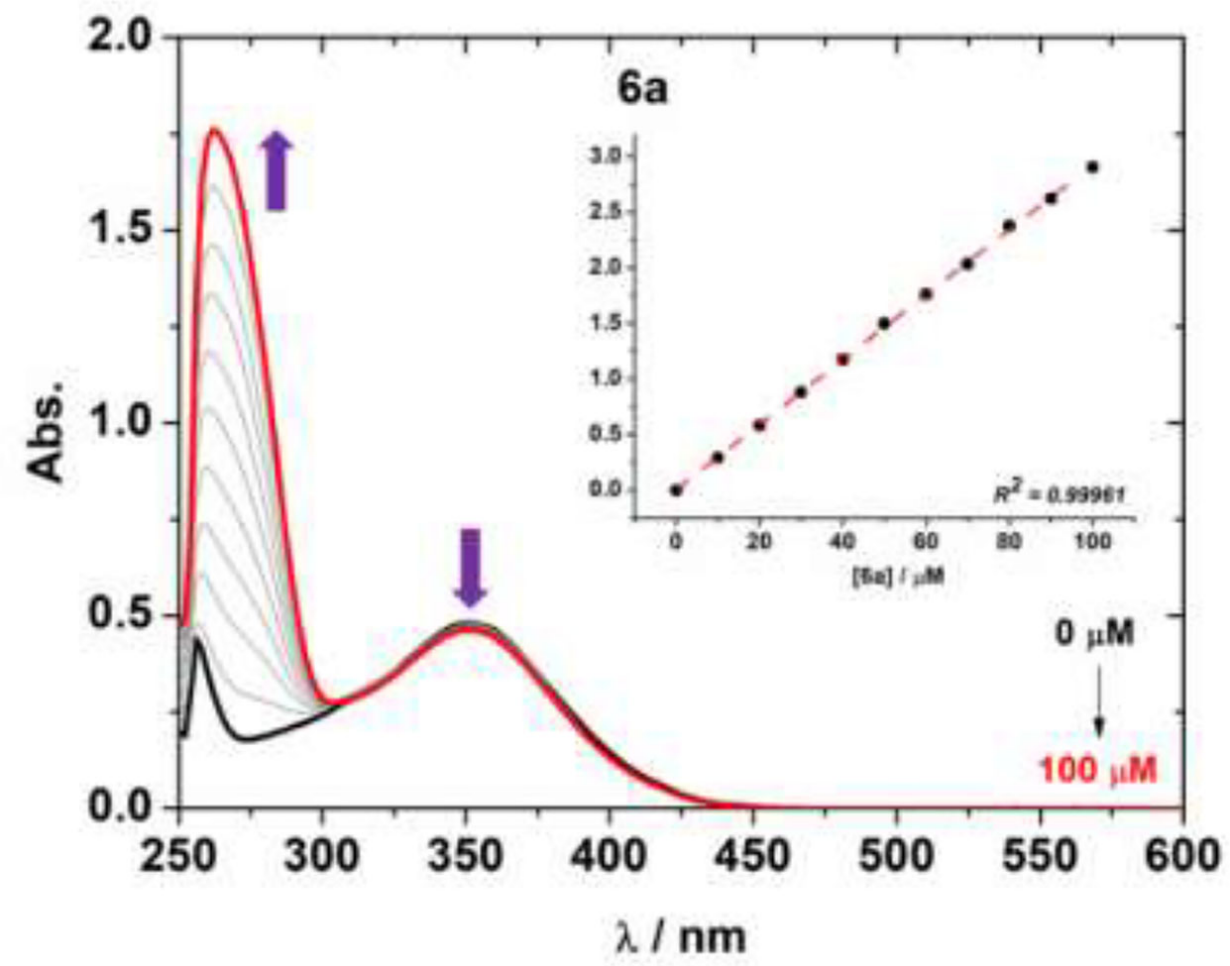
UV-Vis absorption spectra for compound **6a** and the effect of successive additions of CT-DNA solution in the presence of a fixed concentration of **6a**, in a DMSO (2%)/Tris-HCl pH 7.2 buffer mixture. Insert graph shows the plot [DNA]/(ϵa – ϵf) *versus* [DNA]. The concentration of CT-DNA ranged from 0 to 100 μM.

In the present study, the interaction of the boron complexes **6a-i** and **7e** against CT-DNA (Calf Thymus DNA) was studied by UV-Vis at 250–600 nm region using DMSO (2%)/Tris-HCl pH 7.2 buffer mixture solution. The effect of different concentrations of CT-DNA on the UV-Vis spectra of derivative **6a** is presented in **Figure 3**. Other BF_2_-derivatives absorption spectra are shown in **Figures S39**–**S47** in the **Supplementary Information File** and the binding parameters are listed in **Table 4**.

**Table 4:** CT-DNA-binding data from UV-Vis absorption and fluorescence emission of boron complexes.

	CT-DNA by absorption				EB-DNA by emission		
Comp.	*H*(%)^a^	Δλ(nm)^b^	K_b_(M^-1^)^c^	ΔG°(kcal/mol)^d^	*Q*(%)^e^	K_SV_(M^-1^)^f^	*k*_q_(M^-1^ s^-1^)^g^
**6a**	3.55	0.0	1.67 × 10^5^	−7.12	7.08	7.47 × 10^2^	3.24 × 10^10^
**6b**	3.68	0.0	1.72 × 10^5^	− 7.13	3.45	3.44 × 10^2^	1.49 × 10^10^
**6c**	6.26	3.0	2.44 × 10^5^	− 7.34	10.95	1.17 × 10^3^	5.09 × 10^10^
**6d**	4.07	0.0	1.85 × 10^5^	− 7.17	4.85	5.07 × 10^2^	2.20 × 10^10^
**6e**	4.74	0.0	1.98 × 10^5^	− 7.22	5.86	6.72 × 10^2^	2.92 × 10^10^
**6f**	3.10	6.0	1.51 × 10^5^	− 7.06	5.43	3.26 × 10^2^	1.42 × 10^10^
**6g**	2.11	6.0	1.17 × 10^5^	− 6.90	14.53	1.64 × 10^3^	7.13 × 10^10^
**6h**	4.85	0.0	2.23 × 10^5^	− 7.28	7.02	6.98 × 10^2^	3.03 × 10^10^
**6i**	3.68	0.0	1.76 × 10^5^	− 7.14	11.34	1.20 × 10^3^	5.22 × 10^10^
**7e**	7.06	0.0	4.76 × 10^5^	− 7.73	7.31	9.05 × 10^2^	3.93 × 10^10^

a H(%) = (Abs_initial_ − Abs_final_)/(Abs_initial_) × 100 at 300–400 nm range;

b Δλ (nm) = λ _final_- λ _initial_;

c Binding constant by UV-Vis CT-DNA analysis;

d Gas constant R = 1.9858775 kcal K^-1^ mol^-1^ and temperature T = 298K;

e Q(%) = (Emission_initial_ − Emission_final_)/(Emission_initial_) × 100;

f Stern-Volmer quenching EB-DNA constant (K_SV_) by steady-state emission spectra;

g Stern-Volmer rate quenching EB-DNA constant (k_q_) by steady-state emission spectra.

Generally, the absorption spectra of organoboron compounds can be changed at 300–400 nm range upon several additions of CT-DNA (hypochromicity properties). A slight bathochromic shift in some cases was also observed, indicating a weak or non-electrostatic interaction observed between the cited compounds and CT-DNA (**Table 4**). The transition change of the derivatives may be a result of the interaction of the BF_2_ moiety with the nucleobase residues of the DNA (**Figure 3**), which are possible *via* hydrophobic forces, as previously in the literature (Bonacorso et al., 2018b).

Furthermore, the binding constant (K_b_) data for the BF_2_-compounds were listed in **Table 4**. These K_b_ values are associated to the BF_2_-DNA complex stability, while the free energy indicates the spontaneity/non-spontaneity of derivative-DNA binding process, indicating the spontaneity of the interaction between the organoboron complexes and DNA.

### Competitive Experiments With DNA by Steady-State Emission Fluorescence

**Figure 4:**
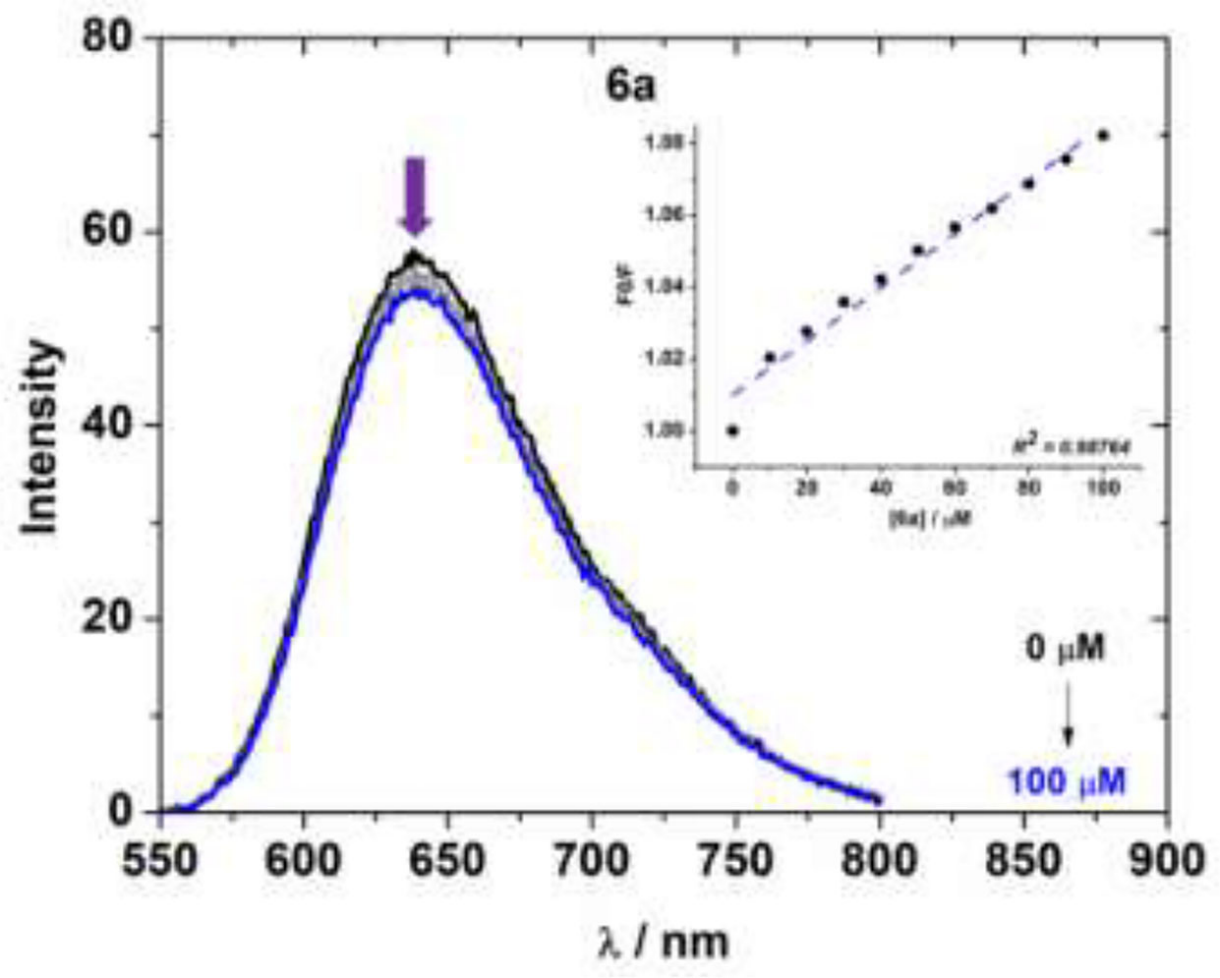
Fluorescence emission spectra of EB bound to CT-DNA in the presence of **6a** in a DMSO (2%)/Tris-HCl pH 7.2 mixture at λ_exc_ = 510 nm. The inset shows the plot of F_0_/F versus the concentration of compound 6a according to the Stern-Volmer equation.

In the emission assays, EB-DNA (Ethidium Bromide-DNA) analysis were also conducted to determine the displacement of the intercalating agent ethidium bromide (EB) dye from CT-DNA. As example, the fluorescence emission of compound 6a was monitored by increasing the compound concentration at a fixed concentration of CT-DNA pre-treated with EB dye (**Figure 4**). The EB-DNA adduct emission fluorescence spectra of other derivatives are listed in **Figures S48**–**S56** in the **Supplementary Information File**.

The EB-DNA competition experiment shows an intense emission band located at λ_em_ = 649 nm by excitation at λ_exc_ = 510 nm. After adding derivative **6a** to the EB-DNA solution, the EB-DNA emission appeared to decrease in emission intensity. This fact demonstrates a weak fluorescence quenching of the EB-DNA adduct, as estimated by the K_SV_ values (**Table 4**). This behavior can be assigned to the competition of the BF_2_ complexes with EB over binding to the base pair of CT-DNA (intercalation mode). High values were obtained for the quenching constant rate (*k*q), which were higher than the diffusion rate constant (*k*_diff_ ≈ 7.40 × 10^9^ L mol^-1^ s^-1^ at 298K) (Montalti et al., 2006). These observed values indicate a possible static interaction mechanism between the BF_2_-complexes and EB-DNA (Montalti et al., 2006), which takes place *via* ground state association.

### Antimicrobial Activity

Twenty of the newly synthesized compounds **4a-i**, **5e**, **6a-i**, and **7e** were evaluated for them *in vitro* antimicrobial activity against a panel of microorganisms including yeasts, filamentous fungi, bacteria, and alga by determining their MIC and minimal fungicidal/bactericidal/algacidal concentrations using broth microdilution methods according to CLSI standard protocols (CLINICAL AND LABORATORY STANDARDS INSTITUTE (CLSI), 2008; CLINICAL AND LABORATORY STANDARDS INSTITUTE (CLSI), 2012; CLINICAL AND LABORATORY STANDARDS INSTITUTE (CLSI), 2015).

In order to classify the antimicrobial activity, antibacterial and two antifungal agents currently employed in therapeutics were compared (**Supplementary Information File**—**Table S13**). Therefore, the range of MICs until 10 μg/ml was considered significant activity for yeast-like fungi, with MIC-ranges between 20 and 40 μg/ml considered moderate activity and concentrations beyond not being considered. For filamentous fungi and *P. zopfii*, the MICs until 1.0 μg/ml were considered strong activity, with MIC ranges from >1 to 4 μg/ml considered moderate activity and above this range not being considered. For bacteria, all MICs under 4.0 μg/ml were considered as active and above they were not considered (**Supplementary Information File**—**Table S13**). The comparisons among MICs and MCC (minimal “cidal” concentrations) revealed that they were similar in 66% of cases (48/73) and showed that the MCC were higher by one or more concentration in 34% (25/73). The comparisons are important because they show the differences between compounds that are only inhibitory from those able to inhibit and kill pathogenic microorganisms.

Considering the antimicrobial activity of the synthesized compounds, the algaecidal action against *P. zopfii* stands out. Compounds **4a**, **4b**, **4d**, **4e**, **6c**, **6e**, **6f**, and **6h** were strongly effective in both inhibiting growth and causing algae death at a concentration below 1.0 µg/ml, while the other compounds shows moderate activity against this alga (except **4i** and **5e**). These results are encouraging, since *P. zopfii* is an environmental agent of bovine mastitis that causes many losses resulting from the compromised quality and production of milk and, in addition, it presents high antimicrobial resistance and the optimal treatment strategy for this infection has not yet been well established. In addition, *P. zopfii* can cause cutaneous and serious systemic infections in humans (Lass-Flörl and Mayr, 2007). The compounds that showed higher inhibitory and algaecidal activity were **4d**, **4e**, **6e**, and **6f** (MIC = 0.31 µg/ml). These results showed that changes in the basic structure as well as in the substituent slightly increased activity against *P. zopfii*.

In relation to the evaluated pathogenic yeasts (*Candida* spp. and *Cryptococcus gattii*) only compound **6i** showed moderate activity for both, the other compounds of series **6** (**6b-h**) were moderately effective in inhibiting only the growth of *C. gattii*, as well as compounds **4a**, **4b**, **5e**, and **7e**. Compounds **4a-i** and 5e have the same substituents as compounds **6a-i** and **7e** although the basic structure is comprised only by a β-enaminoketone for the former, whereas for compounds **6a-i** and **7e** a basic structure is constituted by a boron complex. Compounds **6a-j** and **7e** showed moderate activity against *C. neoformans*, and remained inactive against *S. cerevisiae*. Considering the difficulty in treating meningitis caused by *C. gattii*, which is normally resistant to one of the main antifungal drugs used in this pathology, fluconazole, increasing the mortality rate, potential treatment alternatives are extremely necessary.

Among filamentous fungi selected to study the activity of β-enaminoketone boron complex, the four most frequent agents of the life-threatening disease aspergillosis (*A. fumigatus*, *A. flavus*, *A. niger*, and *A. terreus*) were chosen. It is important to note that the antifungal therapy for these microorganisms is difficult and therapeutic failures are frequent (Leimann et al., 2004). Immunocompromised and neutropenic patients require fungicidal agents to treat their infections due to immunologic system failures (Dignani et al., 2003; Nucchi et al., 2004; Walsh et al., 2004).

Significant results were observed for compounds **4c**, **4g**, and **4h**, which exhibited the best activities against *Aspergillus*. *A. niger* growth was completely inhibited at the concentration of 2.5 µg/ml. For boron complexes (**6a-i** and **7e**), concentrations two or more times higher were required to inhibit *A. niger* growth. Compound 4g showed moderate activity against *A. flavus* (MIC = 20 µg/ml), which is the second most important agent of aspergillosis.

Another interesting point is the lack of activity of **4i** against all the filamentous fungi studied when compared with **6i**. The incorporation of BF_2_ resulted in the acquisition of antifungal activity. This shows that substituents in the boron complexes can bring significant differences in antimicrobial activities.

The activity of the series of compounds against a panel of bacteria clinically important was poor, with the best activities observed with **6c** (MIC = 5.0 µg/ml) and 6i (MIC = 5.0 µg/ml), which were both against *K. pneumoniae*, and an opportunistic gram-negative rod. This is curious because, in general, the gram-positive cocci, which is represented here by *S. aureus*, is more sensible than gram negative rods (Kiska and Gilligan, 1999; Pfaller and Diekema, 2004).

The cytotoxicity of the β-enaminoketones and boron complexes were assessed using *in vitro* cell-based assay with 3T3 fibroblasts as the cell model, and MTT as endpoints to determine cell viability. As shown in **Table S13** (**Supplementary Information File**), all compounds tested at concentrations between 1 and 100 µg/ml exhibited low or negligible cellular toxicity, as determined by MTT assay.

## Conclusion

In summary, the aim of this study was to evaluate the synthetic potential of β-methoxyvinyl trichloromethyl ketones for the synthesis of trichloromethyl substituted β-enaminoketones (**4a-i**, **5e**) (61–90%), which may be used to obtain a new series of 2,2-difluoro-3-alkyl(aryl)-4-phenyl-6-(trichloromethyl)-2*H*-1,3,2-oxazaborinin-3-ium-2-uides (**6a-i**, **7e**) in moderate to very good yields (50–91%). Unfortunately, it was not possible to obtain the trifluoromethyl-substituted analogs.

The results of the DNA-binding studies by spectroscopic analysis of the new BF_2_-β-enaminoketone compounds indicated that the greatest interaction with nucleic acids.

Multidrug-resistant microorganisms are increasingly common. These types of microorganisms can resist the effects of conventional antimicrobial drugs, increasing the mortality rates in both humans and animals affected by infections that are difficult to treat. In this context, new and more potent compounds are required. The series presented here showed to be able to inhibit the growth of several tested microorganisms, with outstanding algaecidal activity, however weak or no antibacterial activity, and moderate antifungal activity. We believe that these compounds could be chemically modified to improve their antimicrobial activity. Some compounds from the present series exhibited potent antimicrobial effects on various pathogenic microorganisms at concentrations below those that showed cytotoxic effects. Notably **4d**, **4e**, **6e**, and **6f** showed the best results and were very significant against P. *zopfii*, which is an agent that causes diseases in humans and animals.

Finally, the new molecules presented here open good prospects for the development of analogue structures with possible application in microbiology and studies involving interactions with biomolecules. The introduction of other known chromophore substituents in similar chelates to those reported here is under initial development and will be published in due course.

## Data Availability Statement

The raw data supporting the conclusions of this article will be made available by the authors, without undue reservation, to any qualified researcher.

## Author Contributions

WR, IR, MR, NZ, and HB: These researchers and students were responsible for the development of the part concerning the synthesis, purification, NMR, GC-MS and X-ray diffractometric data, and TD-DFT calculations for the new compounds. HC, LD, and PL: These researches were responsible for the development of the part related to the evaluation of the minimum inhibitory concentration (MIC) of the series of compounds **5–7**. Twenty of the newly synthesized compounds **4a-i**, **5e**, **6a-i**, and **7e** were evaluated for their *in vitro* antimicrobial activity against a panel of microorganisms including yeasts, filamentous fungi, bacteria, and alga by determining their MIC and minimal fungicidal/bactericidal/algaecidal concentrations using broth microdilution methods according to CLSI standard protocols. TA and BI: This researcher and the student were responsible for the development of the part concerning to the study of the optical properties of the new compounds (UV-vis, fluorescence, quantum yield calculations, Stokes shift) and the interaction of the boron complexes **6a-i** and **7e** against CT-DNA, which was studied by UV–Vis absorption spectroscopy.

## Conflict of Interest

The authors declare that the research was conducted in the absence of any commercial or financial relationships that could be construed as a potential conflict of interest.
