# Supplementary material for: Novel Alkyl(aryl)-Substituted 2,2-Difluoro-6-(trichloromethyl)-2H-1,3,2-oxazaborinin-3-ium-2-uides: Synthesis, Antimicrobial Activity, and CT-DNA Binding Evaluations
Source: Front Pharmacol. 2020 Sep 2;11:1328. doi: 10.3389/fphar.2020.01328 (PMC7493717; doi:10.3389/fphar.2020.01328)
Supplement: Supplementary file 1 [file DataSheet_1.pdf]

Supporting Information

Novel Alkyl(aryl)-Substituted 2,2-Difluoro-6-(trichloromethyl)-2*H*-1,3,2-oxazaborinin-3-ium-2-uides: Synthesis, Antimicrobial Activity, and CT-DNA Binding Evaluations

Wilian C. Rosa,[a] Inaiá O. Rocha,[a] Melissa B. Rodrigues,[a] Helena S. Coelho,[b,c] Laura B. Denardin,[b] Pauline C. Ledur,[b] Nilo Zanatta,[a] Thiago V. Acunha,[d] Bernardo A. Iglesias[d*] and Helio G. Bonacorso[a*]

[a] Núcleo de Química de Heterociclos (NUQUIMHE), Departamento de Química, Universidade Federal de Santa Maria, 97.105-900, Santa Maria, RS, Brazil..

[b] Laboratório de Pesquisas Micológicas (LAPEMI), Departamento de Microbiologia e Parasitologia, Universidade Federal de Santa Maria, 97105-900, Santa Maria, RS, Brazil.

[c] Instituto Federal de Educação, Ciência e Tecnologia Farroupilha, Santa Maria, RS, Brazil.

[d] Laboratório de Bioinorgânica e Materiais Porfirínicos, Departamento de Química, Universidade Federal de Santa Maria, Santa Maria, 97105-900, RS, Brazil.

Table of Contents

1. **NMR spectra of compounds 4a-i, 5e, 6a-i and 7e………** S3
2. **HMRS spectra of compounds 4c, 4d, 4e and 4g………..** S33
3. **Single Crystal X-Ray Data of compounds 4g and 6e.....** S37
4. **UV-Vis spectra……………………………………………** S45
5. **Steady-state emission fluorescence spectra…………….** S47
6. **UV-Vis DNA titrations spectra………………………..…** S48
7. **Competitive EB-DNA assays by emission fluorescence spectra…..** S53
8. **TD-DFT Calculations for compounds 6a-i, 7e………..** S58
9. **Antimicrobial assays……………………………………** S89
10. **Cytotoxicity assays……………………………………...** S91

1. ^{1}H-, ^{13}C-, ^{11}B-, ^{19}F-NMR spectra of compounds 4a-i, 5e, 6a-i and 7e.

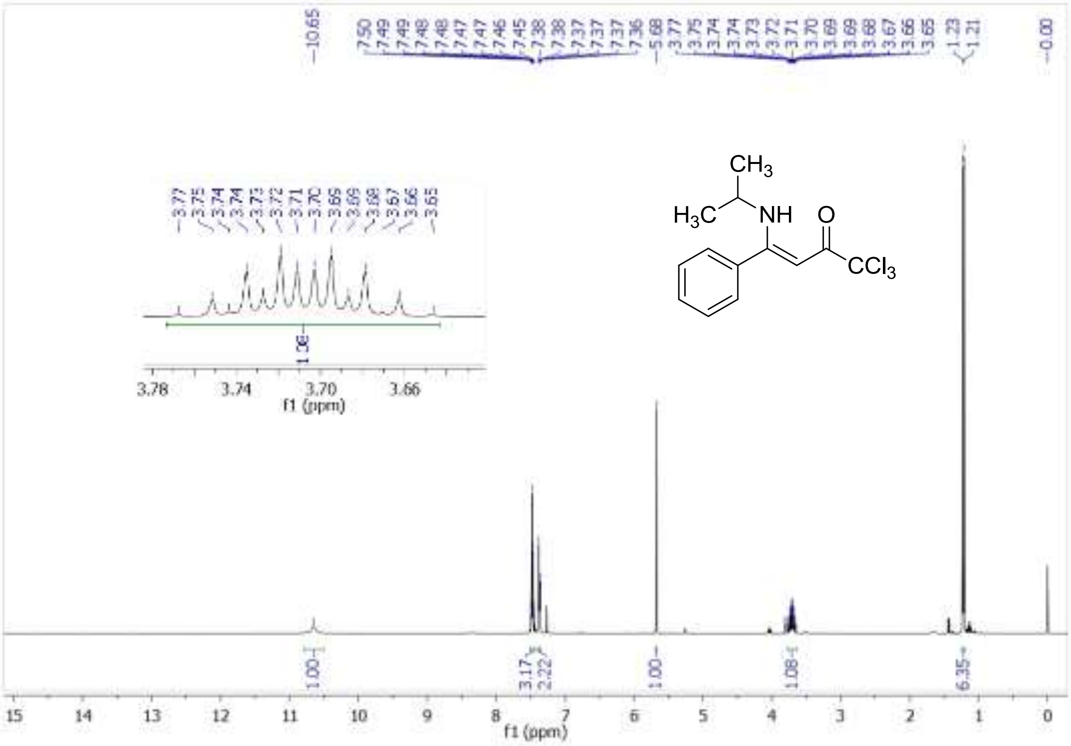

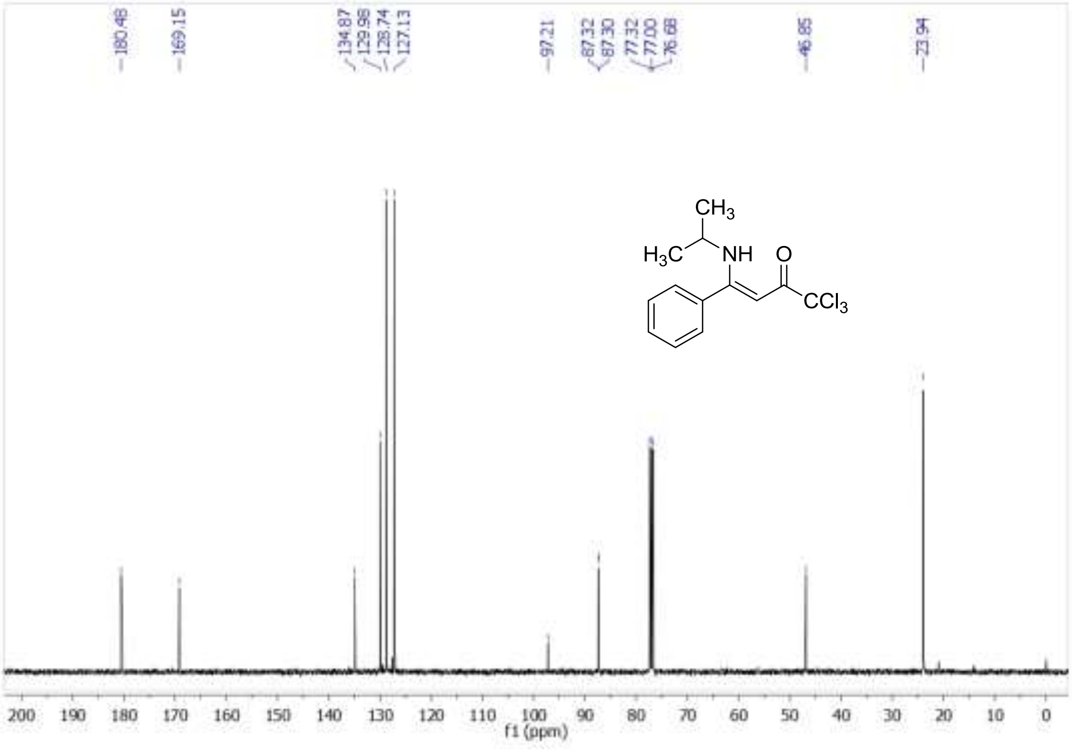

Figure S1. ^{1}H- and ^{13}C-NMR in $CDCl_3$ at 400 and 101 MHz, respectively of **c**ompound **4a**.

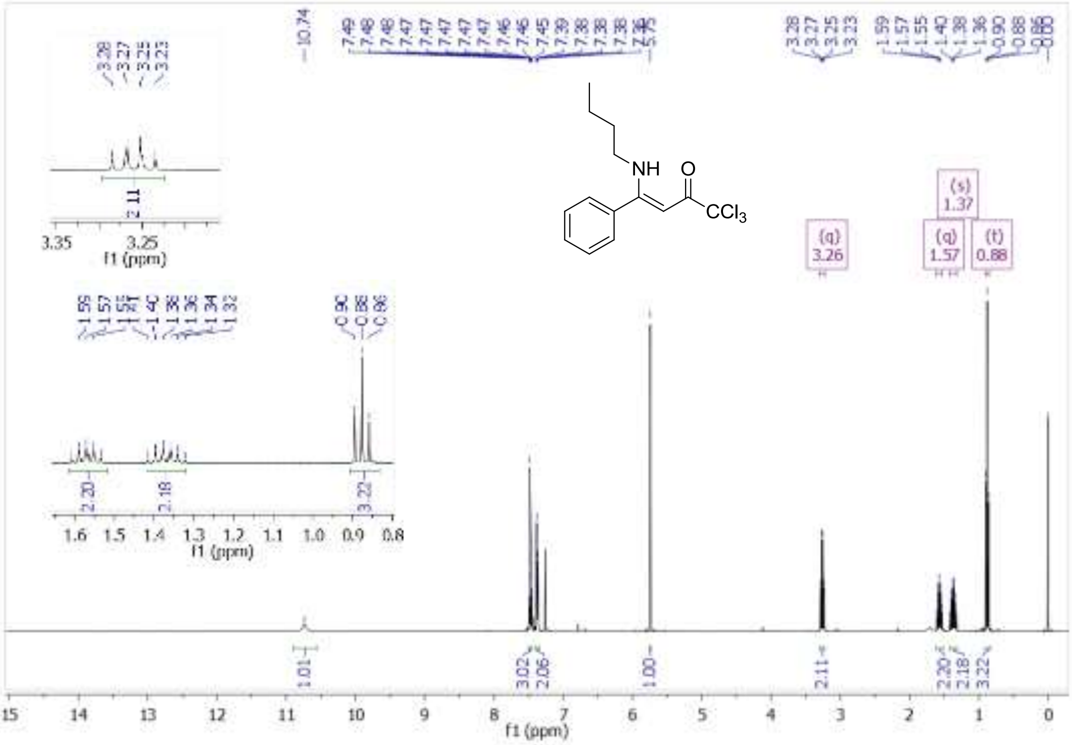

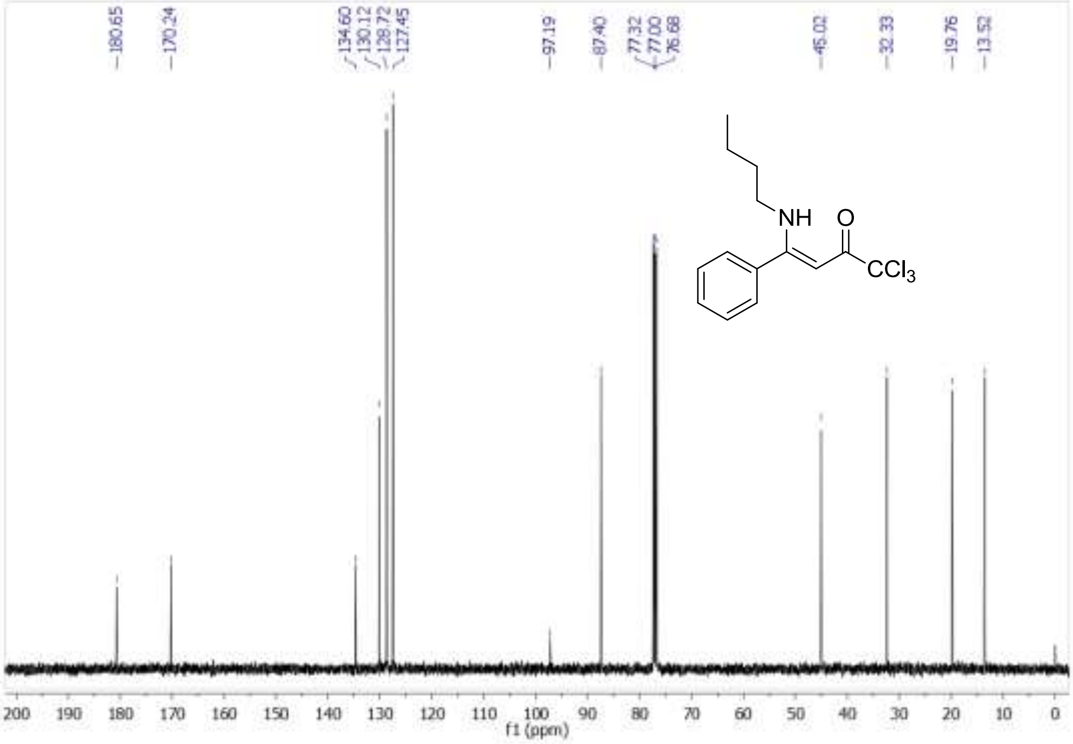

Figure S2. ^{1}H- and ^{13}C-NMR in $CDCl_3$ at 400 and 101 MHz, respectively of compound **4b**.

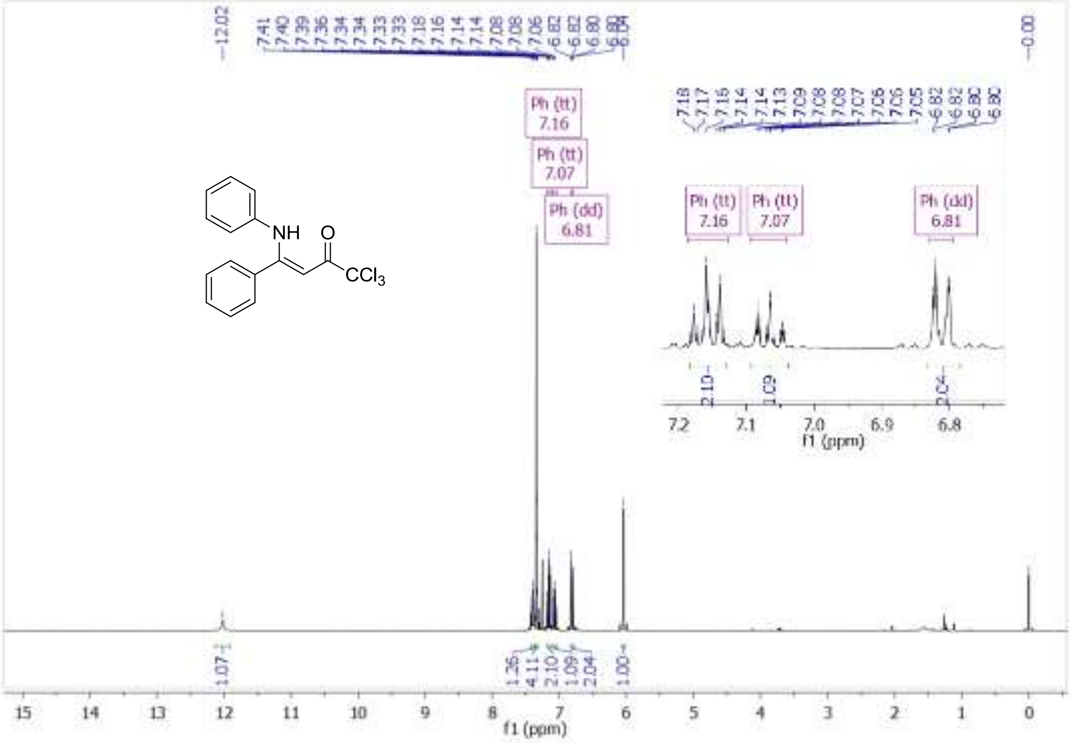

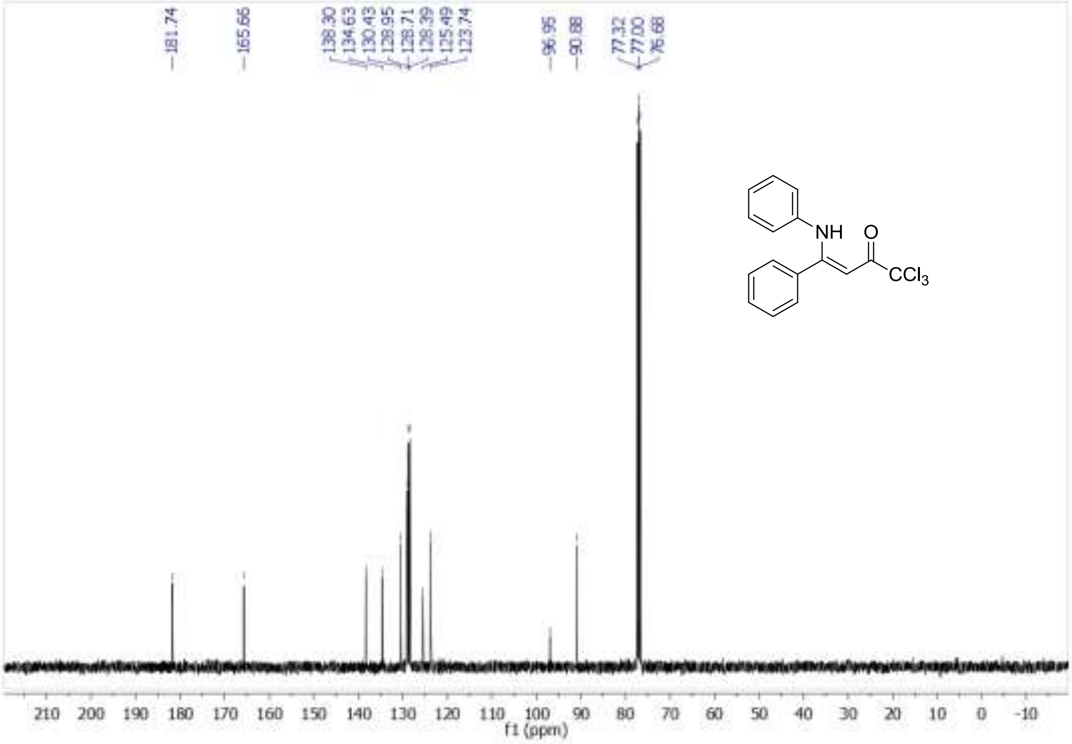

Figure S3. ^{1}H- and ^{13}C-NMR in $CDCl_3$ at 400 and 101 MHz respectively of compound **4c**.

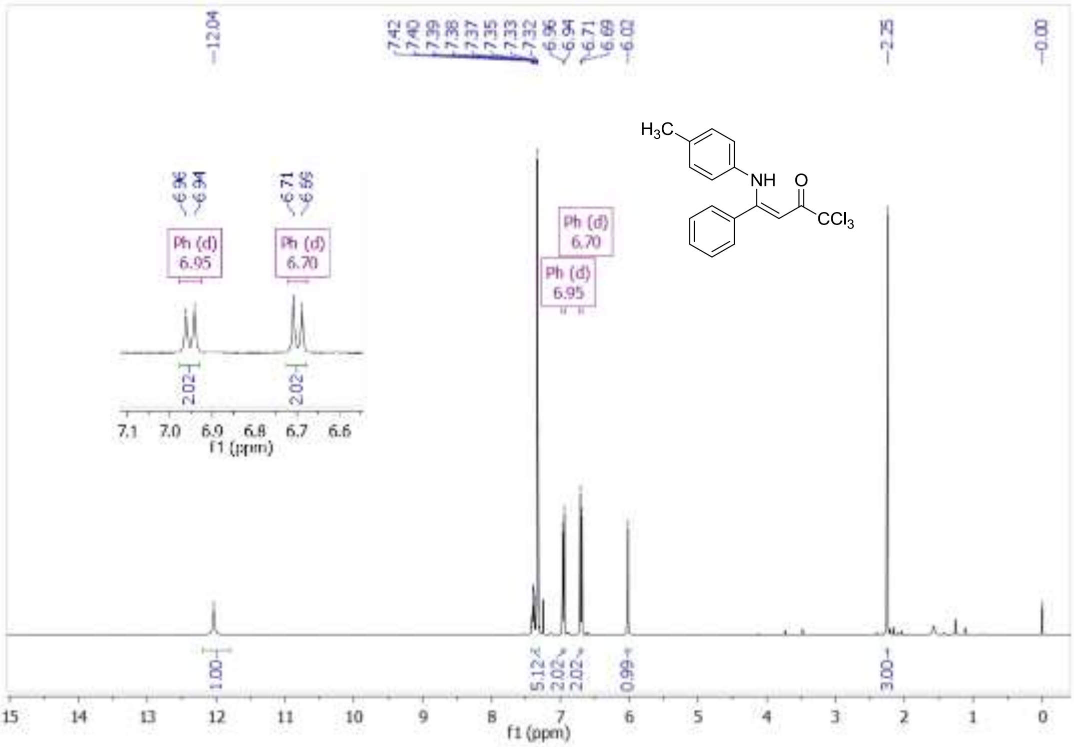

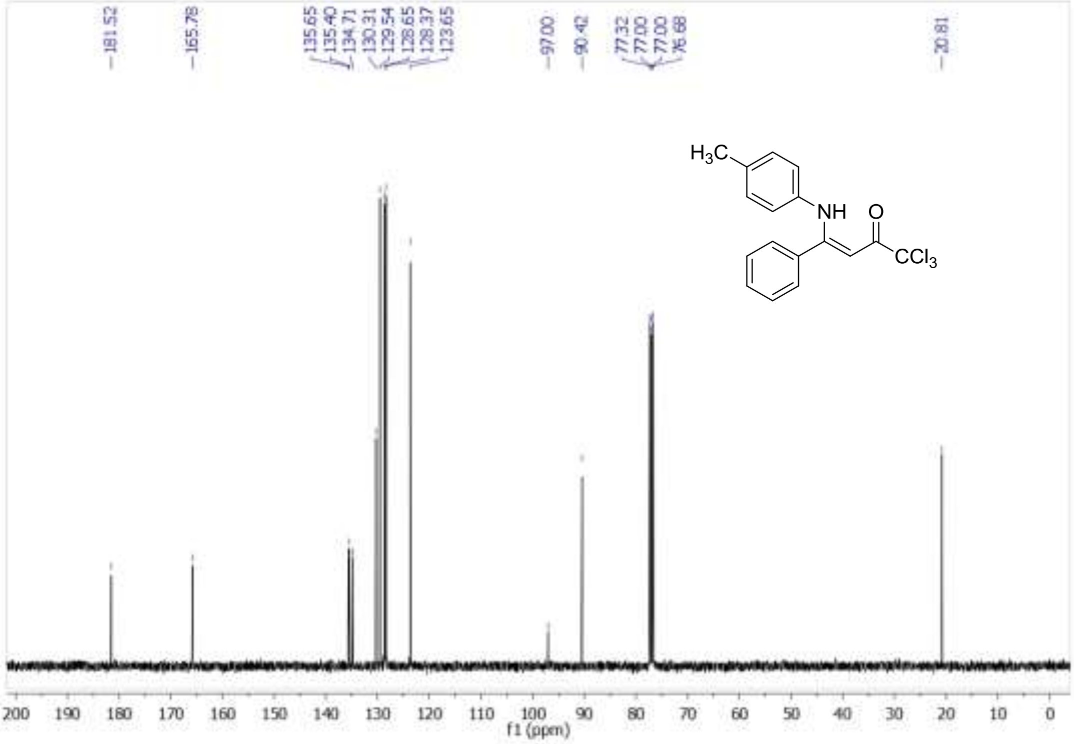

Figure S4. ^{1}H- and ^{13}C-NMR in $CDCl_3$ at 400 and 101 MHz, respectively of compound **4d**.

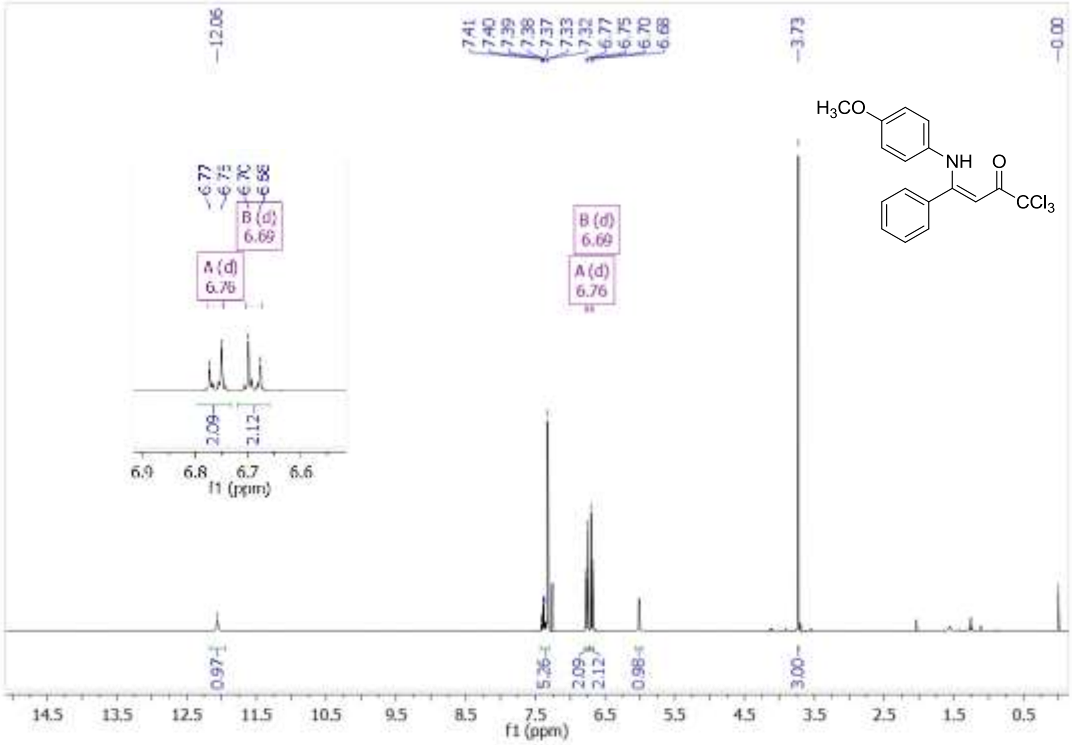

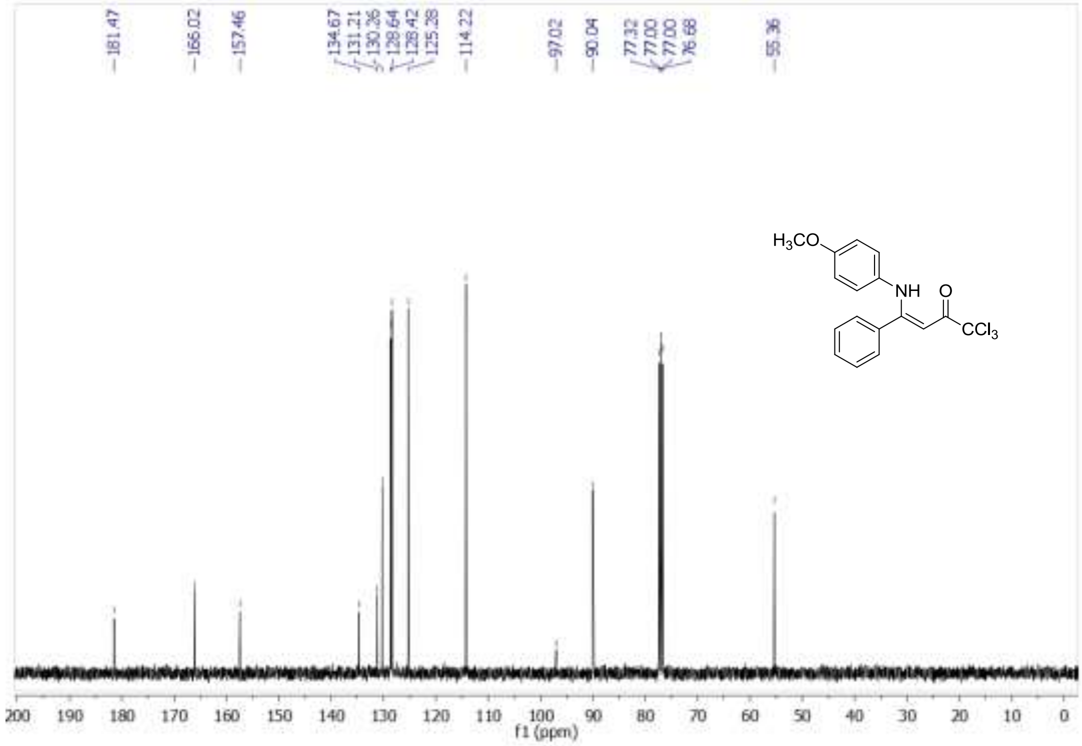

Figure S5. ^{1}H- and ^{13}C-NMR in $CDCl_3$ at 400 and 101 MHz, respectively of compound **4e**.

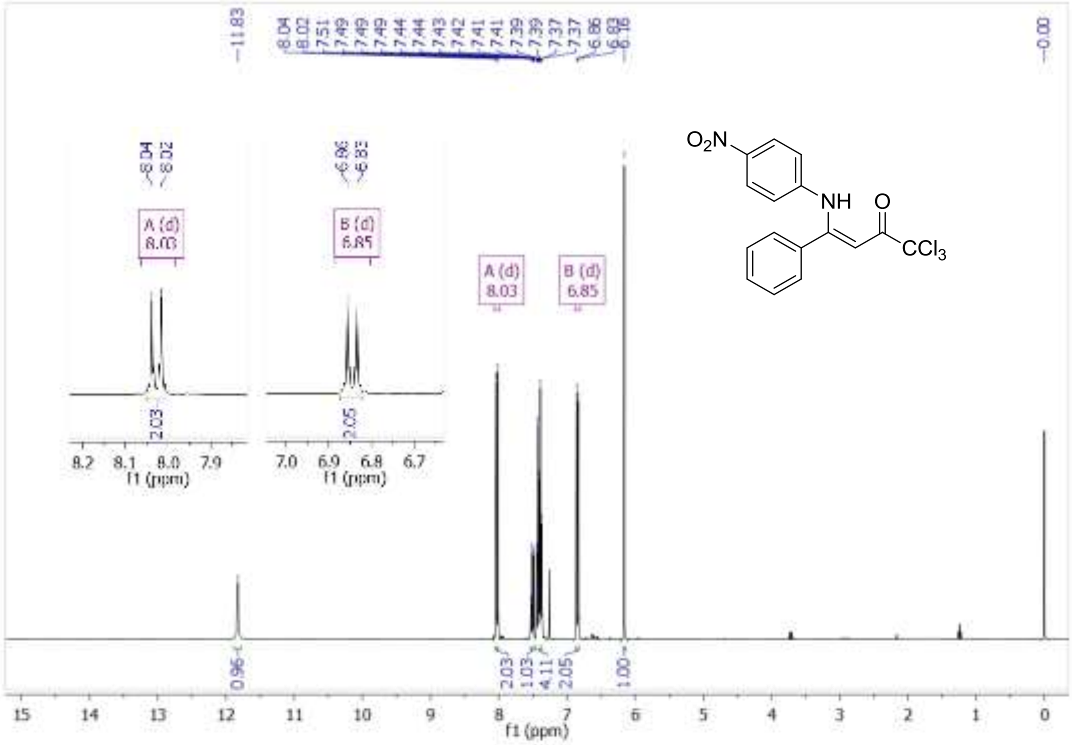

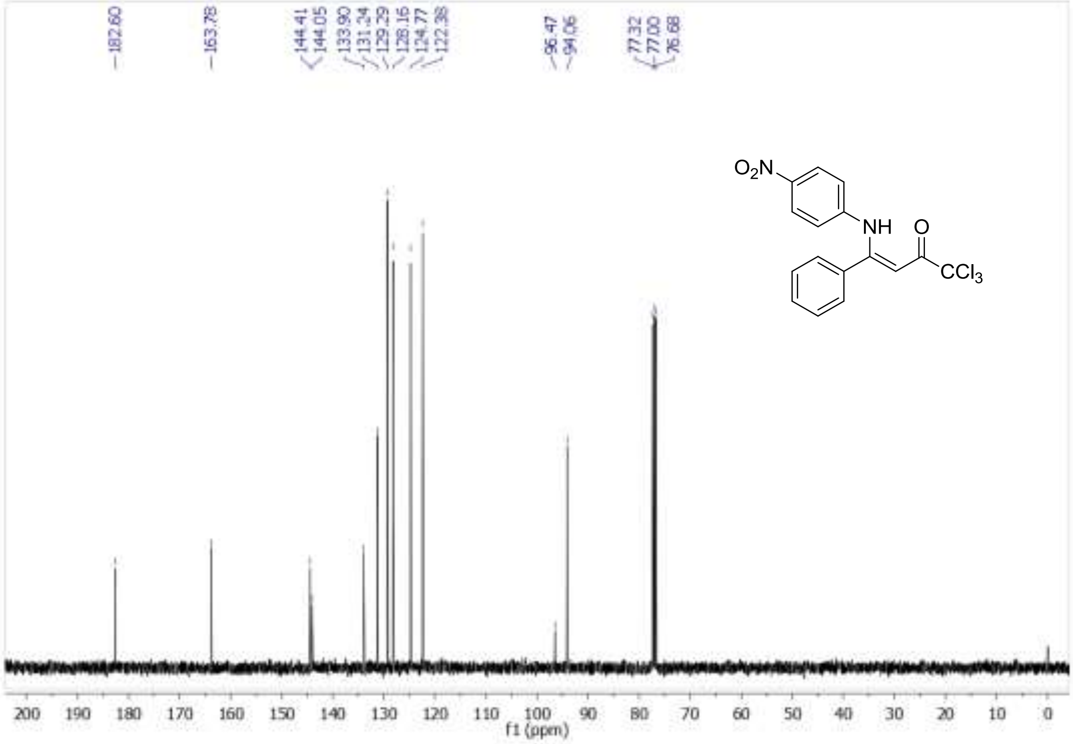

Figure S6. ^{1}H- and ^{13}C-NMR in $CDCl_3$ at 400 and 101 MHz, respectively of compound **4f**.

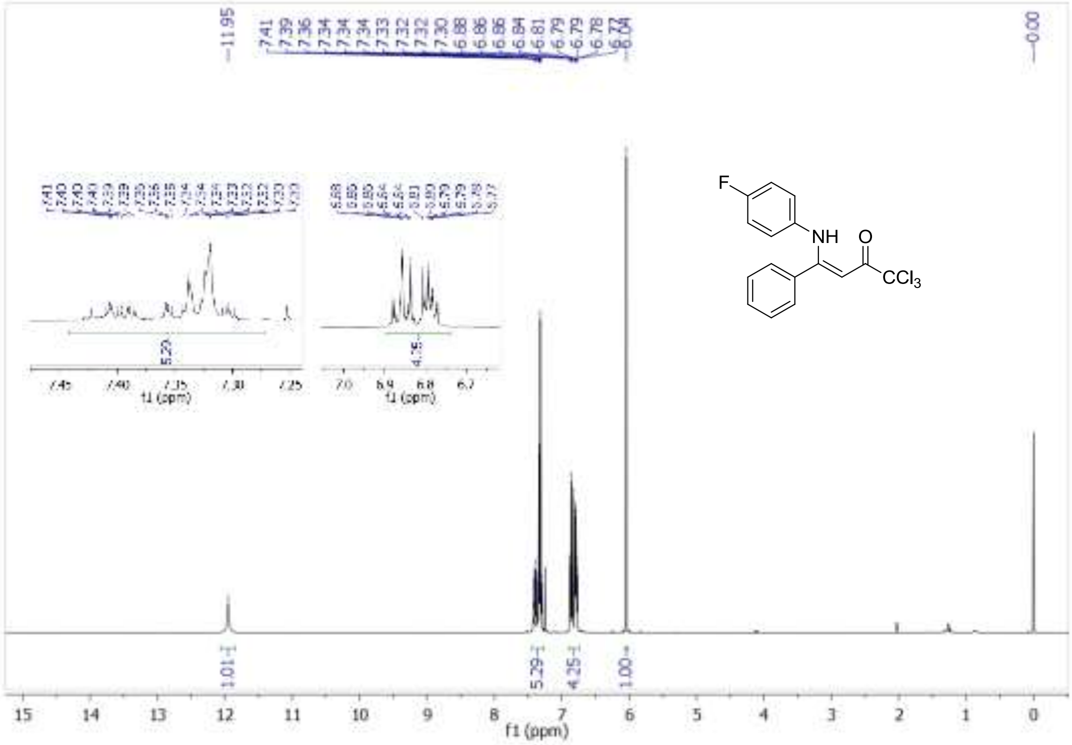

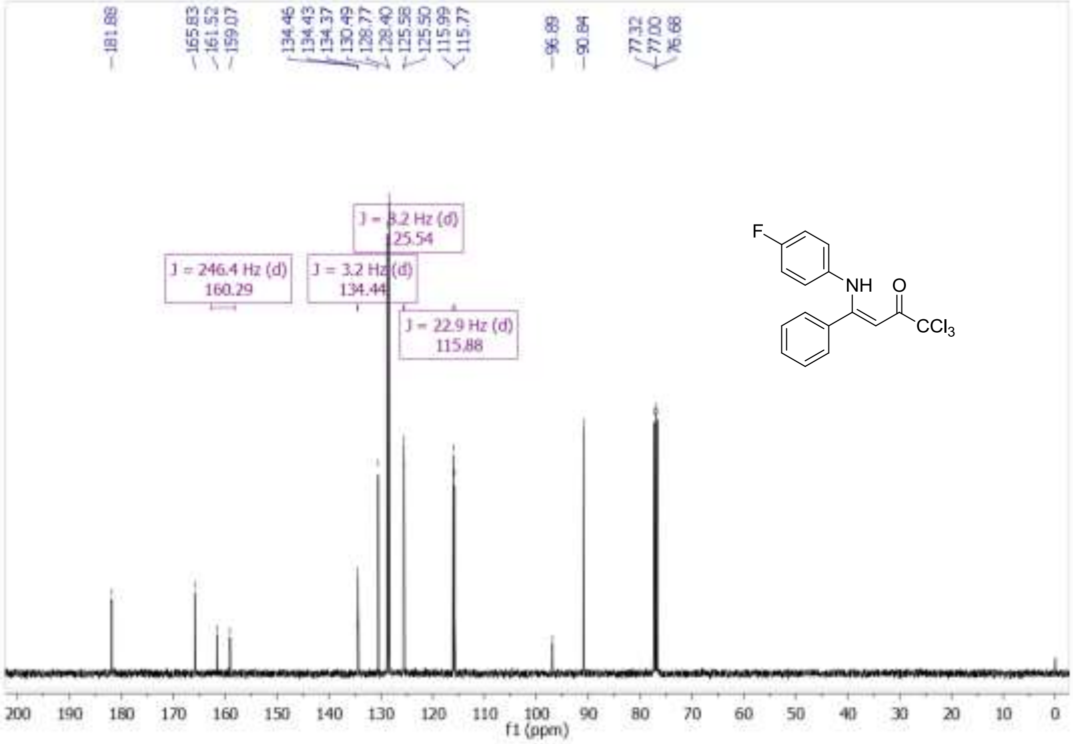

Figure S7. ^{1}H- and ^{13}C-NMR in $CDCl_3$ at 400 and 101 MHz, respectively of compound **4g**.

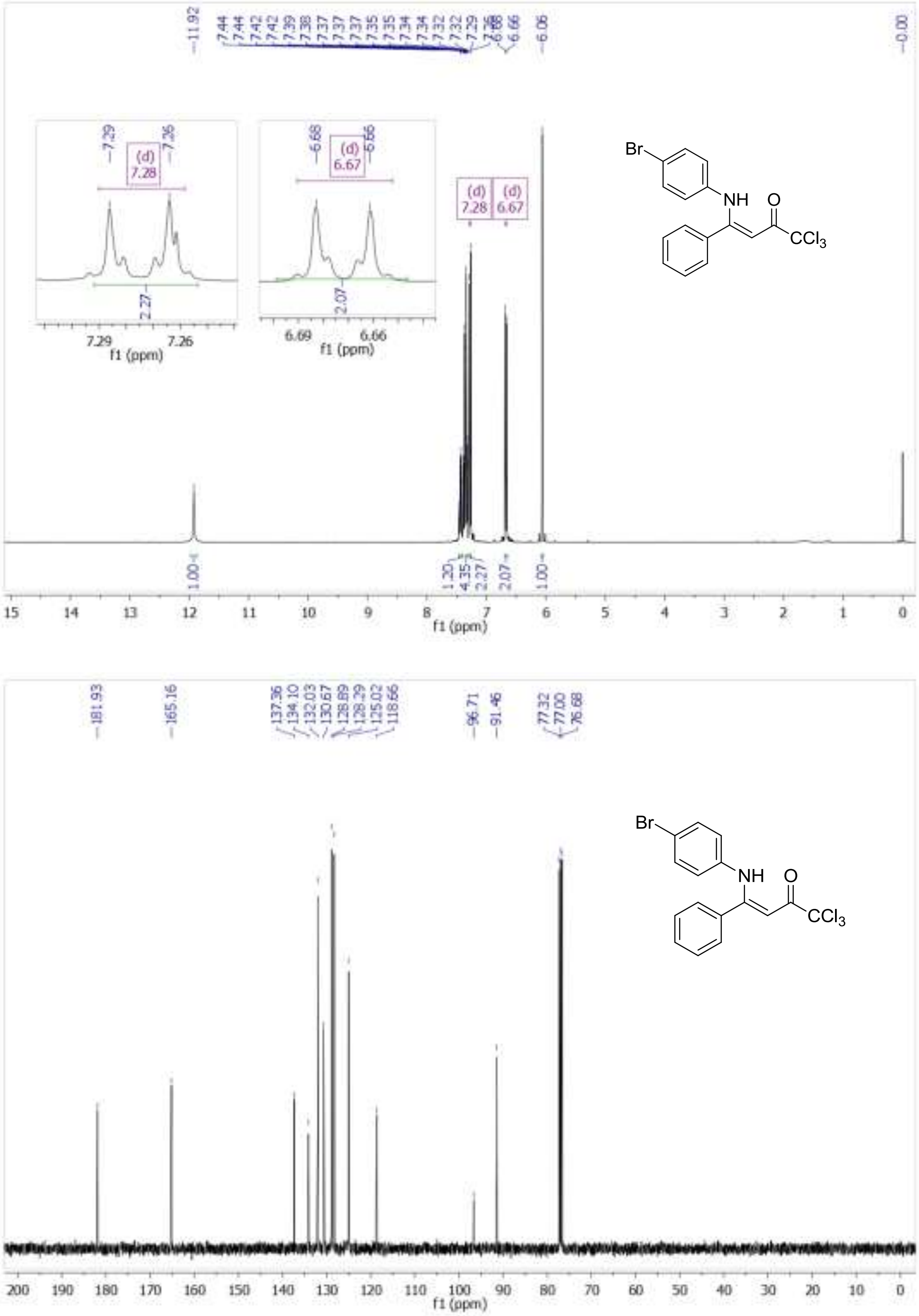

Figure S8. ^{1}H- and ^{13}C-NMR in $CDCl_3$ at 400 and 101 MHz, respectively of compound **4h**.

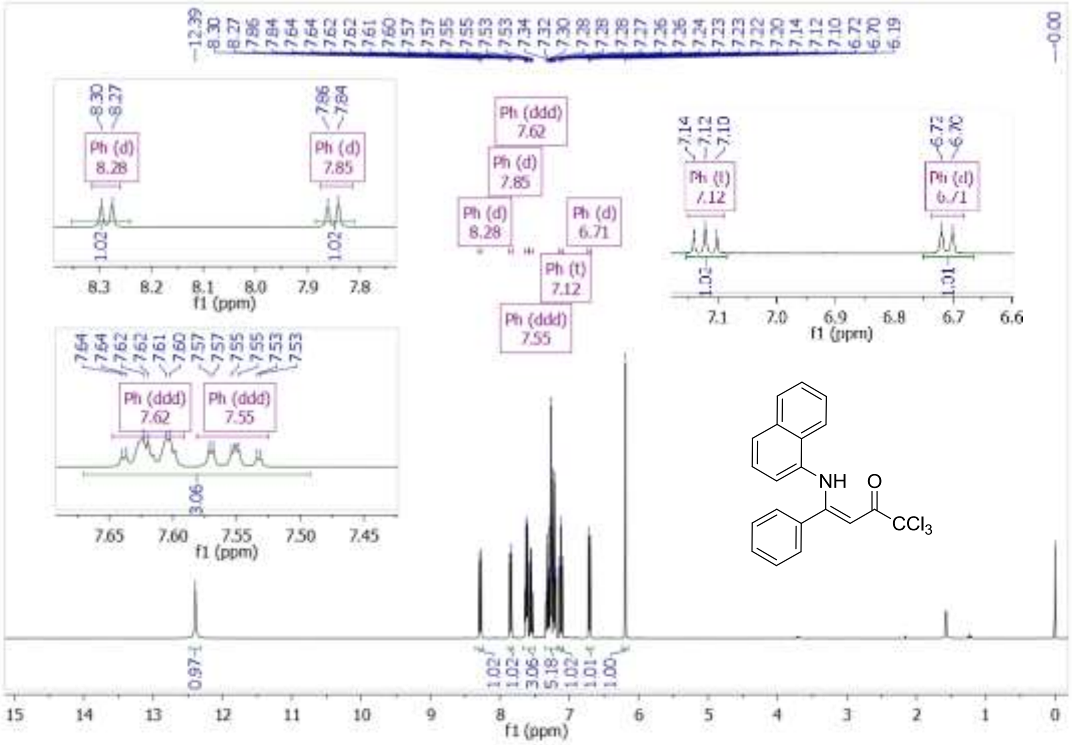

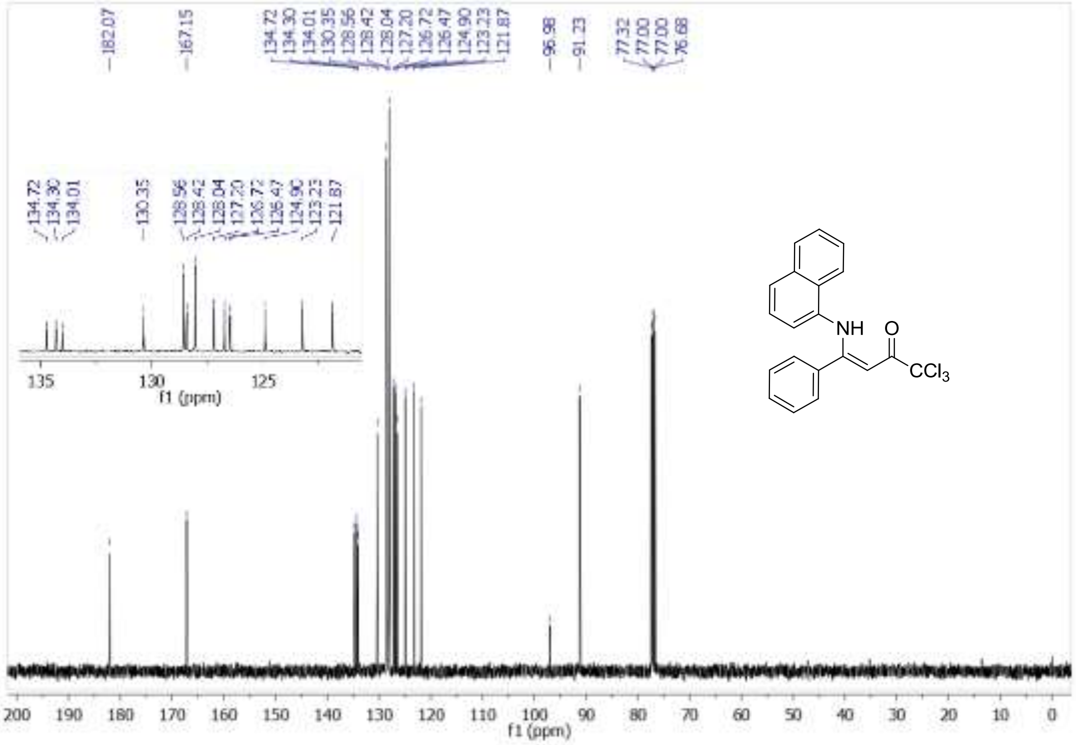

Figure S9. ^{1}H- and ^{13}C-NMR in $CDCl_3$ at 400 and 101 MHz, respectively of compound **4i**.

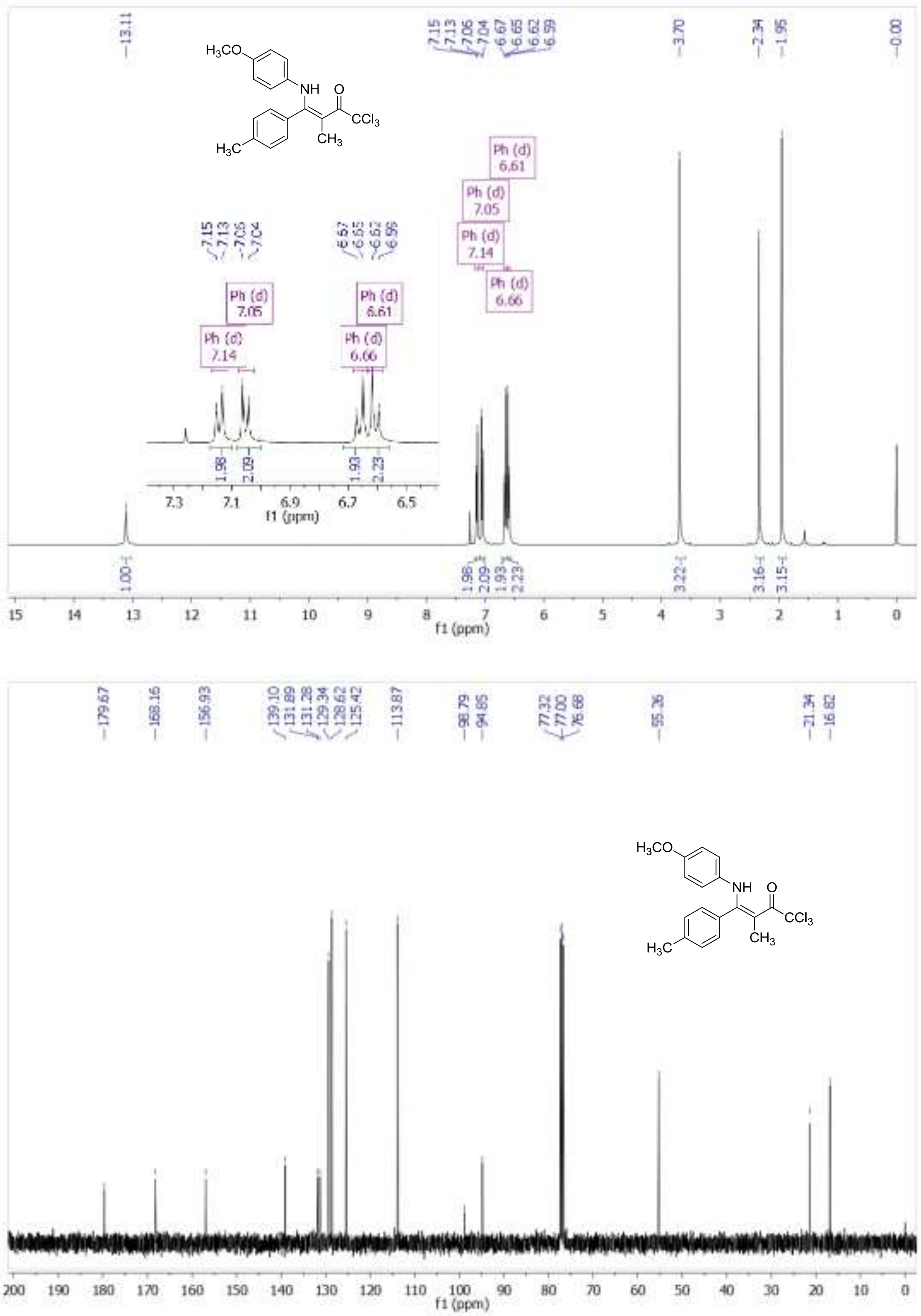

Figure S10. ^{1}H- and ^{13}C-NMR in $CDCl_3$ at 400 and 101 MHz, respectively of compound **5e**.

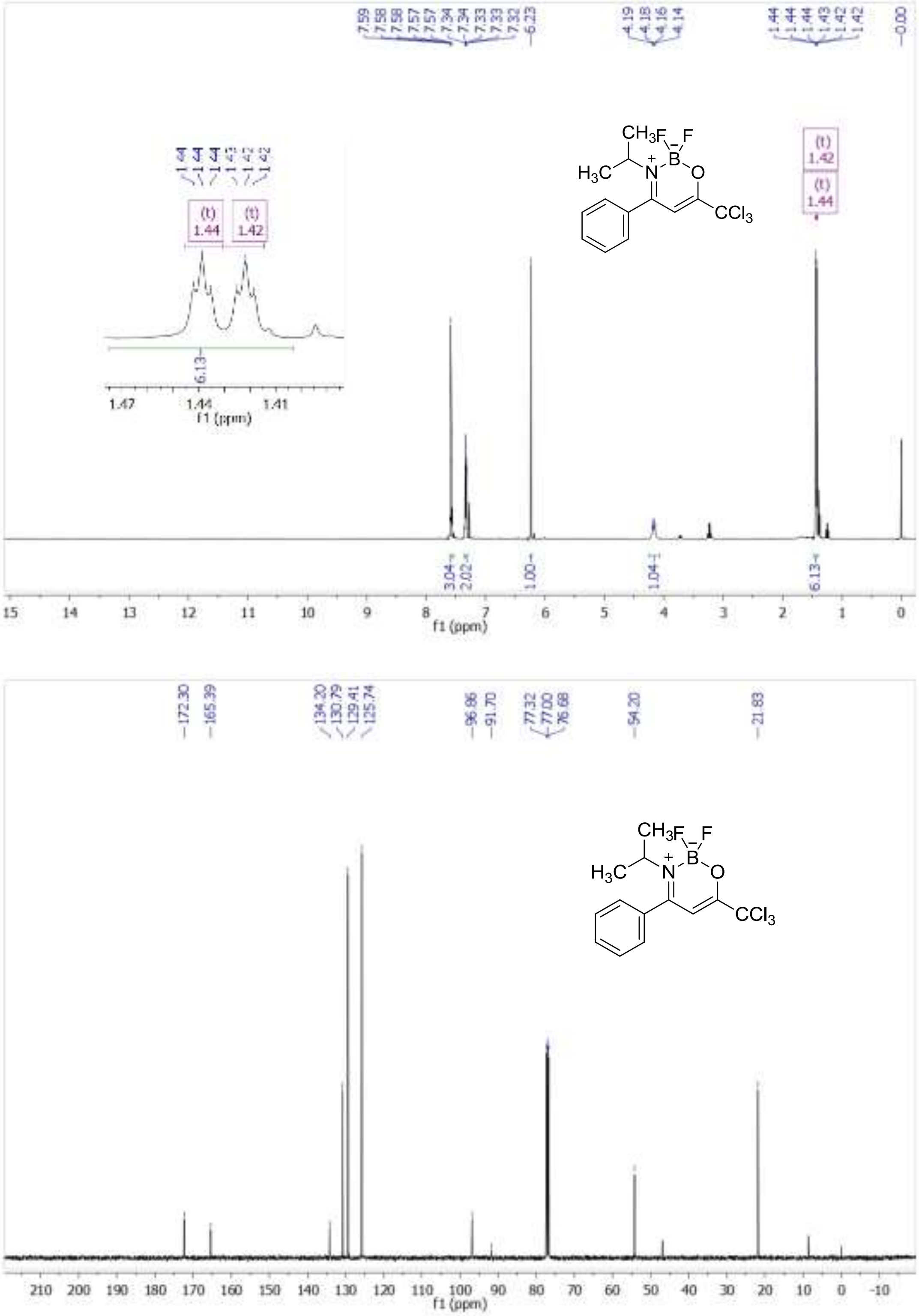

Figure S11. ^{1}H- and ^{13}C-NMR in $CDCl_3$ at 400 and 101 MHz, respectively of compound **6a**.

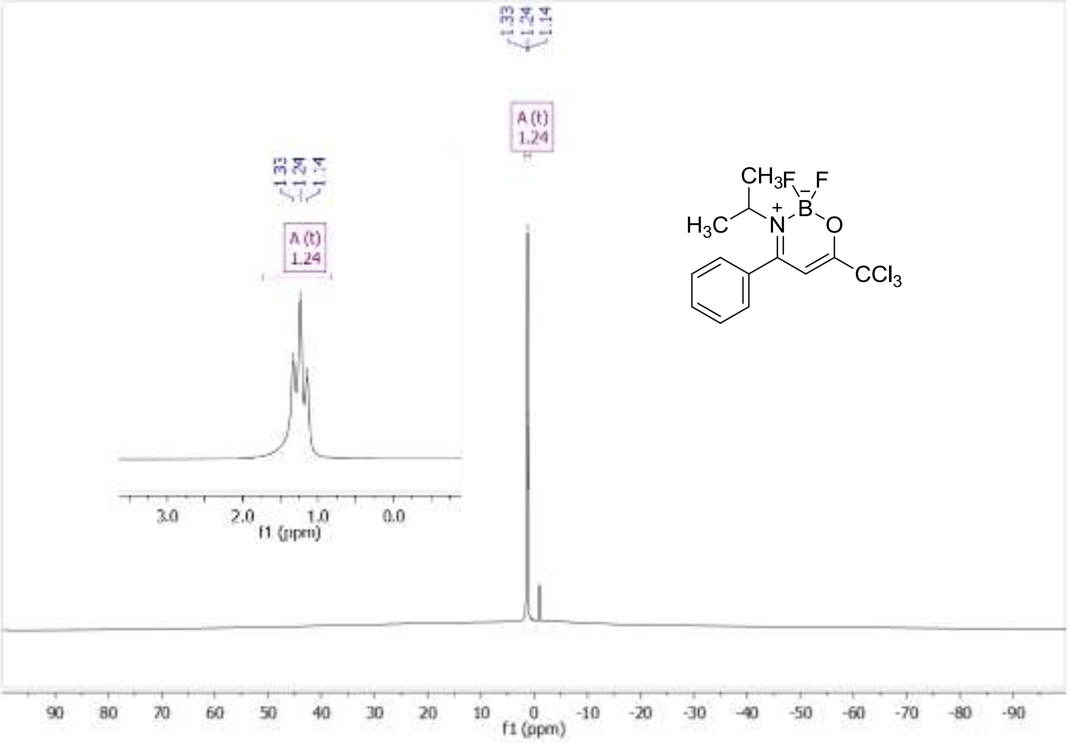

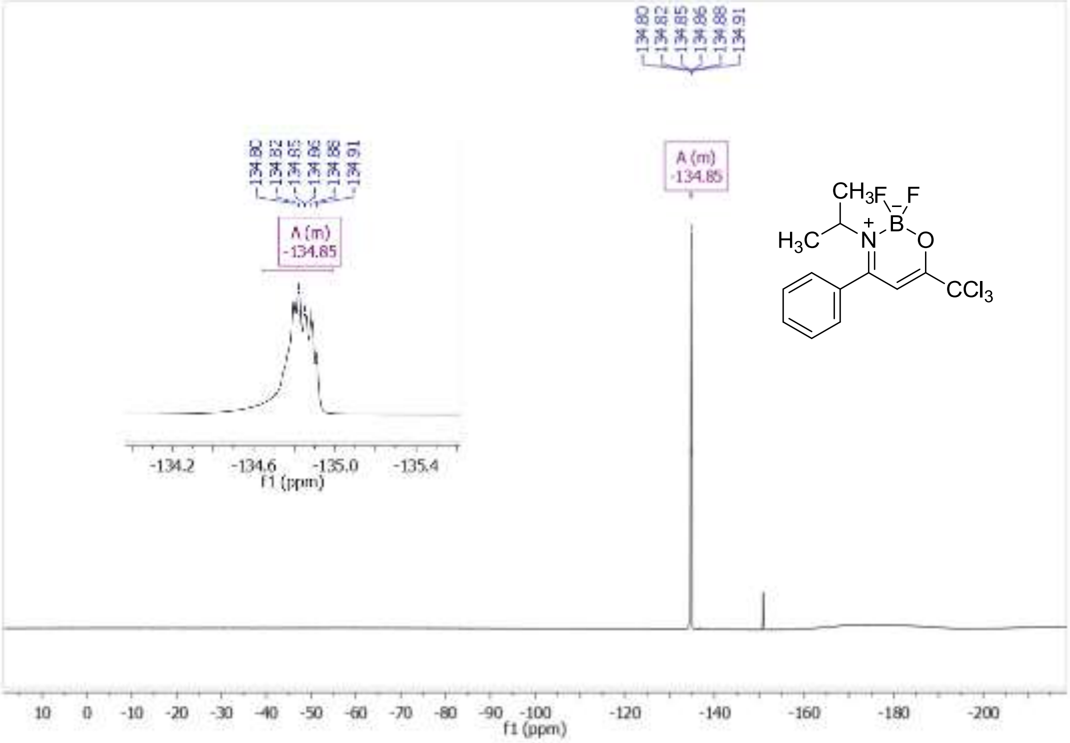

Figure S12. ^{11}B and ^{19}F-NMR in $CDCl_3$ at 193 and 565 MHz, respectively of compound **6a**.

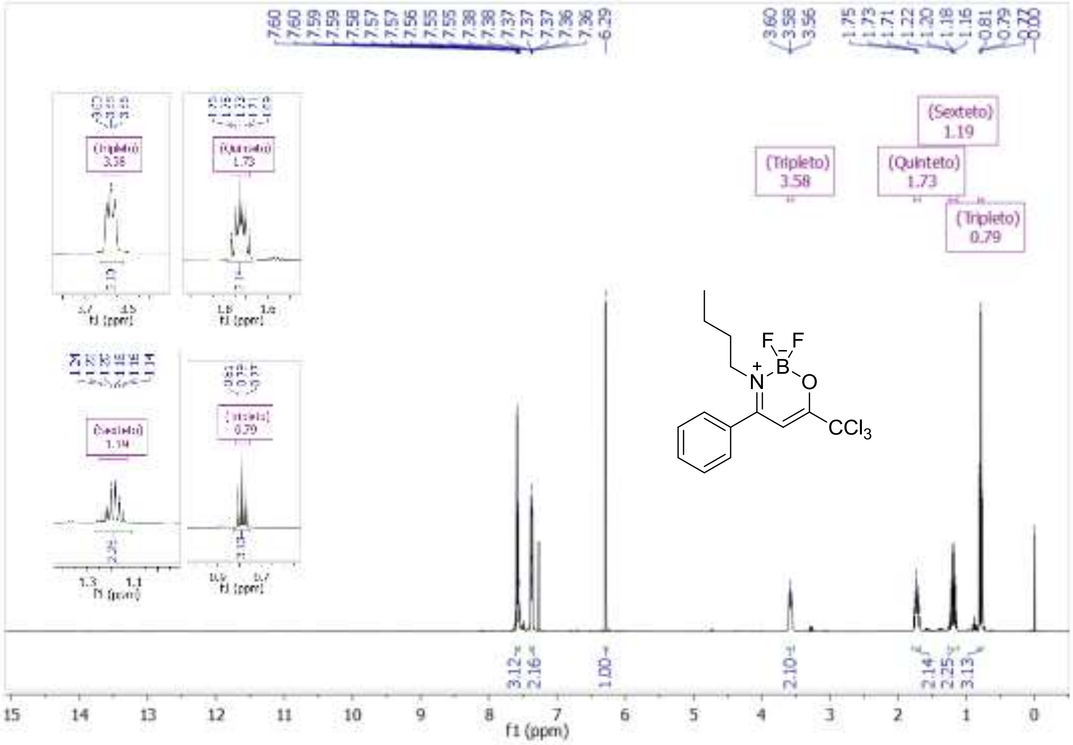

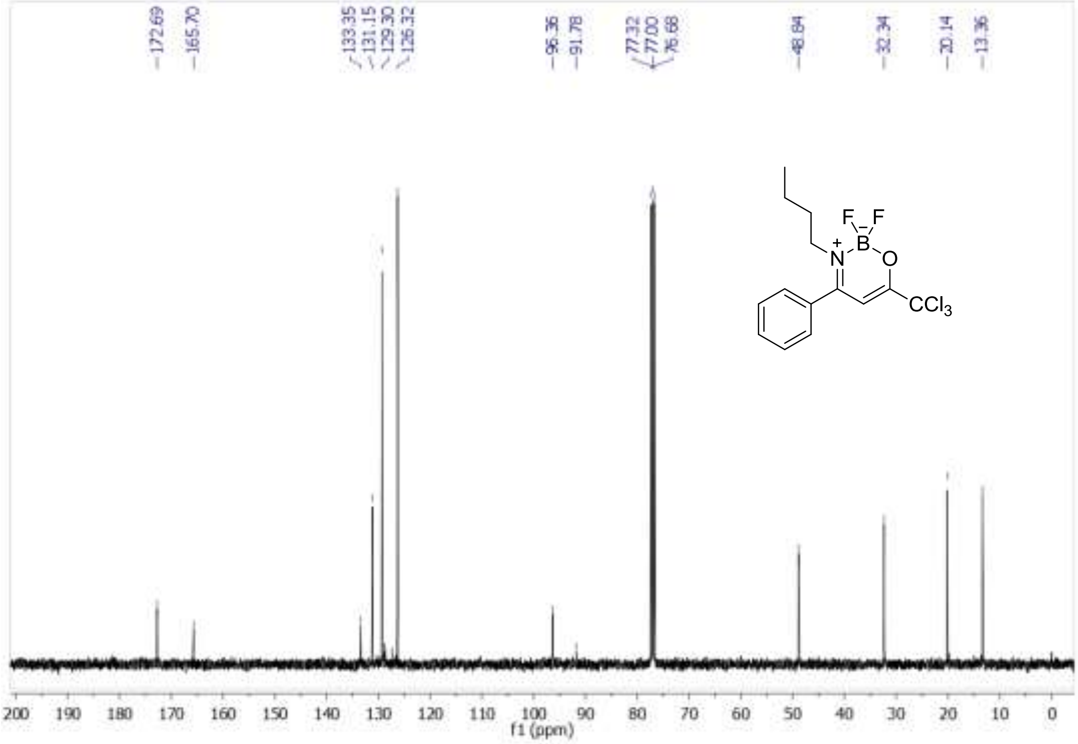

Figure S13. ^{1}H- and ^{13}C-NMR in $CDCl_3$ at 400 and 101 MHz, respectively of compound **6b**.

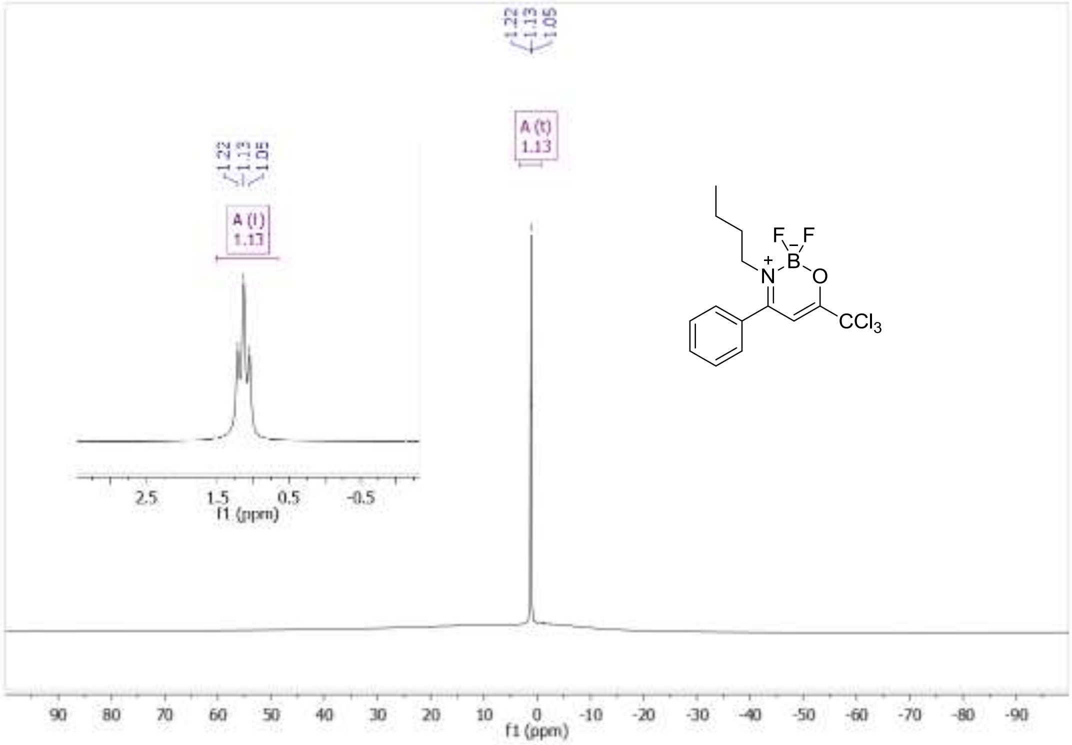

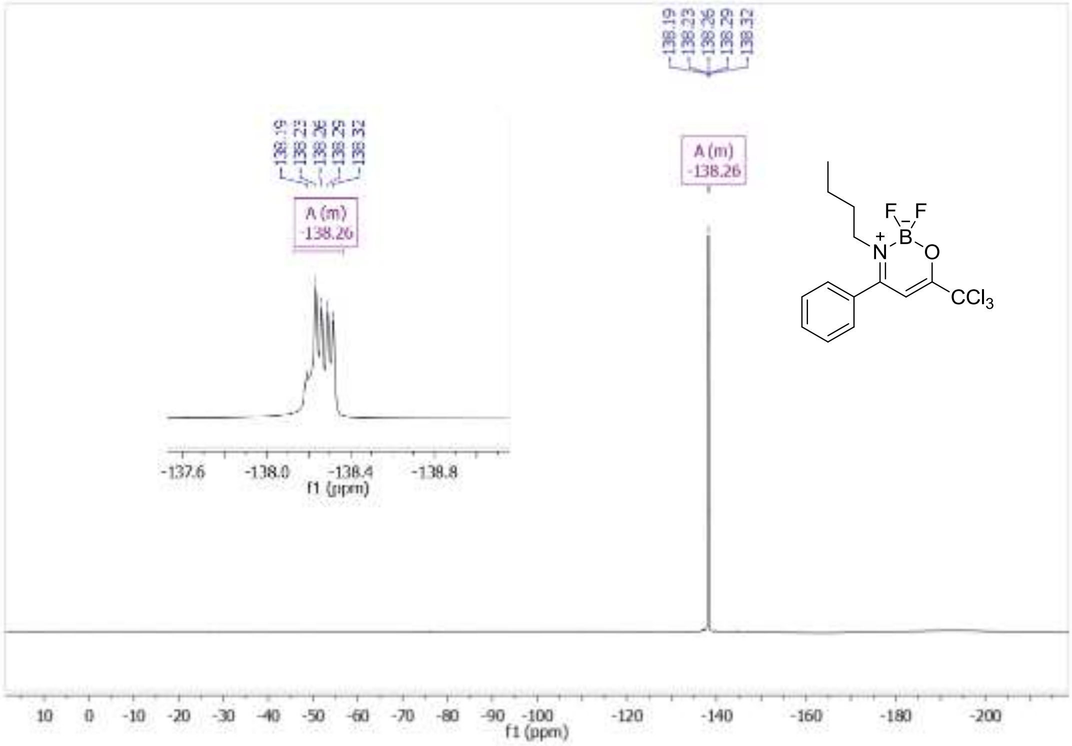

Figure S14. ^{11}B and ^{19}F-NMR in $CDCl_3$ at 193 and 565 MHz, respectively of compound **6b**.

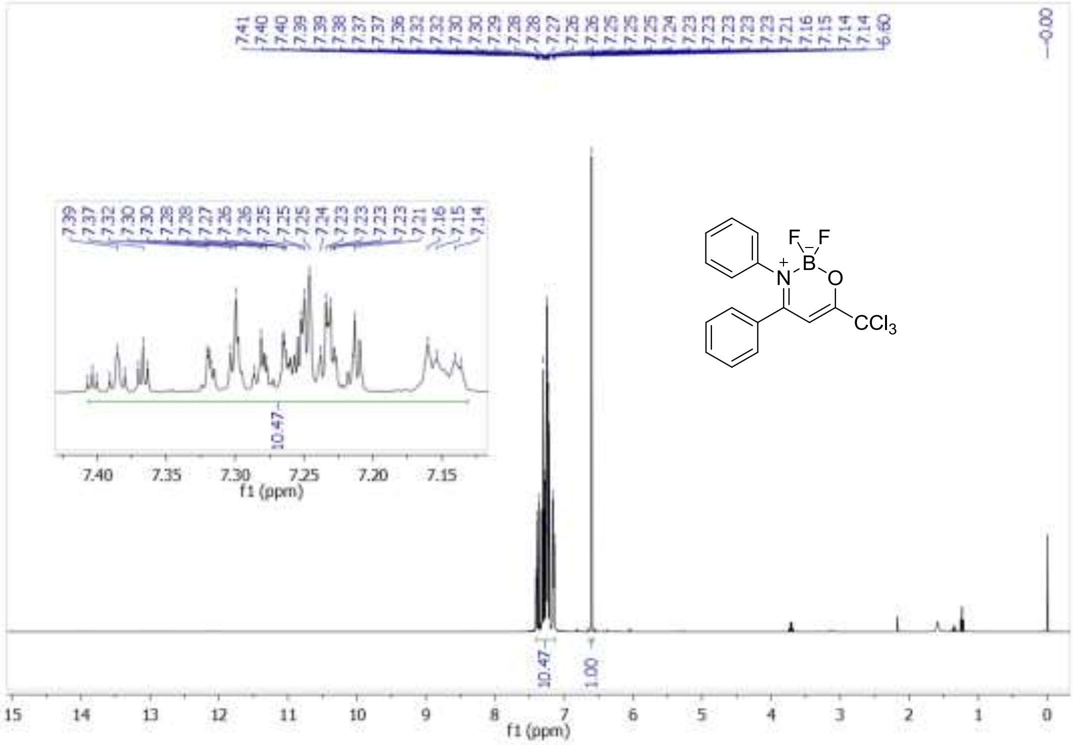

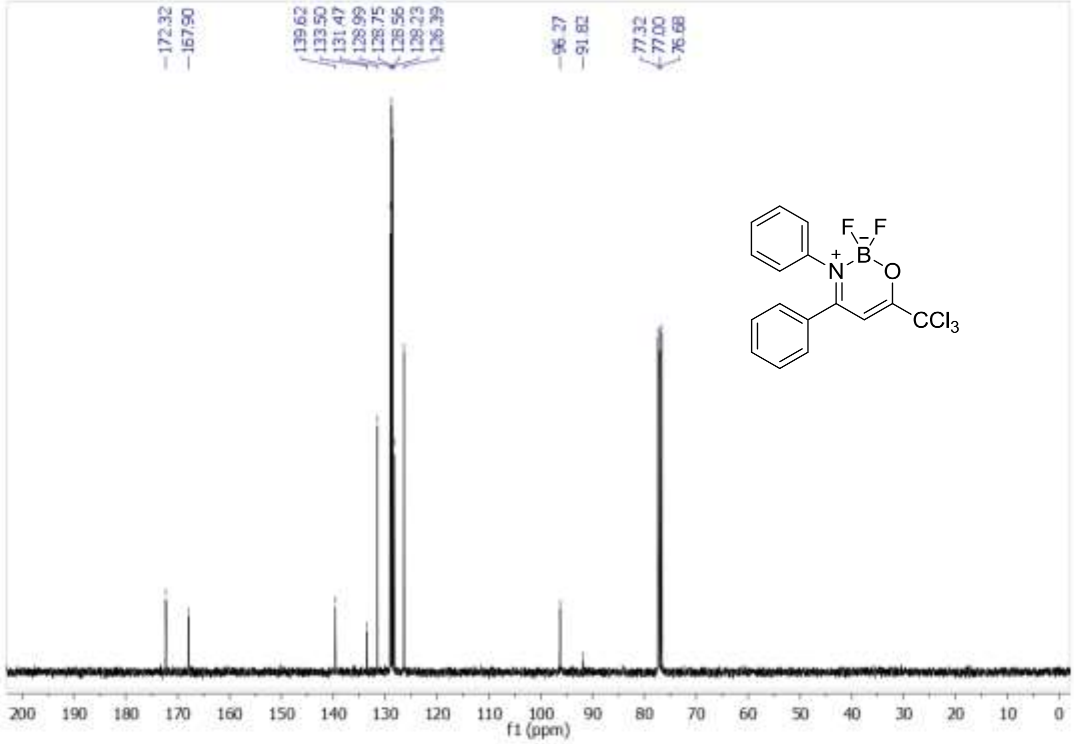

Figure S15. ^{1}H- and ^{13}C-NMR in $CDCl_3$ at 400 and 101 MHz, respectively of compound **6c**.

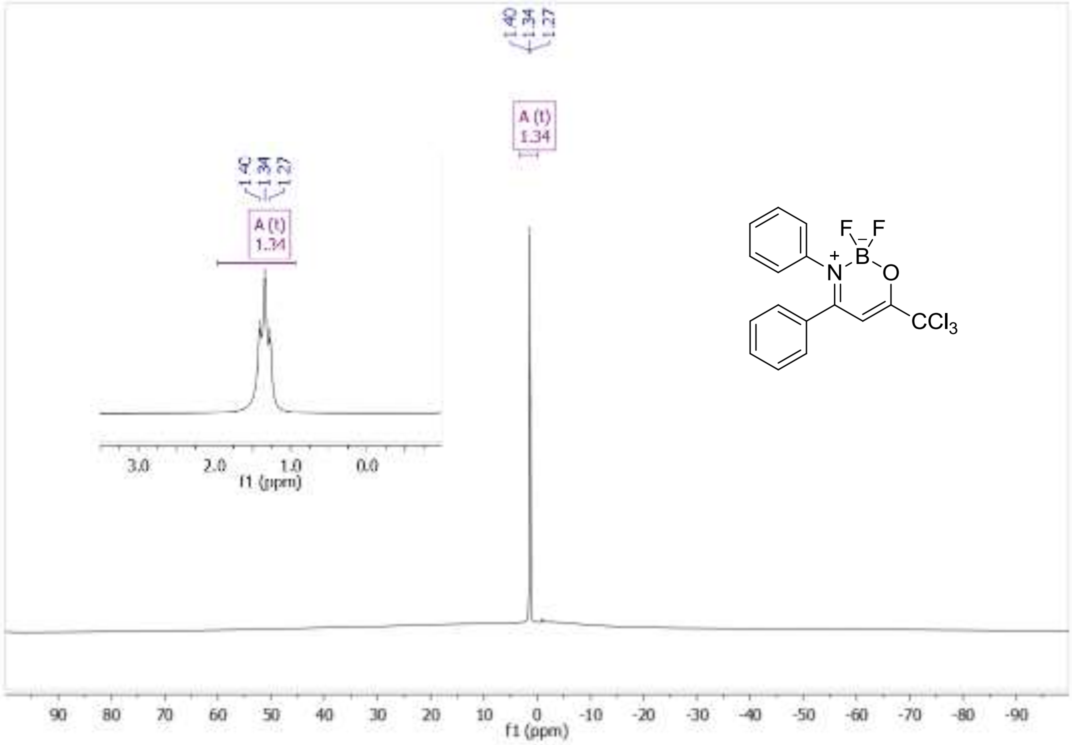

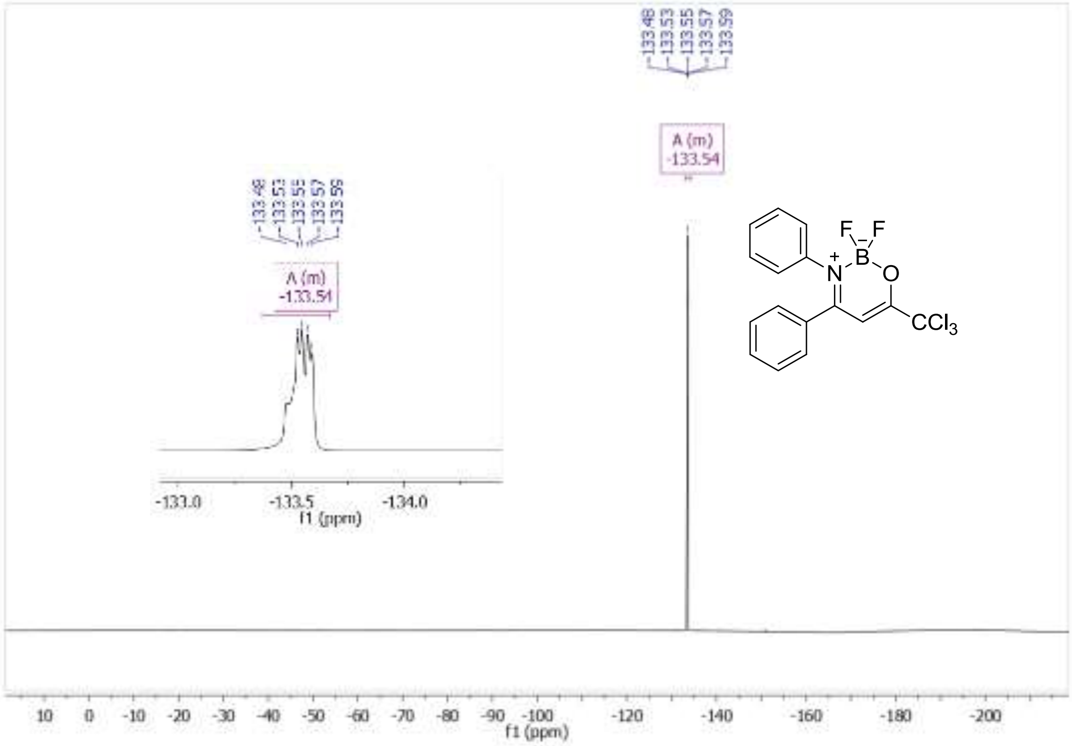

Figure S16. ^{11}B- and ^{19}F-NMR in $CDCl_3$ at 193 and 565 MHz respectively of compound **6c**.

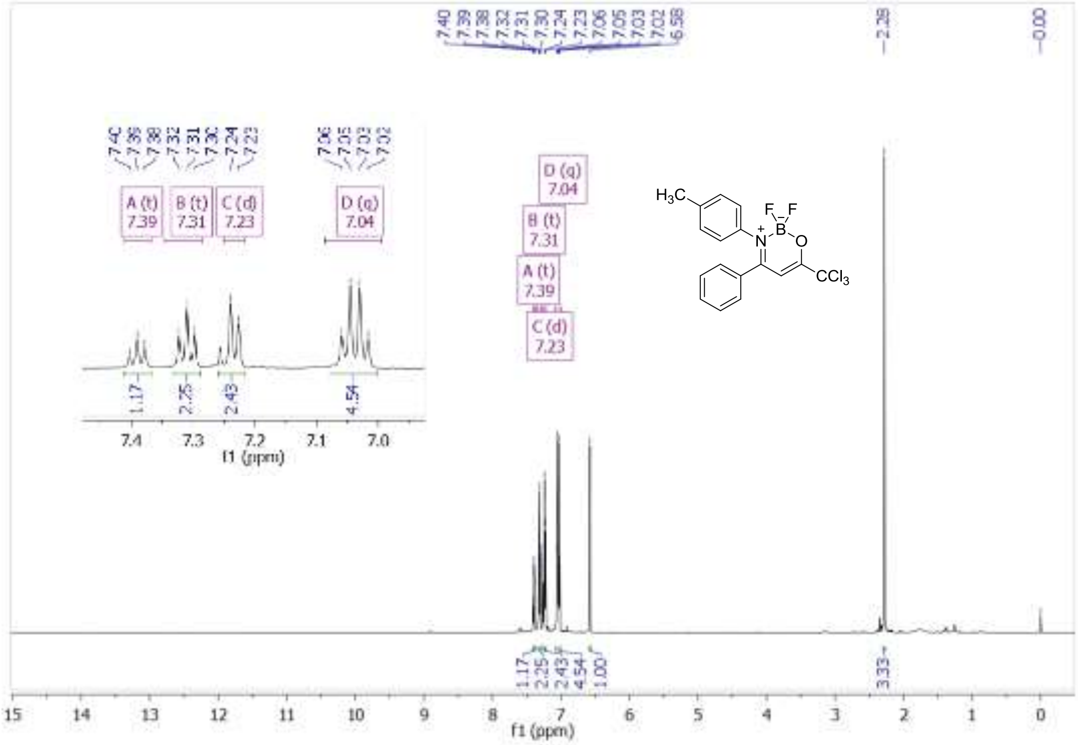

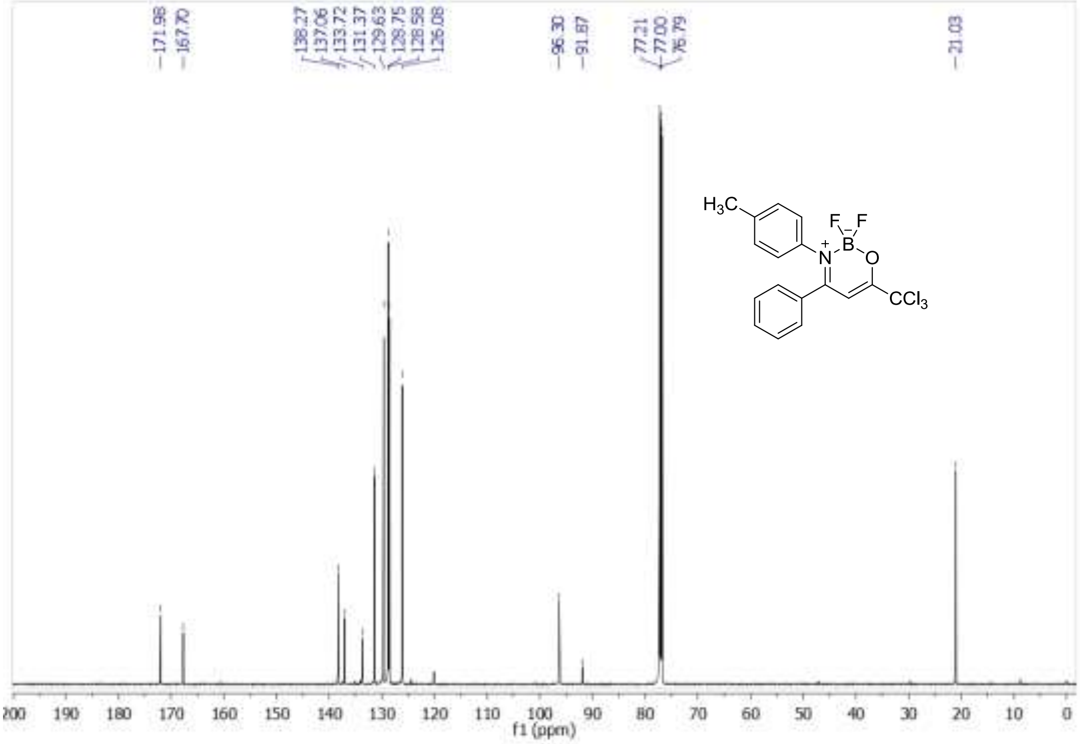

Figure S17. ^{1}H- and ^{13}C-NMR in $CDCl_3$ at 600 and 151 MHz, respectively of compound **6d**.

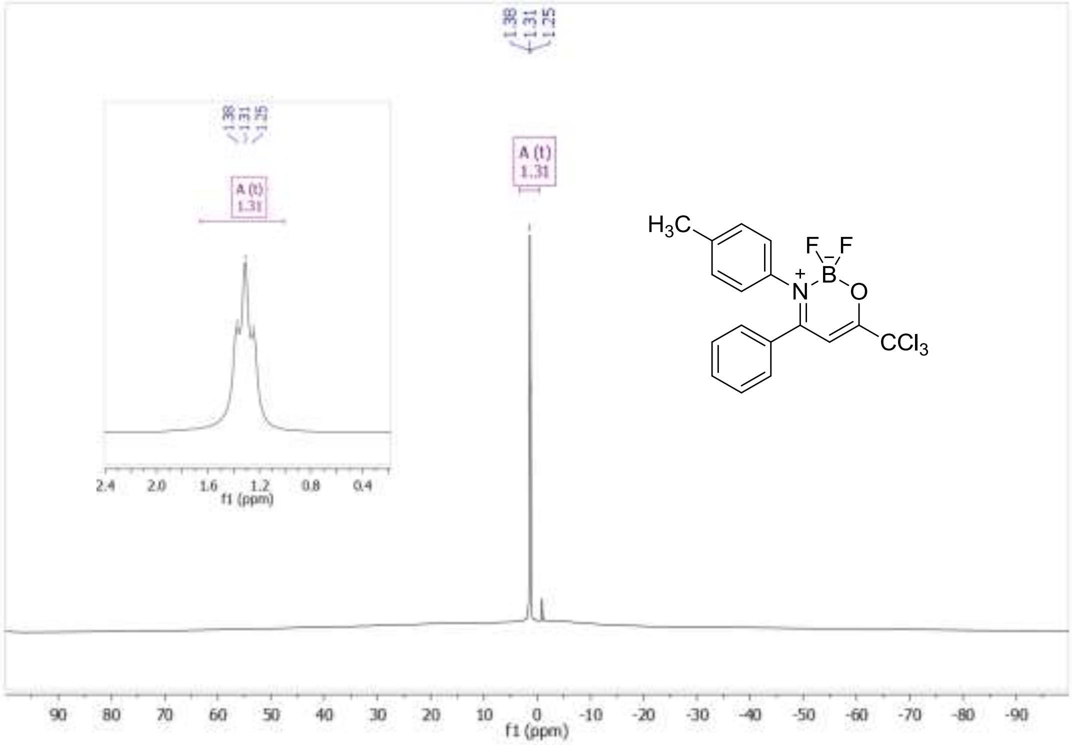

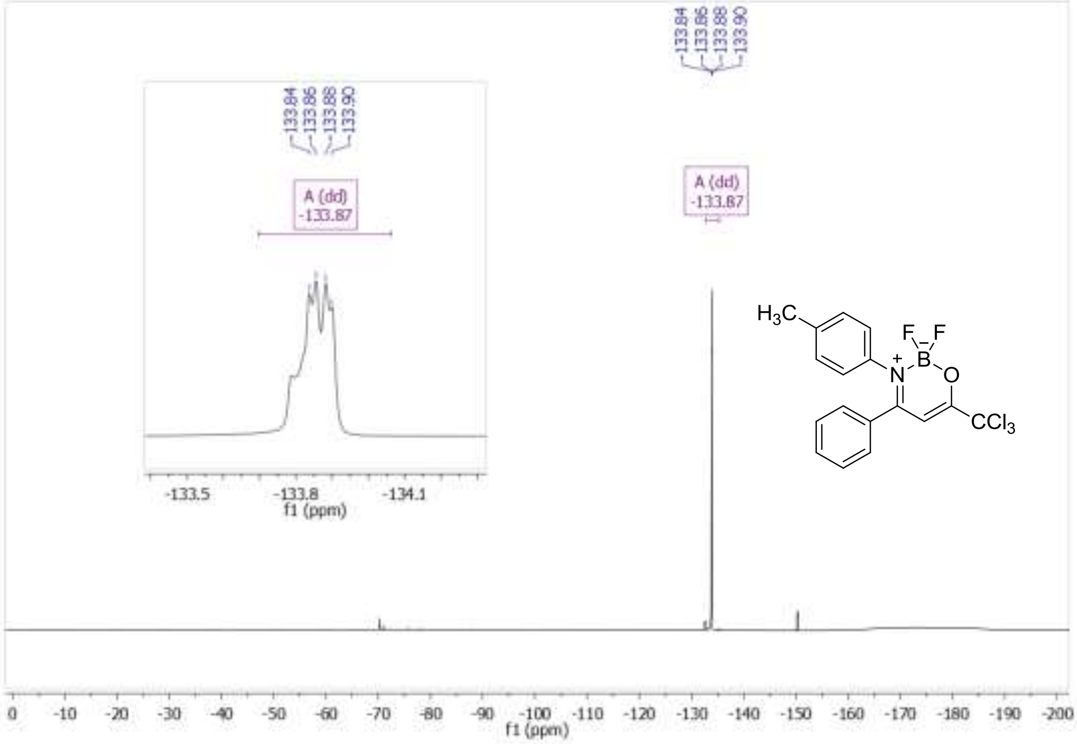

Figure S18. ^{11}B- and ^{19}F-NMR in $CDCl_3$ at 193 and 565 MHz, respectively of compound **6d**.

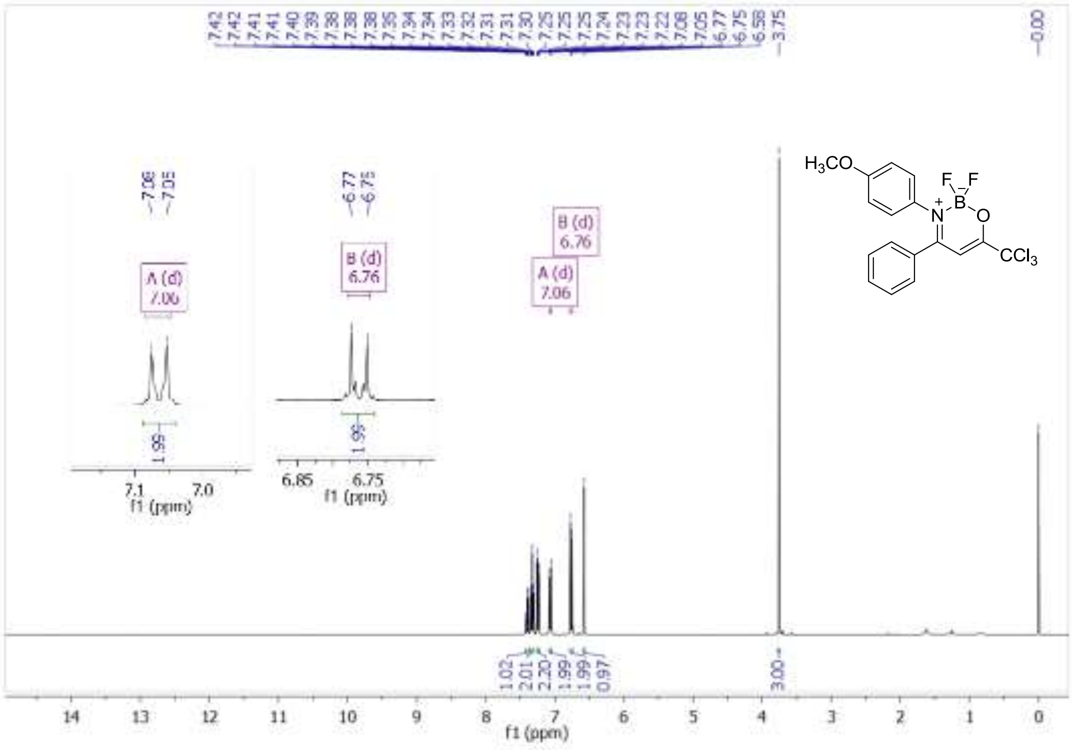

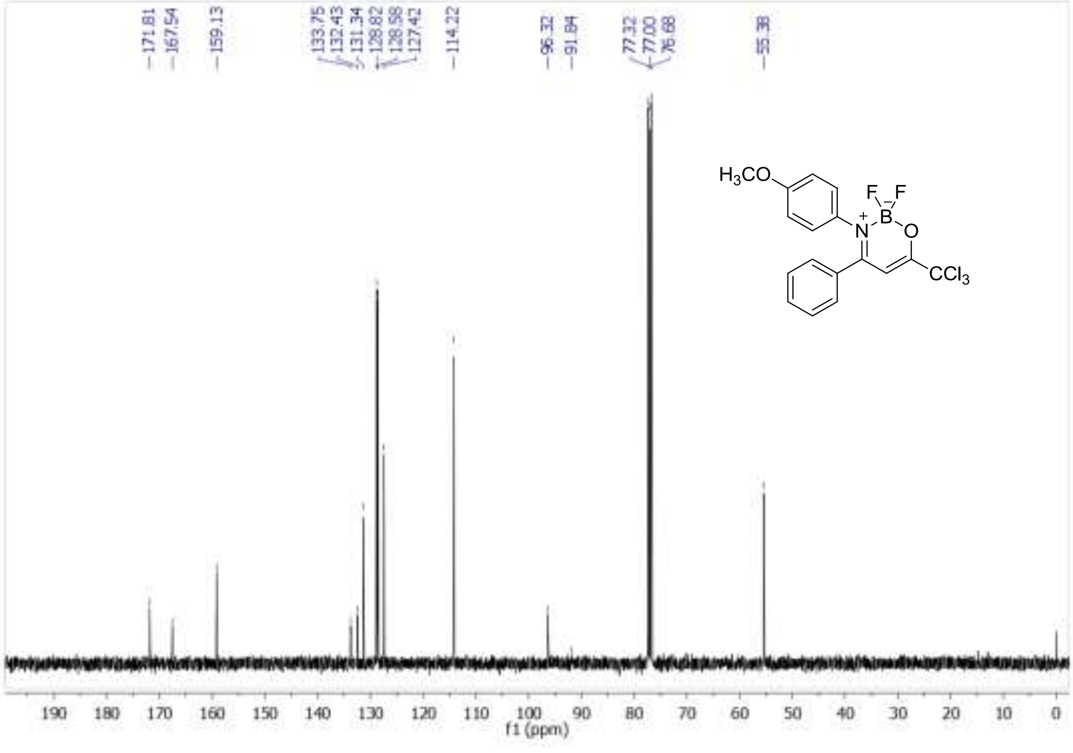

Figure S19. ^{1}H- and ^{13}C-NMR in $CDCl_3$ at 400 and 101 MHz, respectively of compound **6e**.

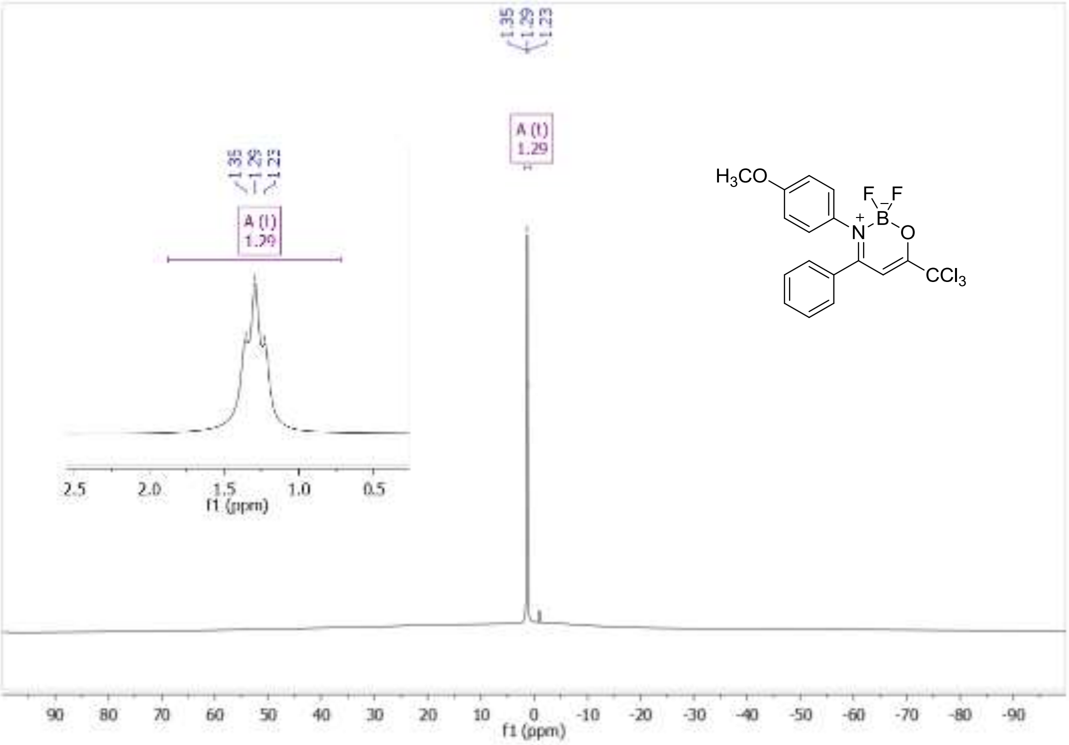

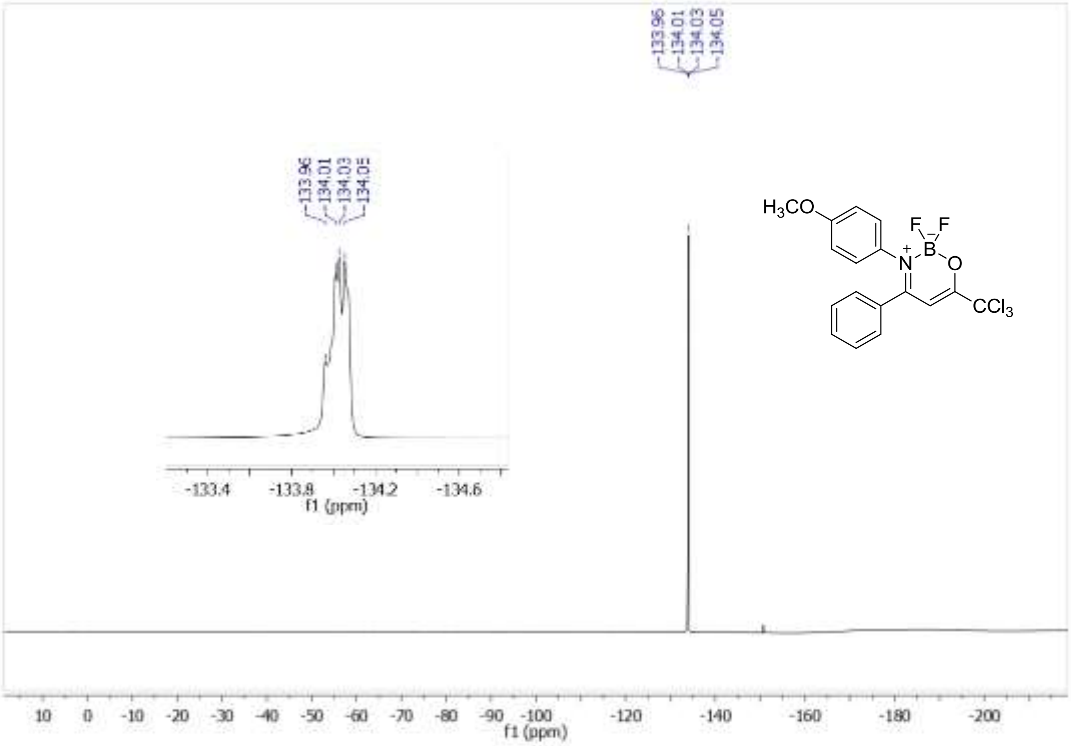

Figure S20. ^{11}B- and ^{19}F-NMR in $CDCl_3$ at 193 and 565 MHz, respectively of compound **6e**.

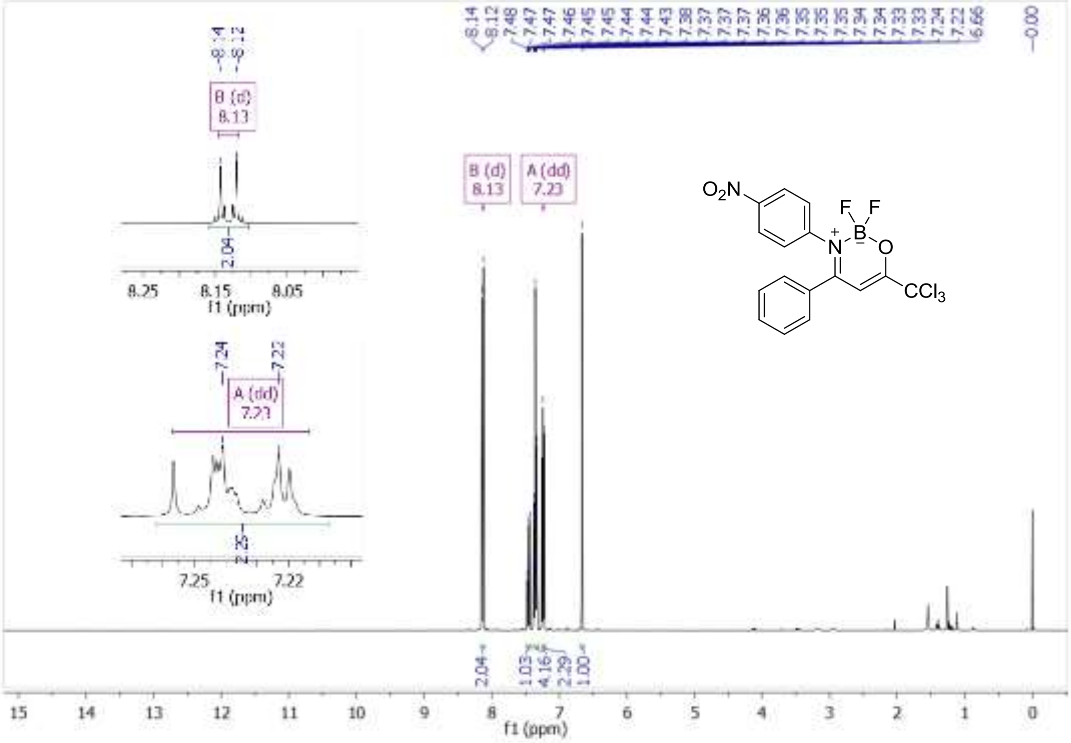

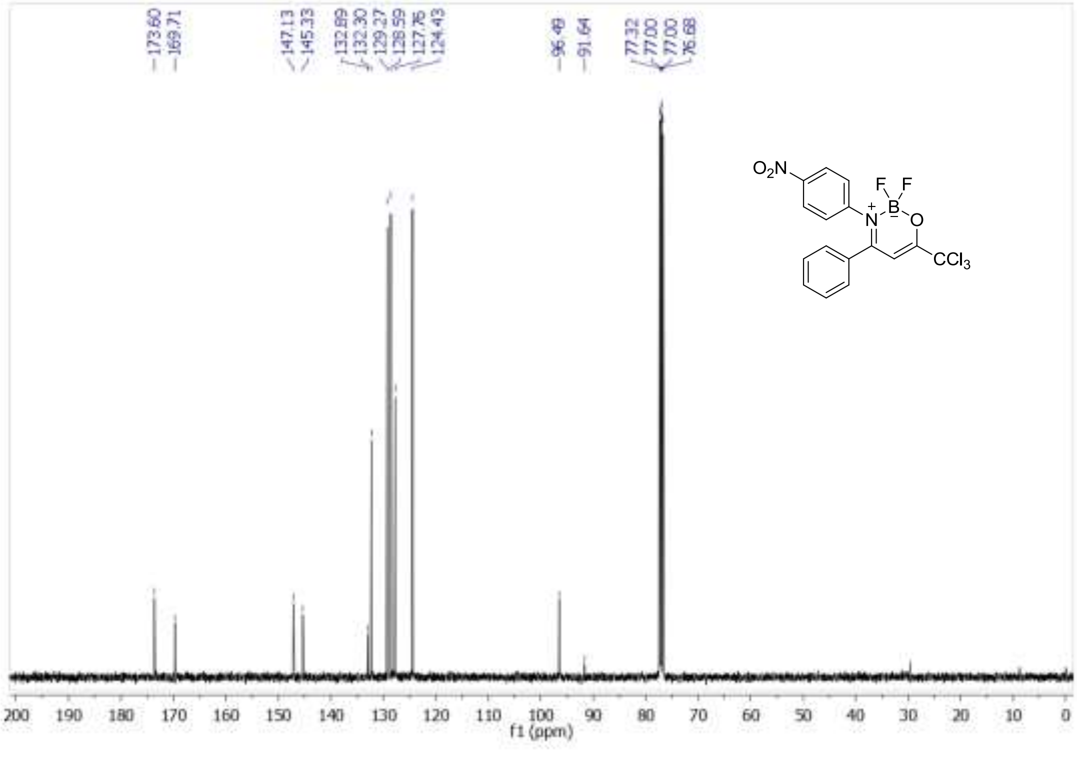

Figure S21. ^{1}H- and ^{13}C-NMR in $CDCl_3$ at 400 and 101 MHz, respectively of compound **6f**.

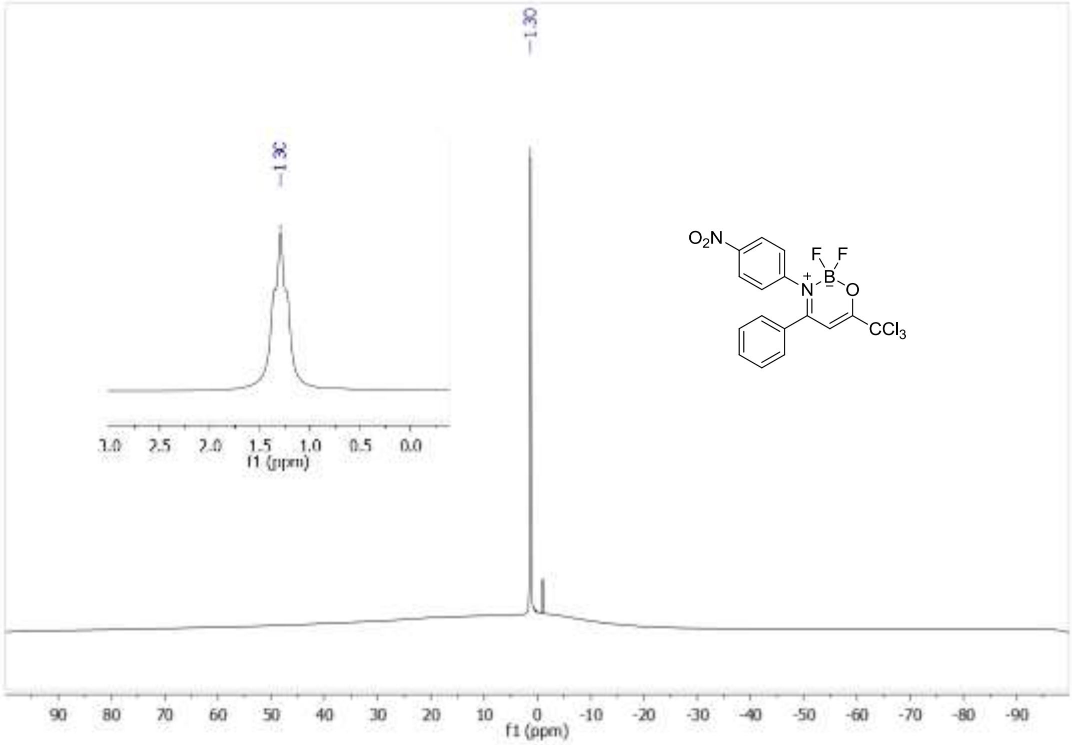

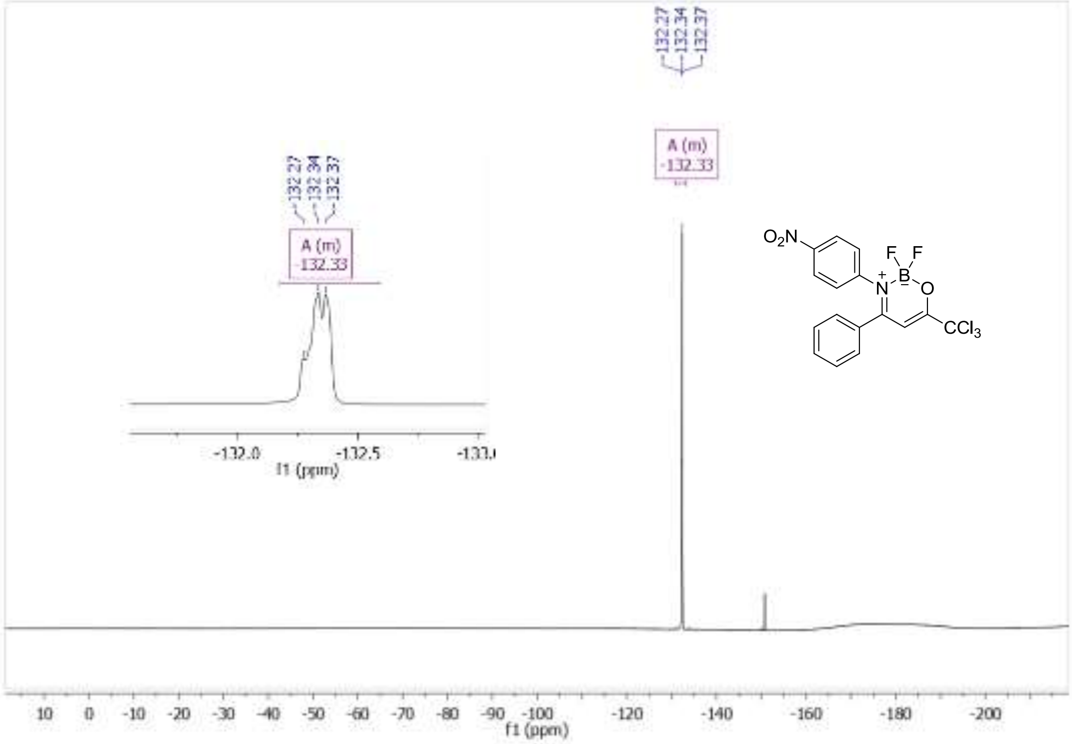

Figure S22. ^{11}B- and ^{19}F-NMR in $CDCl_3$ at 193 and 565 MHz, respectively of compound **6f**.

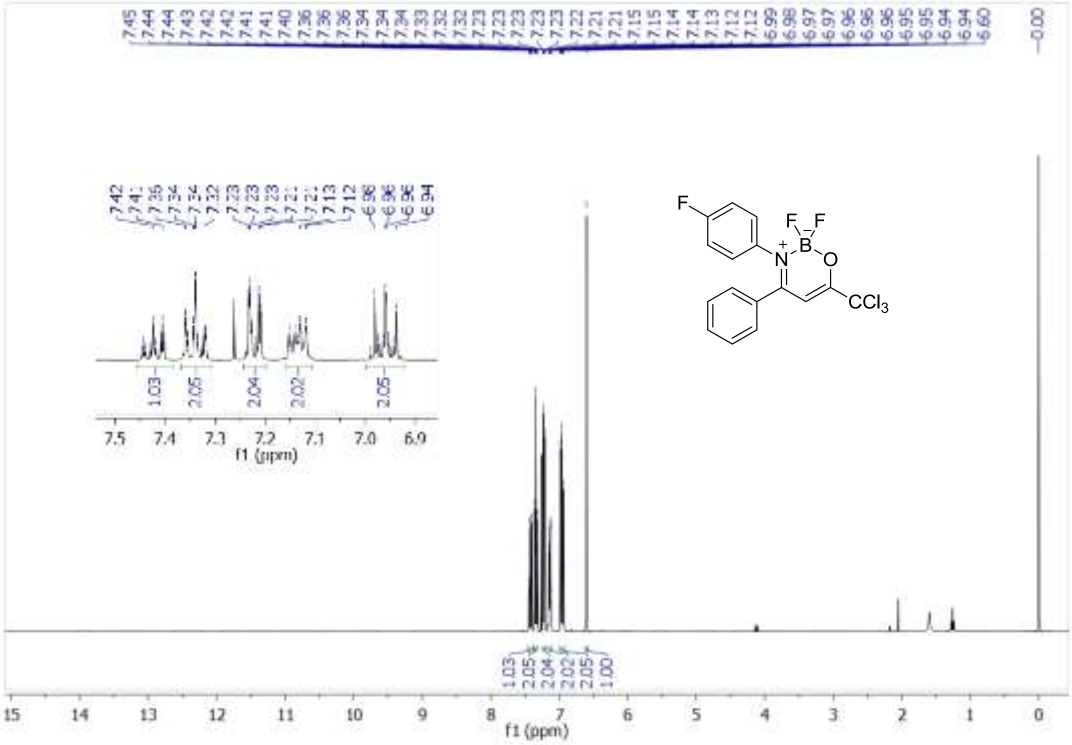

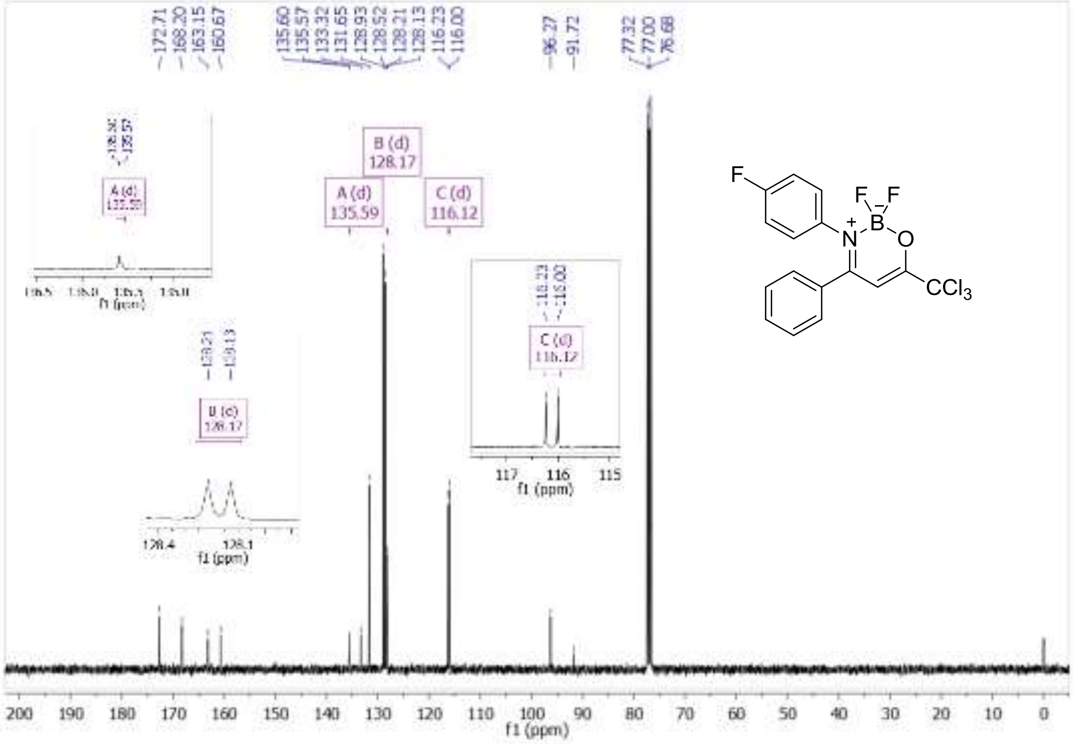

Figure S23. ^{1}H- and ^{13}C-NMR in $CDCl_3$ at 400 and 101 MHz, respectively of compound **6g**.

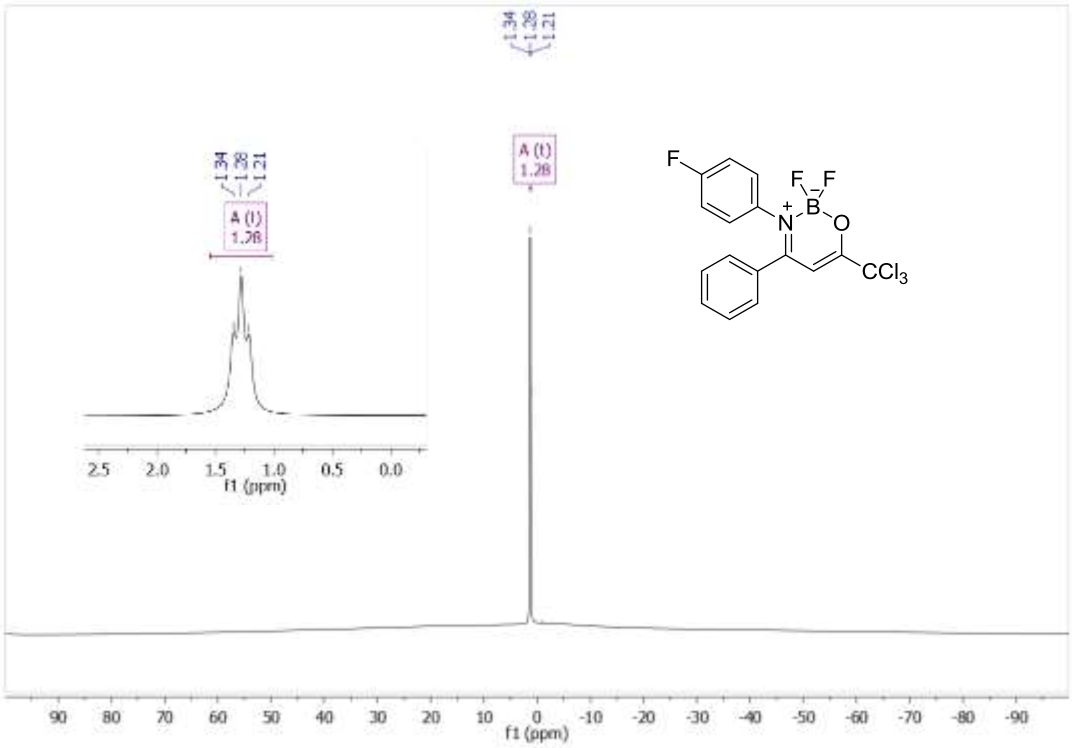

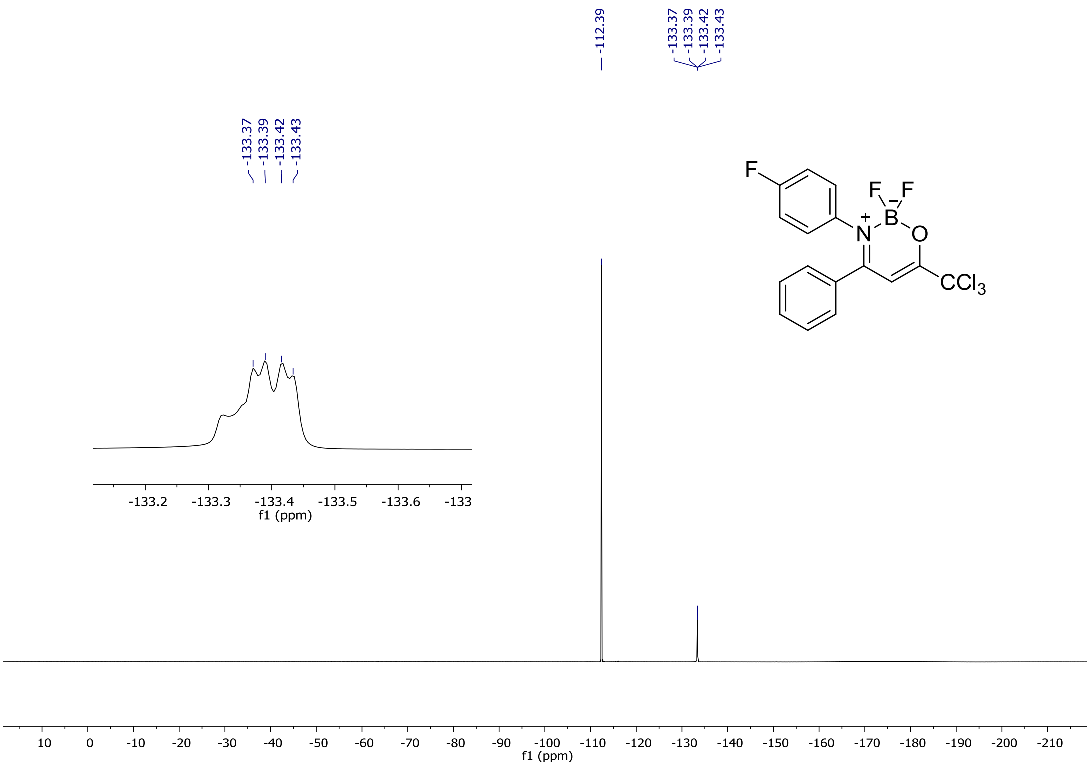

Figure S24. ^{11}B- and ^{19}F-NMR in $CDCl_3$ at 193 and 565 MHz, respectively of compound **6g**.

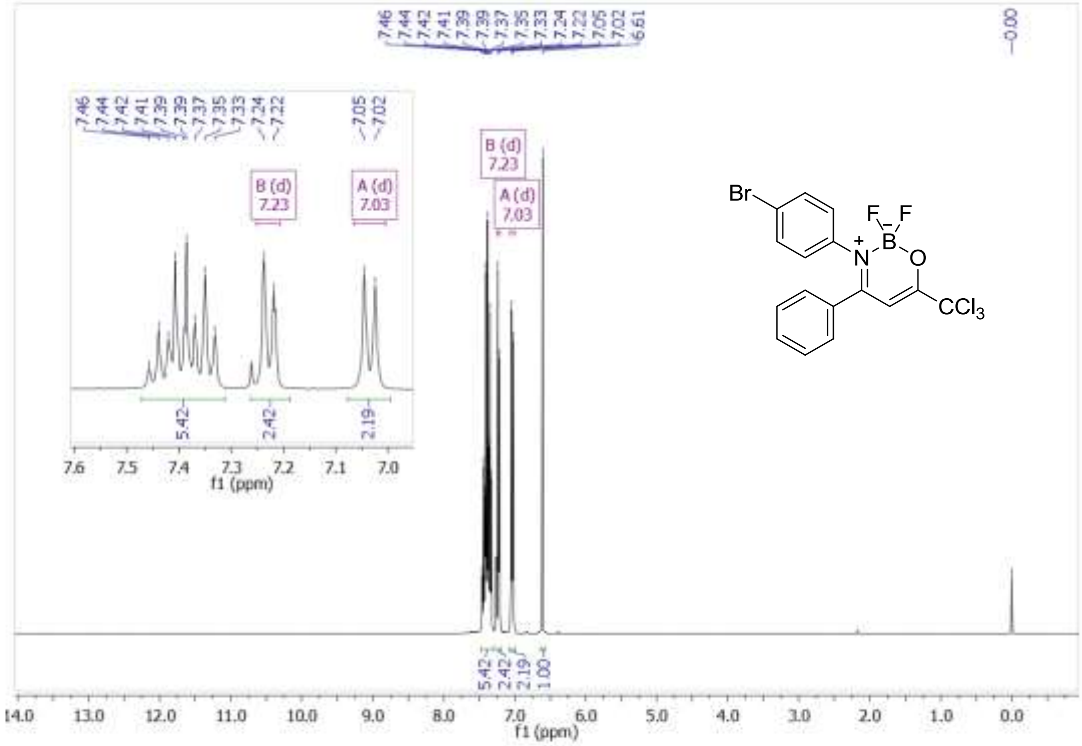

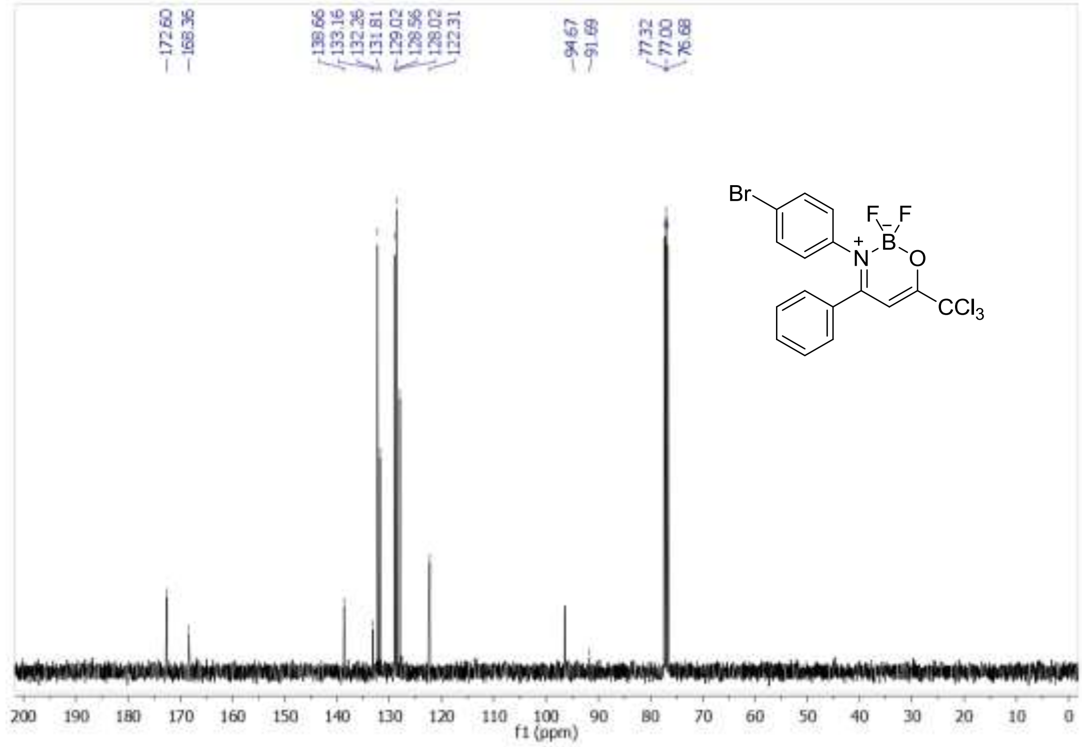

Figure S25. ^{1}H- and ^{13}C-NMR in $CDCl_3$ at 400 and 101 MHz, respectively of compound **6h**.

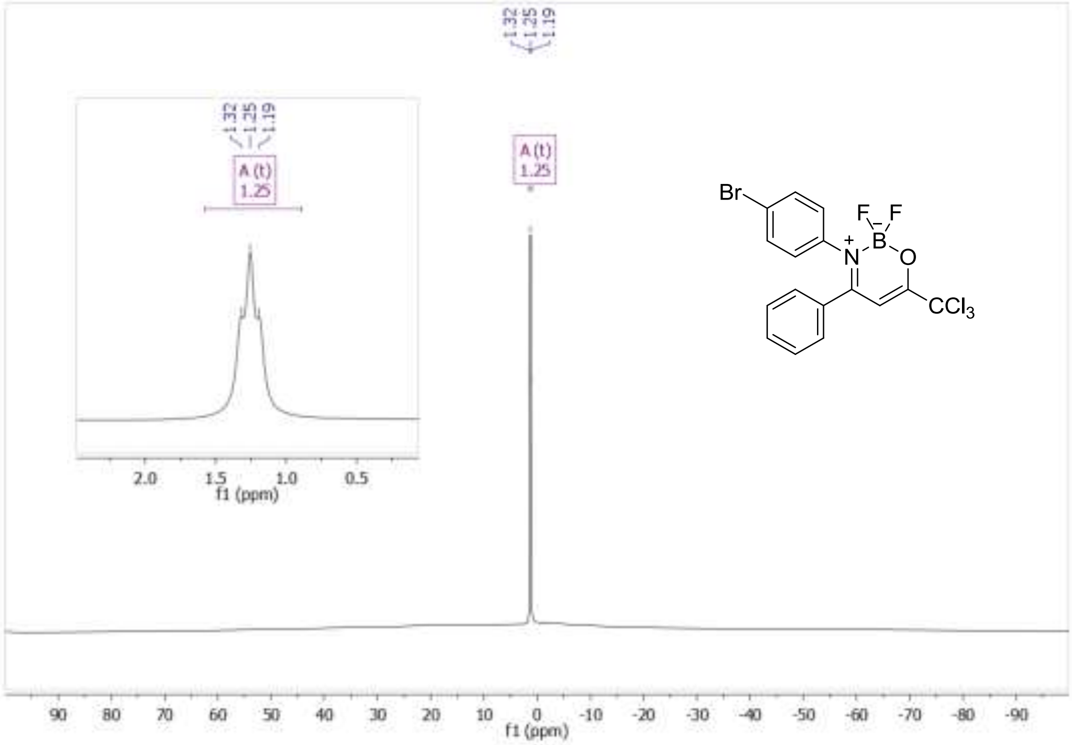

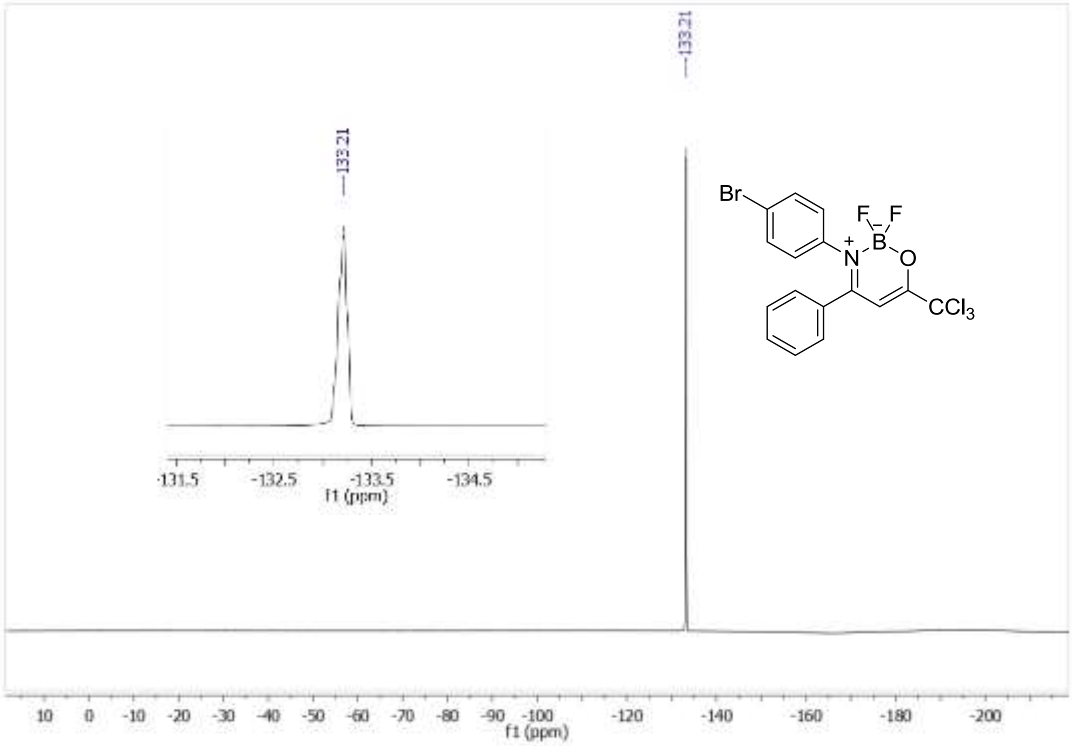

Figure S26. ^{11}B- and ^{19}F-NMR in $CDCl_3$ at 193 and 565 MHz, respectively of compound **6h**.

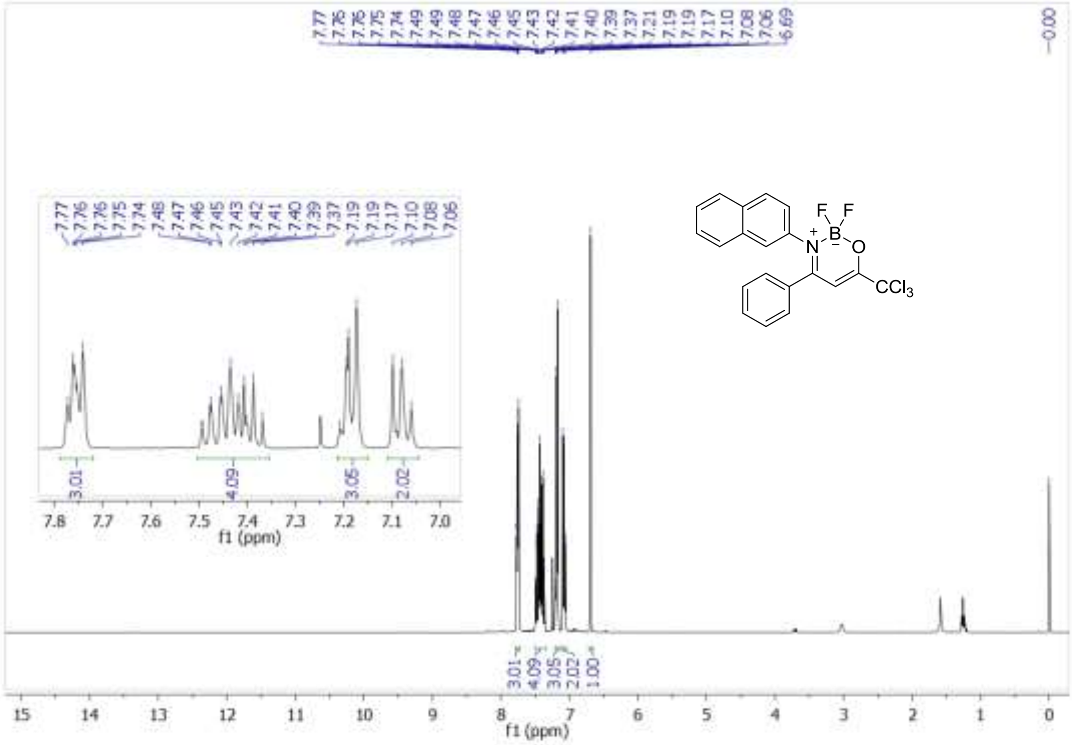

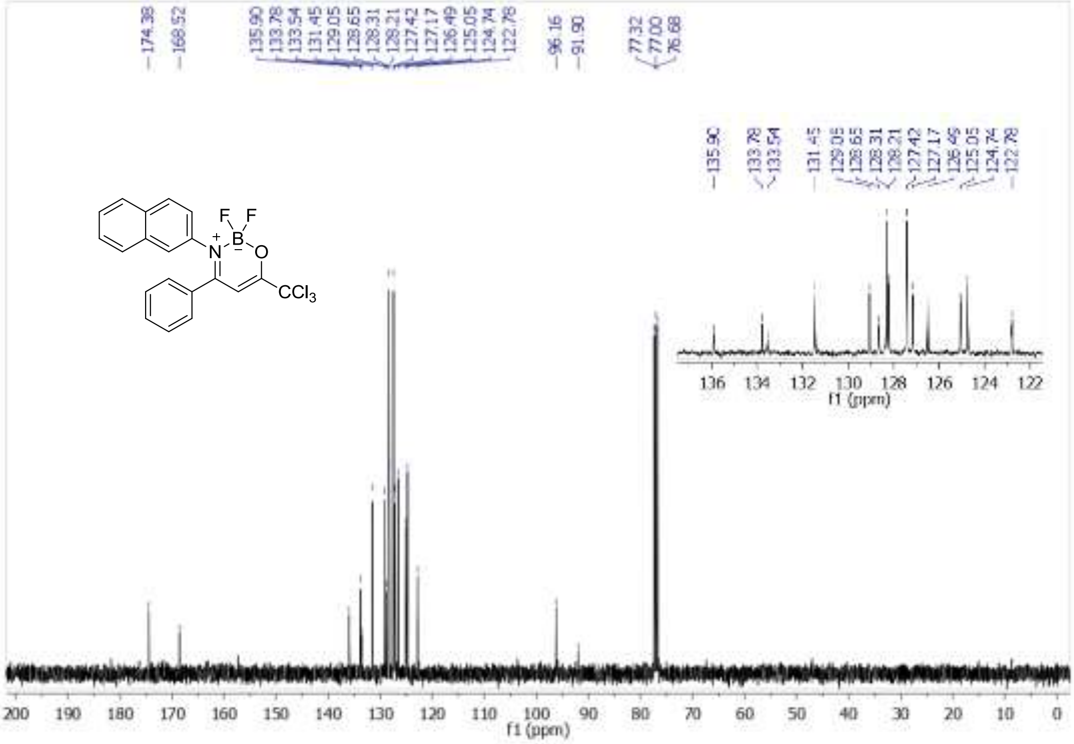

Figure S27. ^{1}H- and ^{13}C-NMR in $CDCl_3$ at 400 and 101 MHz respectively of compound **6i**.

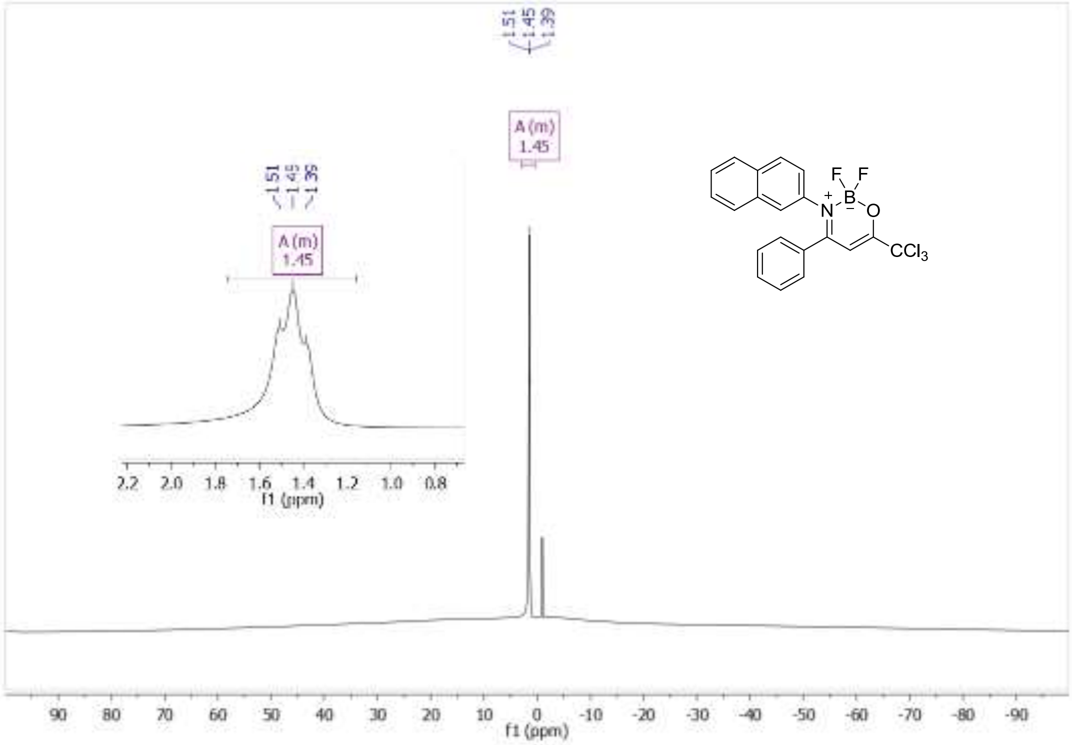

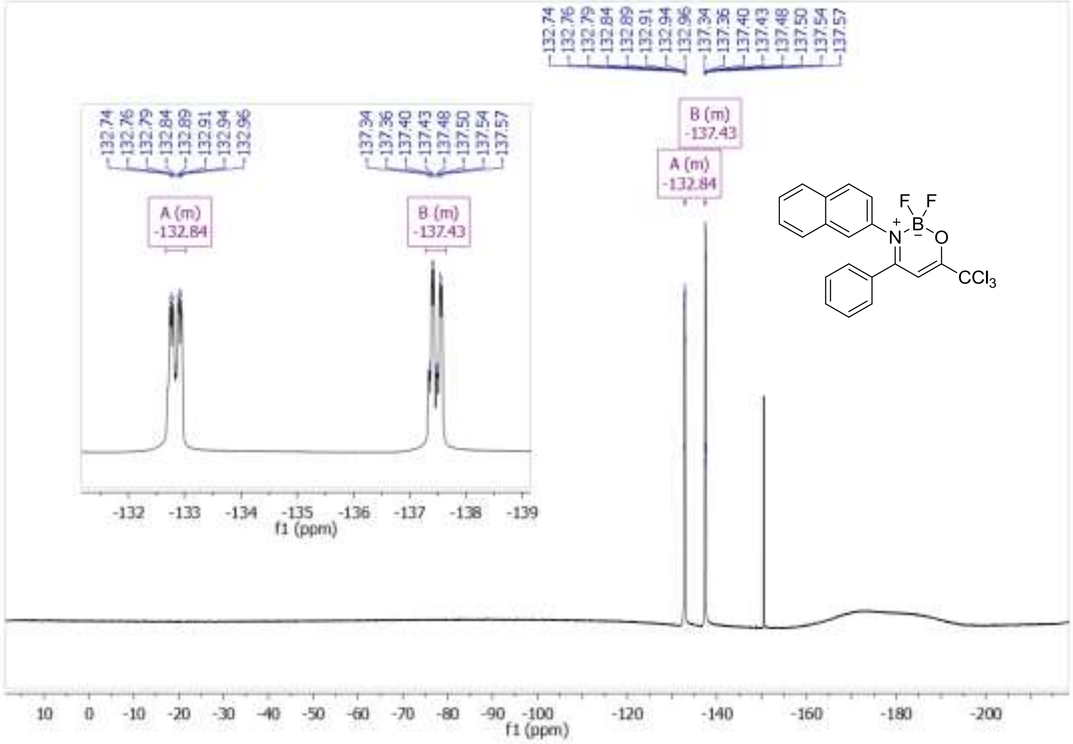

Figure S28. ^{11}B- and ^{19}F-NMR in $CDCl_3$ at 193 and 565 MHz respectively of compound **6i**.

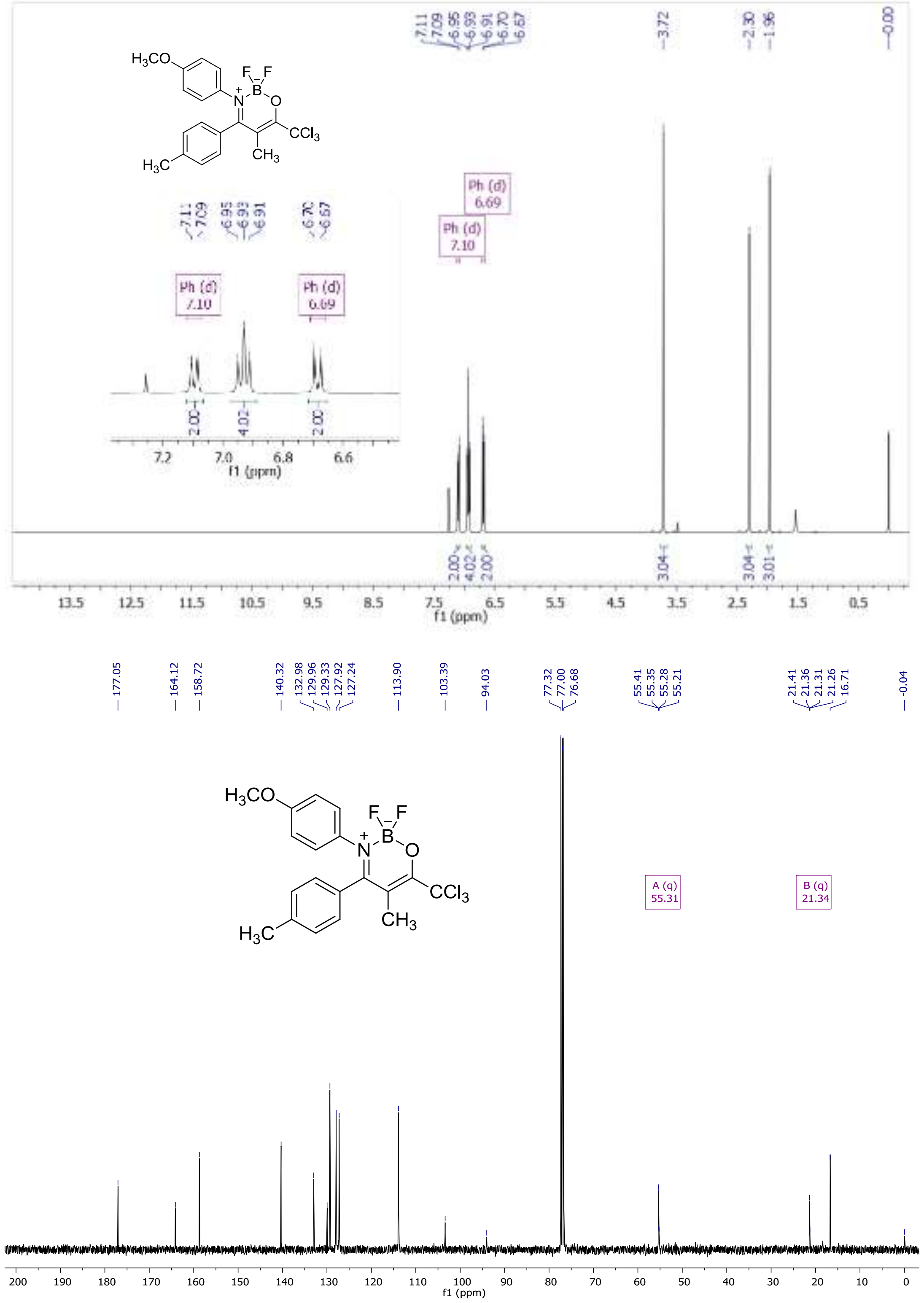

Figure S29. ^{1}H- and ^{13}C-NMR in $CDCl_3$ at 400 and 101 MHz, respectively of compound **7e**.

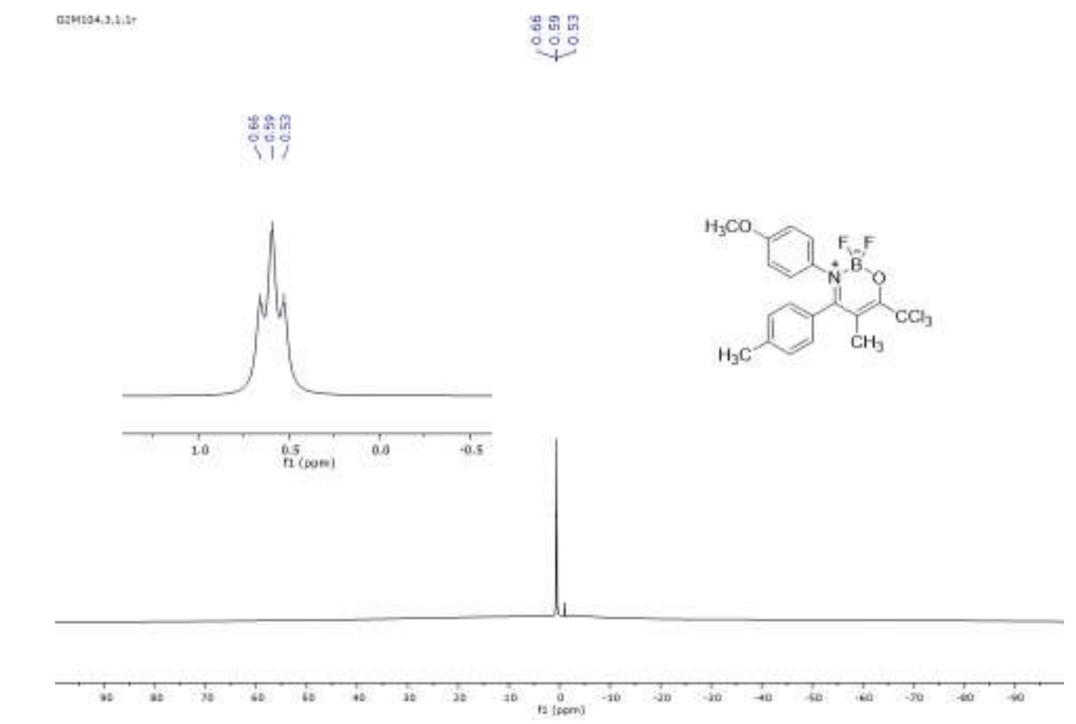

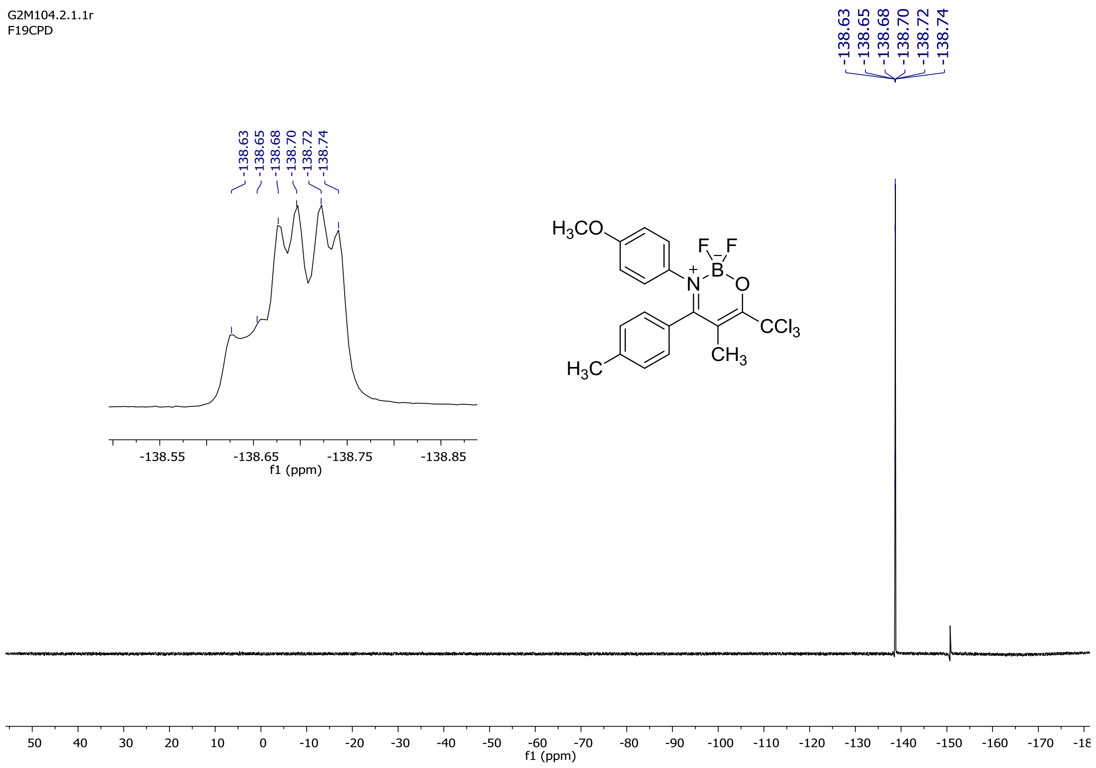

Figure S30. ^{11}B- and ^{19}F-NMR in $CDCl_3$ at 193 and 565 MHz, respectively of compound **7e**.

2. HMRS spectra of compounds 4c, 4d, 4e and 4g.

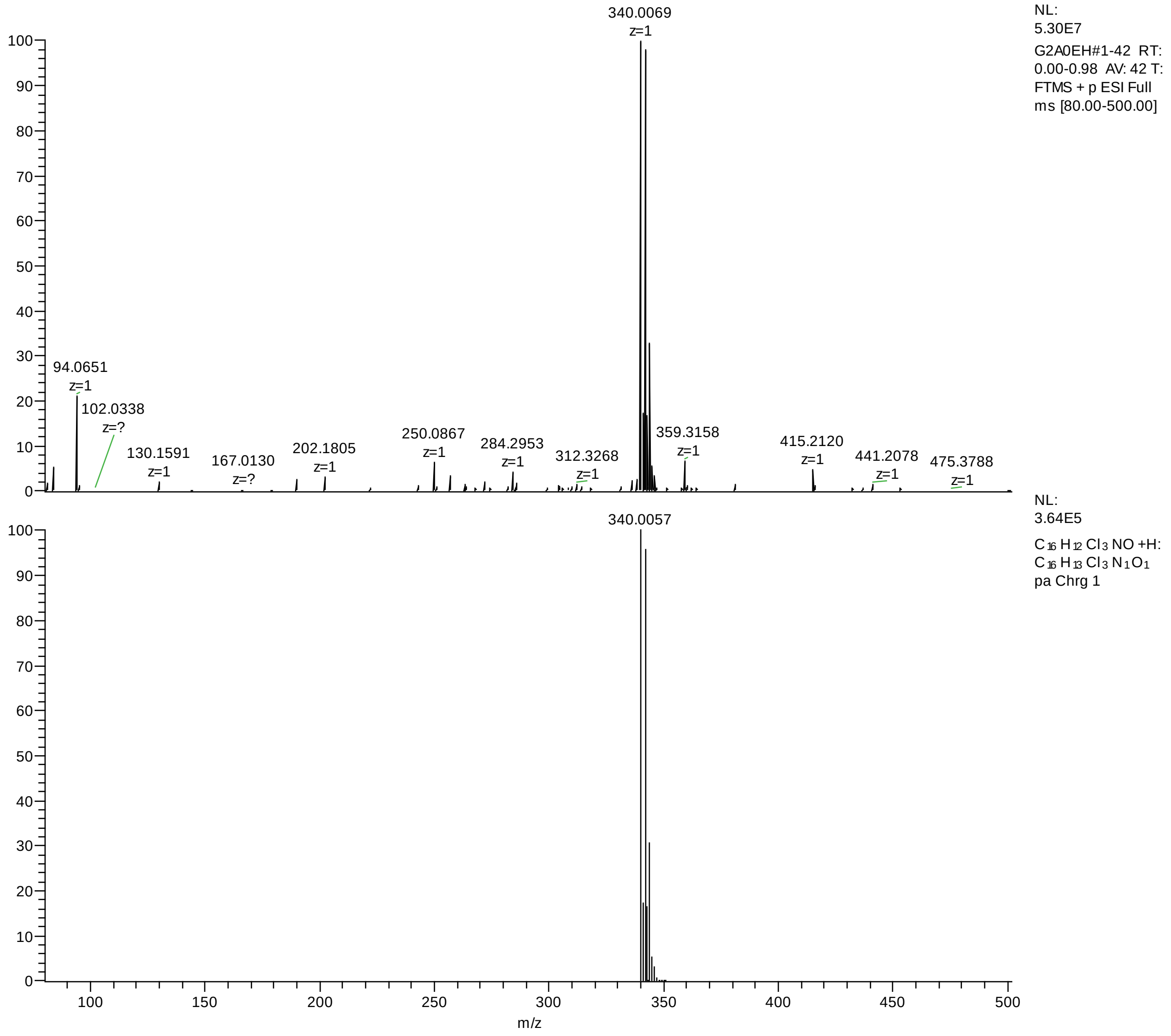

Figure S31. HRMS of compound **4c**.

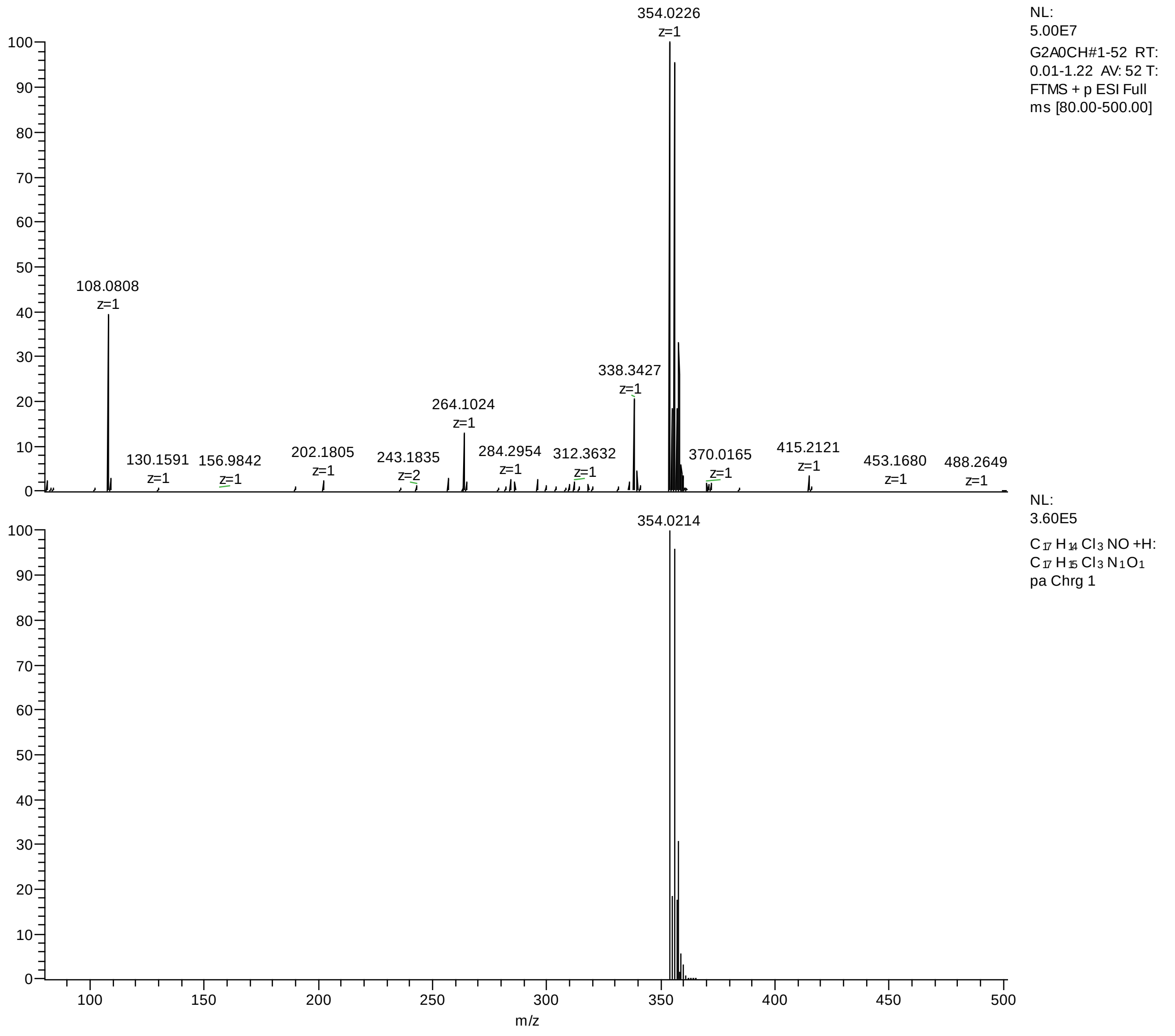

Figure S32. HRMS of compound **4d**.

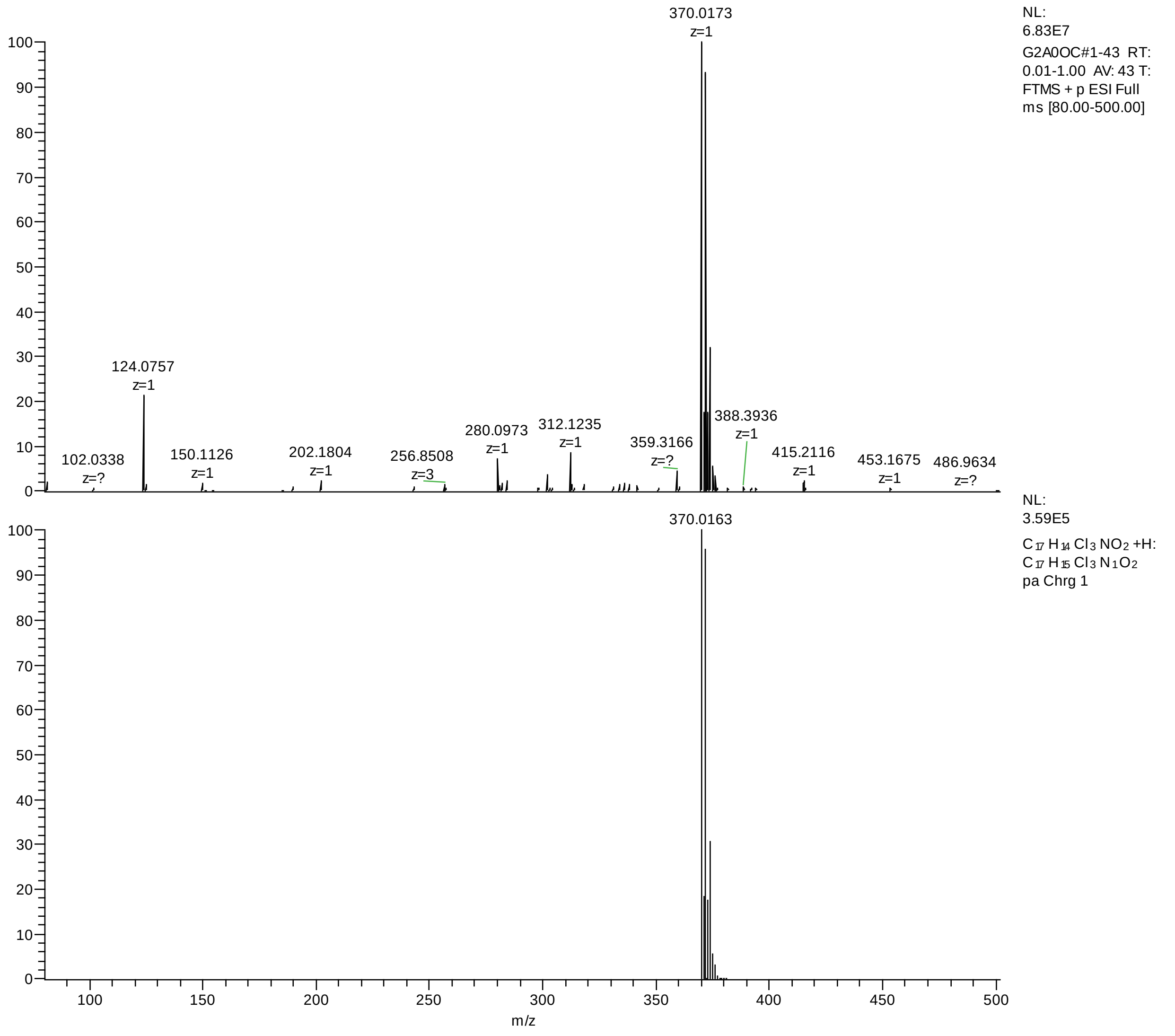

Figure S33. HRMS of compound **4e**.

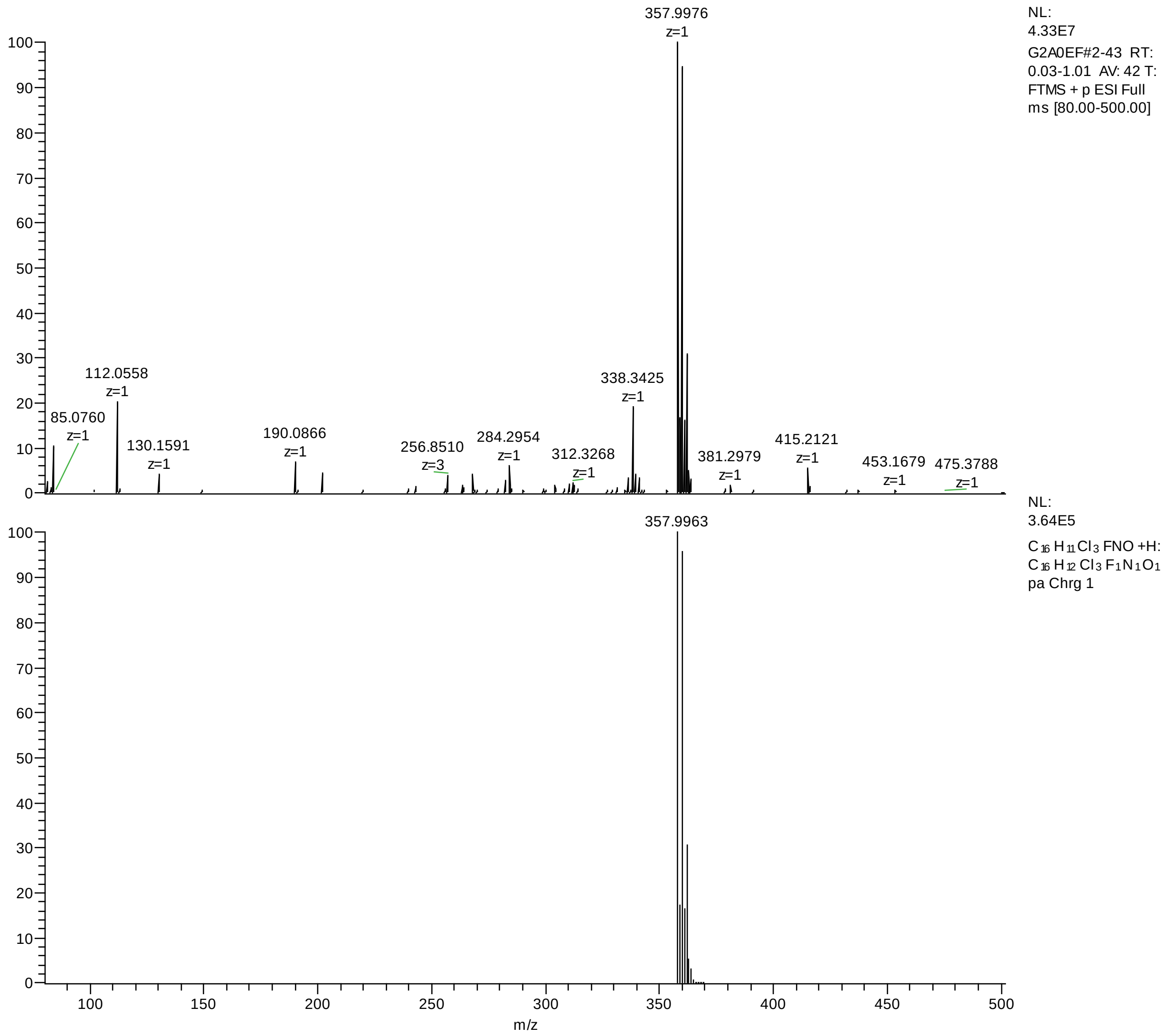

Figure S34. HRMS of compound **4g**.

3. Single Crystal X-Ray Data of compounds 4g and 6e.

Single Crystal X-Ray Data of compound 4g.

checkCIF/PLATON report

You have not supplied any structure factors. As a result the full set of tests cannot be run.

THIS REPORT IS FOR GUIDANCE ONLY. IF USED AS PART OF A REVIEW PROCEDURE FOR PUBLICATION, IT SHOULD NOT REPLACE THE EXPERTISE OF AN EXPERIENCED CRYSTALLOGRAPHIC REFEREE.

No syntax errors found. CIF dictionary Interpreting this report

Datablock: I

```
Bond precision:  C-C = 0.0049 A          Wavelength=1.54178

Cell:           a=5.9888(8)        b=11.5229(14)    c=12.3545(15)
                alpha=110.229(4)   beta=99.803(4)   gamma=93.479(4)
Temperature:    296 K

                 Calculated                Reported
Volume           781.68(17)                781.68(17)
Space group      P -1                      P-1
Hall group       -P 1                      -P1
Moiety formula   C16 H11 Cl3 F N O         C16 H11 Cl3 F N O
Sum formula      C16 H11 Cl3 F N O         C16 H11 Cl3 F N O
Mr               358.61                    358.61
Dx,g cm-3        1.524                     1.524
Z                2                         2
Mu (mm-1)        5.409                     5.409
F000             364.0                     364.0
F000'            367.06
h,k,lmax         7,13,14                   7,13,14
Nref             2887                      2712
Tmin,Tmax        0.199,0.399               0.261,0.460
Tmin'            0.127

Correction method= # Reported T Limits: Tmin=0.261 Tmax=0.460
AbsCorr = GAUSSIAN

Data completeness= 0.939          Theta(max)= 68.480

R(reflections)= 0.0600( 2487)     wR2(reflections)= 0.1757( 2712)

S = 1.095                Npar= 199
```

The following ALERTS were generated. Each ALERT has the format
test-name_ALERT_alert-type_alert-level.
Click on the hyperlinks for more details of the test.

Alert level C

```
ABSTY02_ALERT_1_C  An _exptl_absorpt_correction_type has been given without
           a literature citation. This should be contained in the
           _exptl_absorpt_process_details field.
           Absorption correction given as gaussian
DIFMX02_ALERT_1_C  The maximum difference density is > 0.1*ZMAX*0.75
           The relevant atom site should be identified.
HYDTR01_ALERT_1_C  The hydrogen treatment should only be one of the following
           keywords
           *  refall
           *  refxyz
           *  refU
           *  noref
           *  undef
           *  constr
           *  none
           *  mixed
           *  hetero
           *  heteroxyz
           *  heteroU
           *  heteronoref
           *  hetero-mixed
           *  heteroxyz-mixed
           *  heteroU-mixed
           *  heteronoref-mixed
           Hydrogen treatment given as costr
PLAT028_ALERT_3_C _diffrn_measured_fraction_theta_max Value Low ..       0.939 Why?
PLAT094_ALERT_2_C Ratio of Maximum / Minimum Residual Density ....        2.58 Report
PLAT097_ALERT_2_C Large Reported Max.  (Positive) Residual Density        1.30 eA-3
PLAT242_ALERT_2_C Low    'MainMol' Ueq as Compared to Neighbors of          C1 Check
PLAT334_ALERT_2_C Small Aver. Benzene C-C Dist C6       -C7'              1.37 Ang.
PLAT340_ALERT_3_C Low Bond Precision on  C-C Bonds ...............     0.00487 Ang.
```

Alert level G

```
PLAT005_ALERT_5_G No Embedded Refinement Details Found  in the CIF    Please Do !
PLAT007_ALERT_5_G Number of Unrefined Donor-H Atoms ..............         1 Report
PLAT019_ALERT_1_G _diffrn_measured_fraction_theta_full/*_max < 1.0     0.838 Report
PLAT154_ALERT_1_G The s.u.'s on the Cell Angles are Equal ..(Note)     0.004 Degree
```

```
0 ALERT level A = Most likely a serious problem - resolve or explain
0 ALERT level B = A potentially serious problem, consider carefully
9 ALERT level C = Check. Ensure it is not caused by an omission or oversight
4 ALERT level G = General information/check it is not something unexpected

5 ALERT type 1 CIF construction/syntax error, inconsistent or missing data
4 ALERT type 2 Indicator that the structure model may be wrong or deficient
2 ALERT type 3 Indicator that the structure quality may be low
0 ALERT type 4 Improvement, methodology, query or suggestion
2 ALERT type 5 Informative message, check
```

checkCIF publication errors

Alert level A

```
PUBL006_ALERT_1_A  _publ_requested_journal is missing
           e.g. 'Acta Crystallographica Section C'
PUBL008_ALERT_1_A  _publ_section_title is missing. Title of paper.
PUBL009_ALERT_1_A  _publ_author_name is missing. List of author(s) name(s).
PUBL010_ALERT_1_A  _publ_author_address is missing. Author(s) address(es).
PUBL012_ALERT_1_A  _publ_section_abstract is missing.
           Abstract of paper in English.
```

```
  5 ALERT level A = Data missing that is essential or data in wrong format
  0 ALERT level G = General alerts. Data that may be required is missing
```

Publication of your CIF

You should attempt to resolve as many as possible of the alerts in all categories. Often the minor alerts point to easily fixed oversights, errors and omissions in your CIF or refinement strategy, so attention to these fine details can be worthwhile. In order to resolve some of the more serious problems it may be necessary to carry out additional measurements or structure refinements. However, the nature of your study may justify the reported deviations from journal submission requirements and the more serious of these should be commented upon in the discussion or experimental section of a paper or in the "special_details" fields of the CIF. *checkCIF* was carefully designed to identify outliers and unusual parameters, but every test has its limitations and alerts that are not important in a particular case may appear. Conversely, the absence of alerts does not guarantee there are no aspects of the results needing attention. It is up to the individual to critically assess their own results and, if necessary, seek expert advice.

If level A alerts remain, which you believe to be justified deviations, and you intend to submit this CIF for publication in a journal, you should additionally insert an explanation in your CIF using the Validation Reply Form (VRF) below. This will allow your explanation to be considered as part of the review process.

Validation response form

Please find below a validation response form (VRF) that can be filled in and pasted into your CIF.

```
# start Validation Reply Form
_vrf_PUBL006_GLOBAL
;
PROBLEM: _publ_requested_journal is missing
RESPONSE: ...
;
_vrf_PUBL008_GLOBAL
;
PROBLEM: _publ_section_title is missing. Title of paper.
RESPONSE: ...
;
_vrf_PUBL009_GLOBAL
;
PROBLEM: _publ_author_name is missing. List of author(s) name(s).
RESPONSE: ...
;
_vrf_PUBL010_GLOBAL
```

```
;
PROBLEM: _publ_author_address is missing. Author(s) address(es).
RESPONSE: ...
;
_vrf_PUBL012_GLOBAL
;
PROBLEM: _publ_section_abstract is missing.
RESPONSE: ...
;
# end Validation Reply Form
```

If you wish to submit your CIF for publication in Acta Crystallographica Section C or E, you should upload your CIF via the web. If you wish to submit your CIF for publication in IUCrData you should upload your CIF via the web. If your CIF is to form part of a submission to another IUCr journal, you will be asked, either during electronic submission or by the Co-editor handling your paper, to upload your CIF via our web site.

PLATON version of 09/11/2017; check.def file version of 08/11/2017

Datablock I - ellipsoid plot

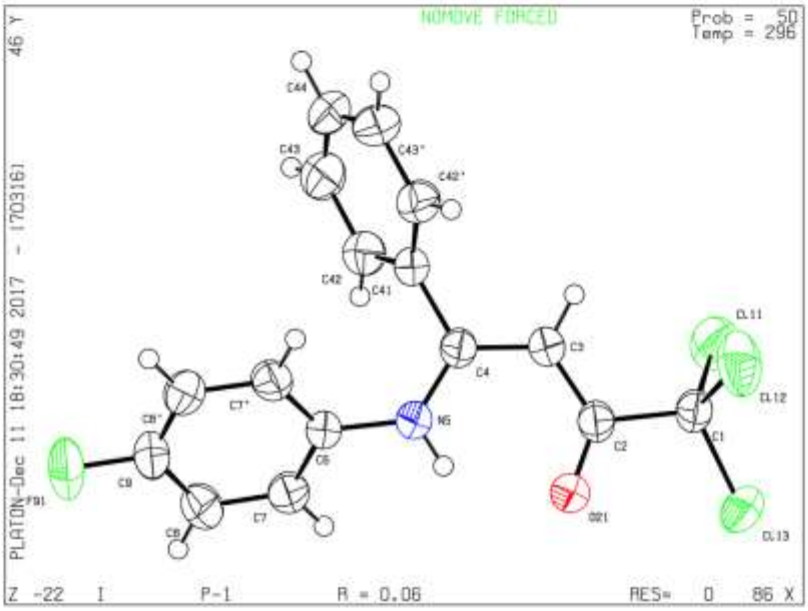

Single Crystal X-Ray Data of compound 6e.

checkCIF/PLATON report

You have not supplied any structure factors. As a result the full set of tests cannot be run.

THIS REPORT IS FOR GUIDANCE ONLY. IF USED AS PART OF A REVIEW PROCEDURE FOR PUBLICATION, IT SHOULD NOT REPLACE THE EXPERTISE OF AN EXPERIENCED CRYSTALLOGRAPHIC REFEREE.

No syntax errors found. CIF dictionary Interpreting this report

Datablock: I

Bond precision: C-C = 0.0033 A   Wavelength=1.54178

Cell:	a=5.7998(2)	b=16.0750(4)	c=19.7847(5)
	alpha=90	beta=90	gamma=90
Temperature:	293 K		

	Calculated	Reported
Volume	1844.56(9)	1844.56(9)
Space group	P 21 21 21	P212121
Hall group	P 2ac 2ab	P2ac2ab
Moiety formula	C17 H13 B Cl3 F2 N O2	C17 H13 B Cl3 F2 N O2
Sum formula	C17 H13 B Cl3 F2 N O2	C17 H13 B Cl3 F2 N O2
Mr	418.44	418.44
Dx,g cm-3	1.507	1.507
Z	4	4
Mu (mm-1)	4.787	4.787
F000	848.0	848.0
F000'	854.70	
h,k,lmax	6,19,23	6,19,23
Nref	3359[1964]	3232
Tmin,Tmax	0.352,0.614	0.558,0.753
Tmin'	0.040	

Correction method= # Reported T Limits: Tmin=0.558 Tmax=0.753
AbsCorr = GAUSSIAN

Data completeness= 1.65/0.96   Theta(max)= 68.080

R(reflections)= 0.0290(3154)   wR2(reflections)= 0.0784(3232)

S = 1.054   Npar= 235

The following ALERTS were generated. Each ALERT has the format
test-name_ALERT_alert-type_alert-level.
Click on the hyperlinks for more details of the test.

Alert level C

```
ABSTY02_ALERT_1_C  An _exptl_absorpt_correction_type has been given without
           a literature citation. This should be contained in the
           _exptl_absorpt_process_details field.
           Absorption correction given as gaussian
PLAT029_ALERT_3_C _diffrn_measured_fraction_theta_full value Low .      0.977 Note
PLAT241_ALERT_2_C High   'MainMol' Ueq as Compared to Neighbors of         O1 Check
PLAT242_ALERT_2_C Low    'MainMol' Ueq as Compared to Neighbors of         B2 Check
```

Alert level G

```
PLAT005_ALERT_5_G No Embedded Refinement Details found  in the CIF     Please Do !
PLAT033_ALERT_4_G Flack x Value Deviates > 3.0 * sigma from Zero .      0.056 Note
PLAT063_ALERT_4_G Crystal Size Likely too Large for Beam Size ....       0.64 mm
PLAT199_ALERT_1_G Reported _cell_measurement_temperature ..... (K)        293 Check
PLAT200_ALERT_1_G Reported   _diffrn_ambient_temperature ..... (K)        293 Check
PLAT395_ALERT_2_G Deviating  X-O-Y  Angle from 120 Deg for  O1          123.3 Degree
```

0 **ALERT level A** = Most likely a serious problem - resolve or explain
0 **ALERT level B** = A potentially serious problem, consider carefully
4 **ALERT level C** = Check. Ensure it is not caused by an omission or oversight
6 **ALERT level G** = General information/check it is not something unexpected

3 ALERT type 1 CIF construction/syntax error, inconsistent or missing data
3 ALERT type 2 Indicator that the structure model may be wrong or deficient
1 ALERT type 3 Indicator that the structure quality may be low
2 ALERT type 4 Improvement, methodology, query or suggestion
1 ALERT type 5 Informative message, check

checkCIF publication errors

Alert level A

```
PUBL006_ALERT_1_A   _publ_requested_journal is missing
           e.g. 'Acta Crystallographica Section C'
PUBL008_ALERT_1_A   _publ_section_title is missing. Title of paper.
PUBL009_ALERT_1_A   _publ_author_name is missing. List of author(s) name(s).
PUBL010_ALERT_1_A   _publ_author_address is missing. Author(s) address(es).
PUBL012_ALERT_1_A   _publ_section_abstract is missing.
           Abstract of paper in English.
```

5 **ALERT level A** = Data missing that is essential or data in wrong format
0 **ALERT level G** = General alerts. Data that may be required is missing

Publication of your CIF

You should attempt to resolve as many as possible of the alerts in all categories. Often the minor alerts point to easily fixed oversights, errors and omissions in your CIF or refinement strategy, so attention to these fine details can be worthwhile. In order to resolve some of the more serious problems it may be necessary to carry out additional measurements or structure refinements. However, the nature of your study may justify the reported deviations from journal submission requirements and the more serious of these should be commented upon in the discussion or experimental section of a paper or in the "special_details" fields of the CIF. *checkCIF* was carefully designed to identify outliers and unusual parameters, but every test has its limitations and alerts that are not important in a particular case may appear. Conversely, the absence of alerts does not guarantee there are no aspects of the results needing attention. It is up to the individual to critically assess their own results and, if necessary, seek expert advice.

If level A alerts remain, which you believe to be justified deviations, and you intend to submit this CIF for publication in a journal, you should additionally insert an explanation in your CIF using the Validation Reply Form (VRF) below. This will allow your explanation to be considered as part of the review process.

Validation response form

Please find below a validation response form (VRF) that can be filled in and pasted into your CIF.

```
# start Validation Reply Form
_vrf_PUBL006_GLOBAL
;
PROBLEM: _publ_requested_journal is missing
RESPONSE: ...
;
_vrf_PUBL008_GLOBAL
;
PROBLEM: _publ_section_title is missing. Title of paper.
RESPONSE: ...
;
_vrf_PUBL009_GLOBAL
;
PROBLEM: _publ_author_name is missing. List of author(s) name(s).
RESPONSE: ...
;
_vrf_PUBL010_GLOBAL
;
PROBLEM: _publ_author_address is missing. Author(s) address(es).
RESPONSE: ...
;
_vrf_PUBL012_GLOBAL
;
PROBLEM: _publ_section_abstract is missing.
RESPONSE: ...
;
# end Validation Reply Form
```

If you wish to submit your CIF for publication in Acta Crystallographica Section C or E, you should upload your CIF via the web. If you wish to submit your CIF for publication in IUCrData you should upload your CIF via the web. If your CIF is to form part of a submission to another IUCr journal, you will be asked, either during electronic submission or by the Co-editor handling your paper, to upload your CIF via our web site.

PLATON version of 13/08/2017; check.def file version of 27/07/2017

Datablock I - ellipsoid plot

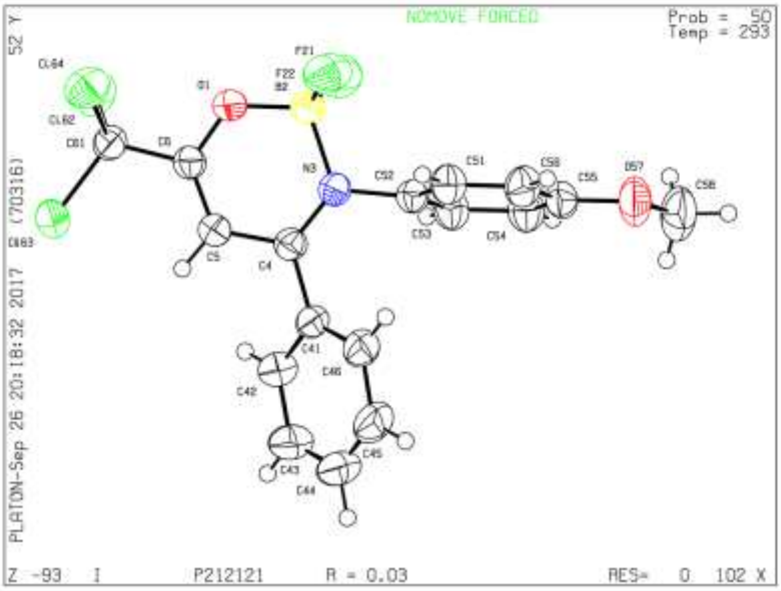

4. UV-VIS Spectra for compounds 6a-i and 7e.

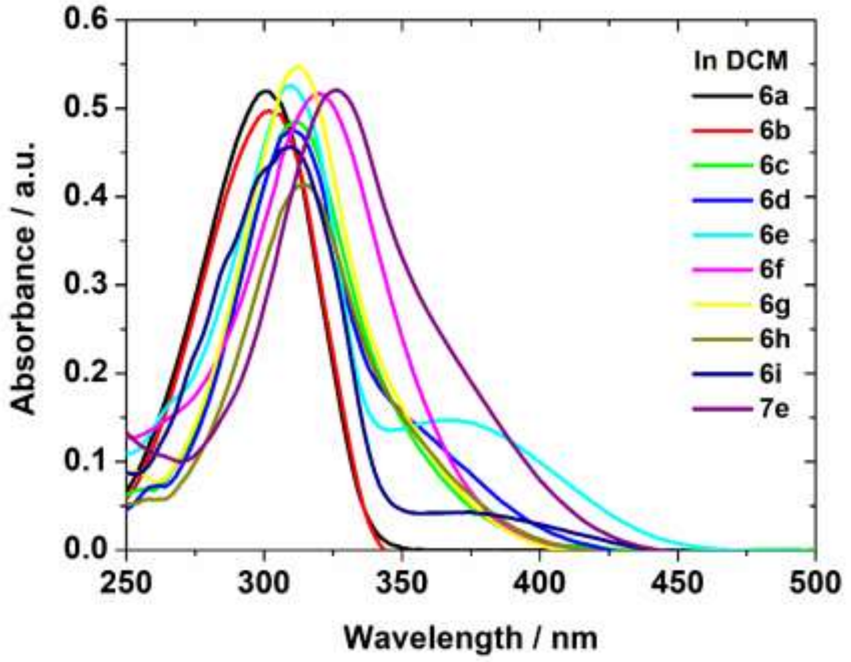

Figure S35. UV-Vis absorption spectra in DCM solution of boron complexes **6a-i** and **7e**.

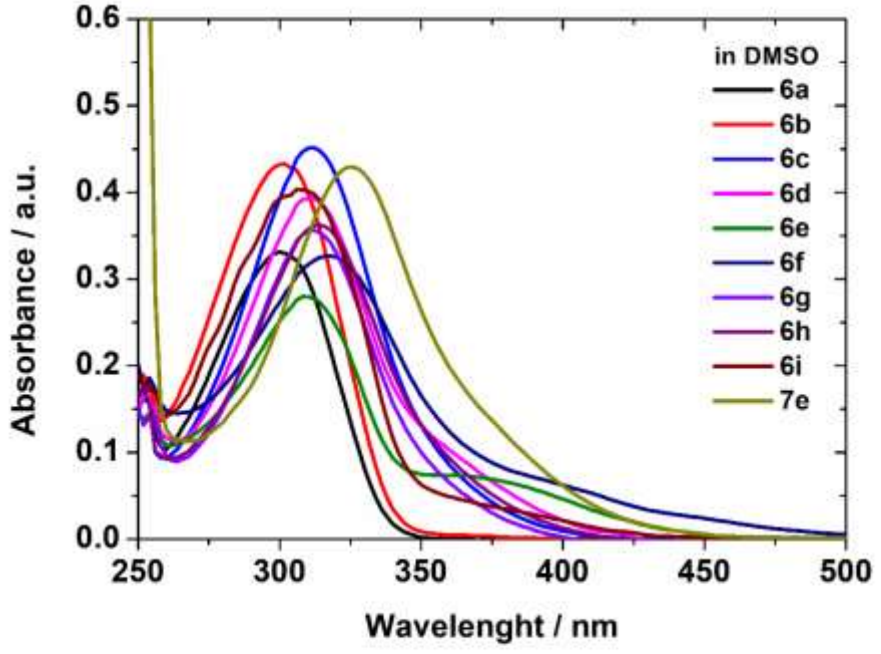

Figure S36. UV-Vis absorption spectra in DMSO solution of boron complexes **6a-i** and **7e**.

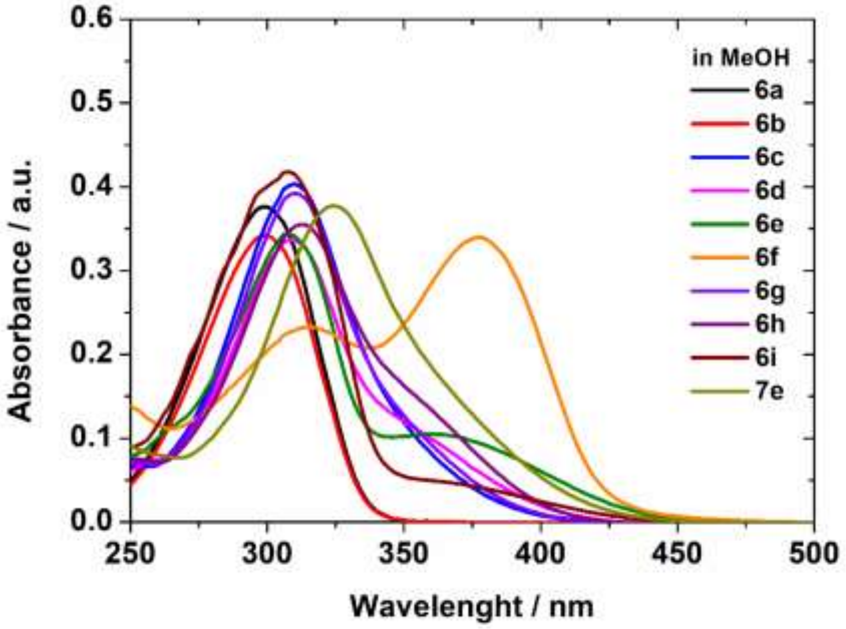

Figure S37. UV-Vis absorption spectra in MeOH solution of boron complexes **6a-i** and **7e**.

5. Steady-state emission fluorescence spectra

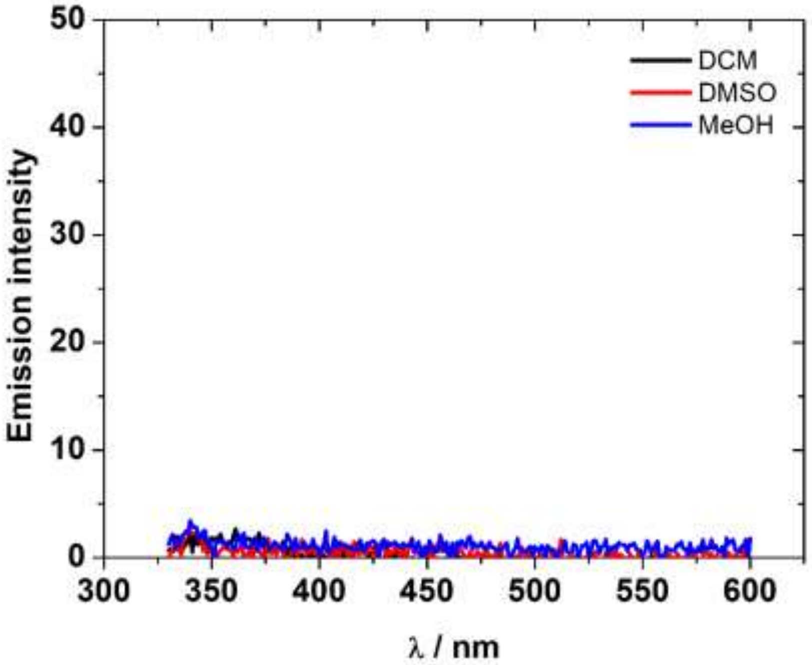

Figure S38. Comparative steady-state emission fluorescence spectra in solution of boron complex **6a**.

6. UV-VIS DNA titrations spectra.

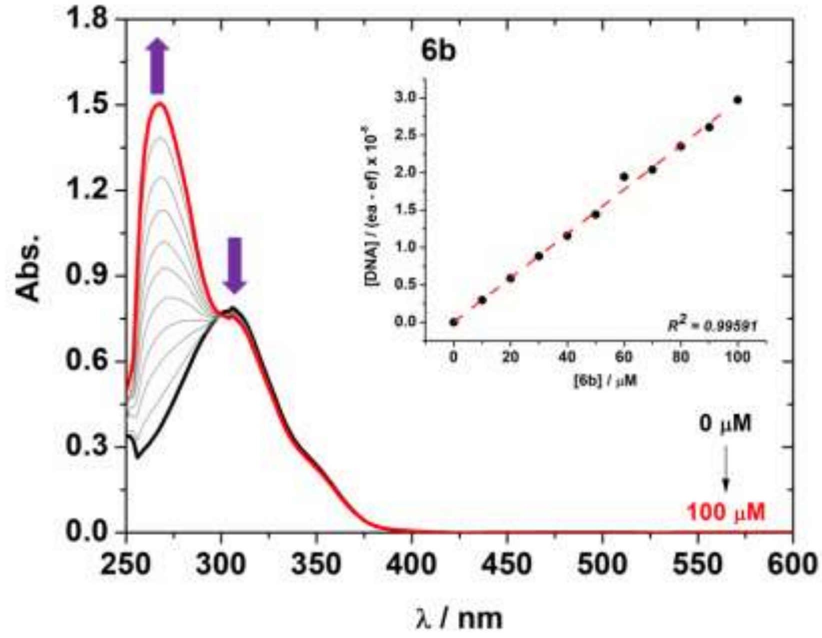

Figure S39. UV–Vis absorption spectra for compound **6b** and the effect of successive additions of CT-DNA solution in the presence of a fixed concentration of **6b**, in a DMSO(2%)/Tris-HCl buffer mixture (pH = 7.2). Insert graph shows the plot [DNA]/($\varepsilon_a - \varepsilon_f$) *versus* [DNA]. The concentration of CT-DNA ranged from 0 to 100 μM.

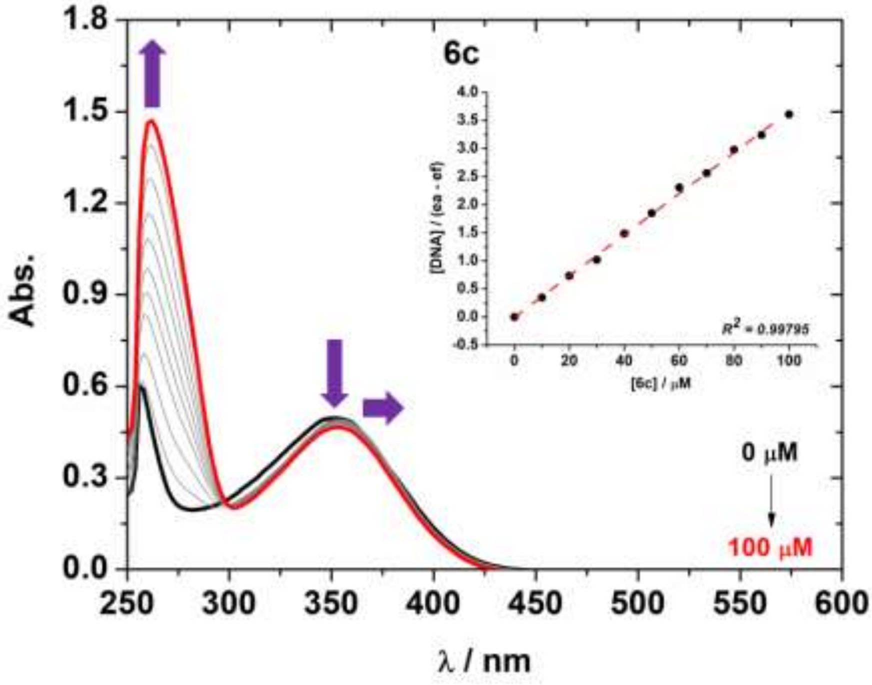

Figure S40. UV–Vis absorption spectra for compound **6c** and the effect of successive additions of CT-DNA solution in the presence of a fixed concentration of **6c**, in a DMSO(2%)/Tris-HCl buffer mixture (pH = 7.2). Insert graph shows the plot [DNA]/($\varepsilon_a - \varepsilon_f$) *versus* [DNA]. The concentration of CT-DNA ranged from 0 to 100 μM.

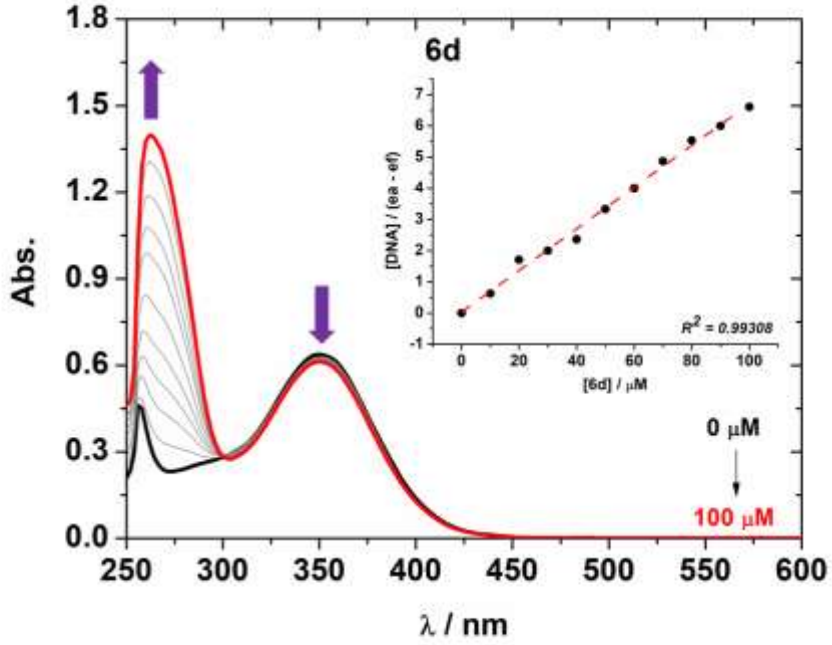

Figure S41. UV–Vis absorption spectra for compound **6d** and the effect of successive additions of CT-DNA solution in the presence of a fixed concentration of **6d**, in a DMSO(2%)/Tris-HCl buffer mixture (pH = 7.2). Insert graph shows the plot [DNA]/(ε_a – ε_f) *versus* [DNA]. The concentration of CT-DNA ranged from 0 to 100 µM.

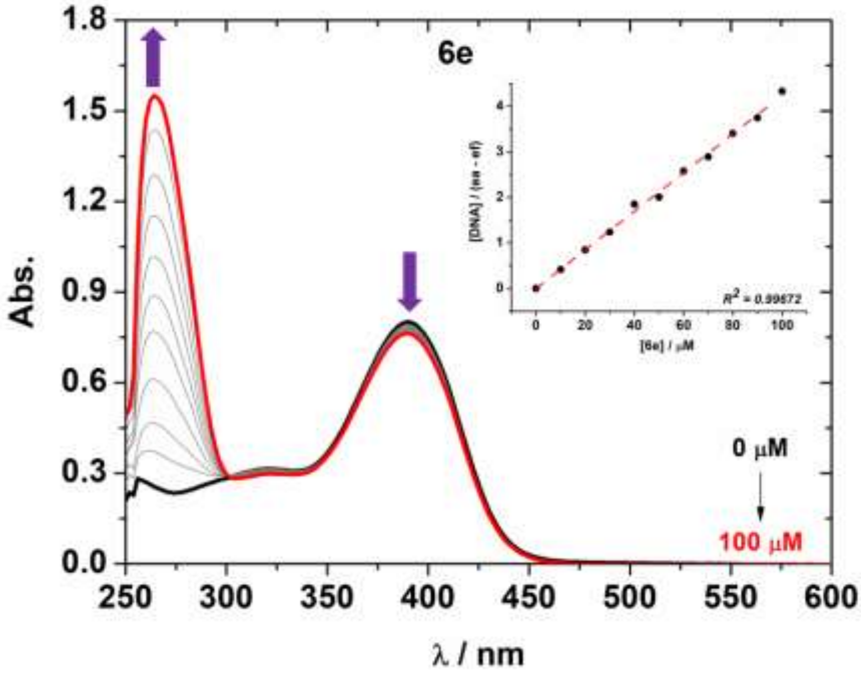

Figure S42. UV–Vis absorption spectra for compound **6e** and the effect of successive additions of CT-DNA solution in the presence of a fixed concentration of **6e**, in a DMSO(2%)/Tris-HCl buffer mixture (pH = 7.2). Insert graph shows the plot [DNA]/(ε_a – ε_f) *versus* [DNA]. The concentration of CT-DNA ranged from 0 to 100 µM.

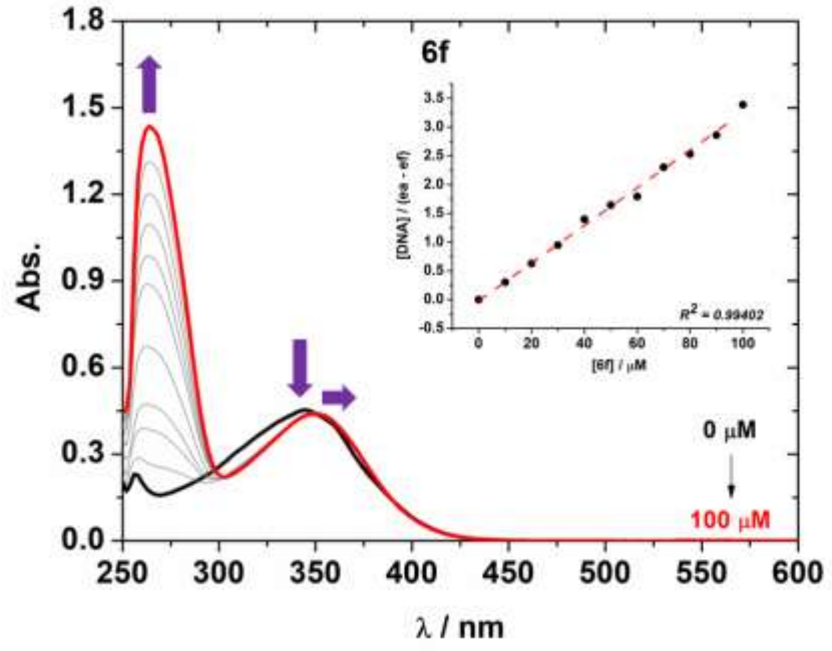

Figure S43. UV–Vis absorption spectra for compound **6f** and the effect of successive additions of CT-DNA solution in the presence of a fixed concentration of **6f**, in a DMSO(2%)/Tris-HCl buffer mixture (pH = 7.2). Insert graph shows the plot [DNA]/(ε_a – ε_f) *versus* [DNA]. The concentration of CT-DNA ranged from 0 to 100 μM.

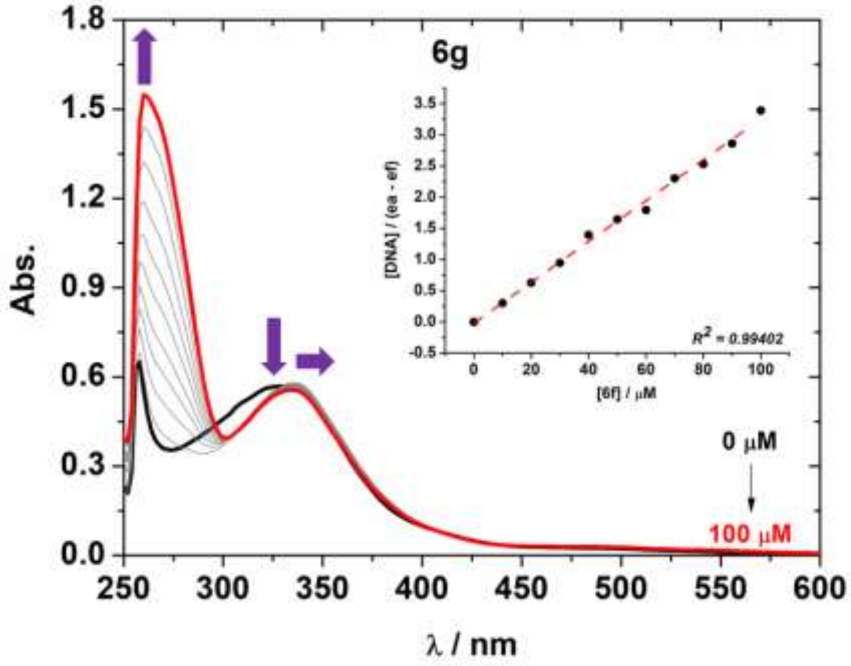

Figure S44. UV–Vis absorption spectra for compound **6g** and the effect of successive additions of CT-DNA solution in the presence of a fixed concentration of **6g**, in a DMSO(2%)/Tris-HCl buffer mixture (pH = 7.2). Insert graph shows the plot [DNA]/(ε_a – ε_f) *versus* [DNA]. The concentration of CT-DNA ranged from 0 to 100 μM.

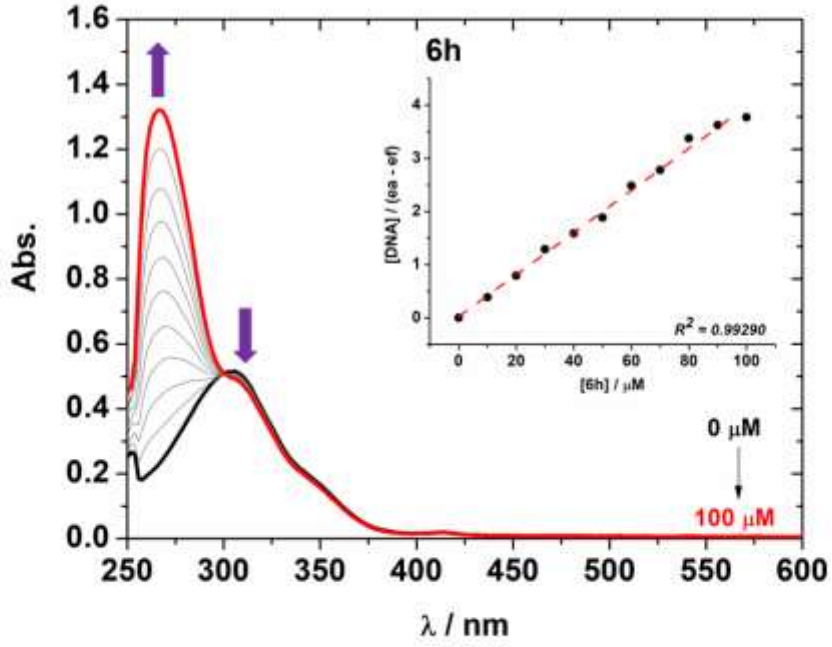

Figure S45. UV–Vis absorption spectra for compound **6h** and the effect of successive additions of CT-DNA solution in the presence of a fixed concentration of **6h**, in a DMSO(2%)/Tris-HCl buffer mixture (pH = 7.2). Insert graph shows the plot [DNA]/(ε_a – ε_f) *versus* [DNA]. The concentration of CT-DNA ranged from 0 to 100 μM.

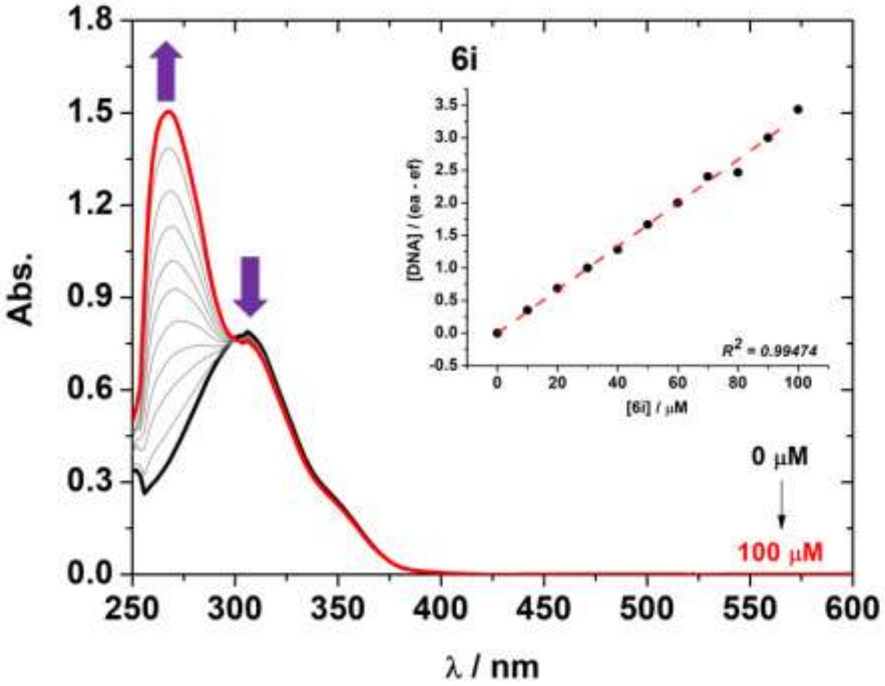

Figure S46. UV–Vis absorption spectra for compound **6i** and the effect of successive additions of CT-DNA solution in the presence of a fixed concentration of **6i**, in a DMSO(2%)/Tris-HCl buffer mixture (pH = 7.2). Insert graph shows the plot [DNA]/(ε_a – ε_f) *versus* [DNA]. The concentration of CT-DNA ranged from 0 to 100 μM.

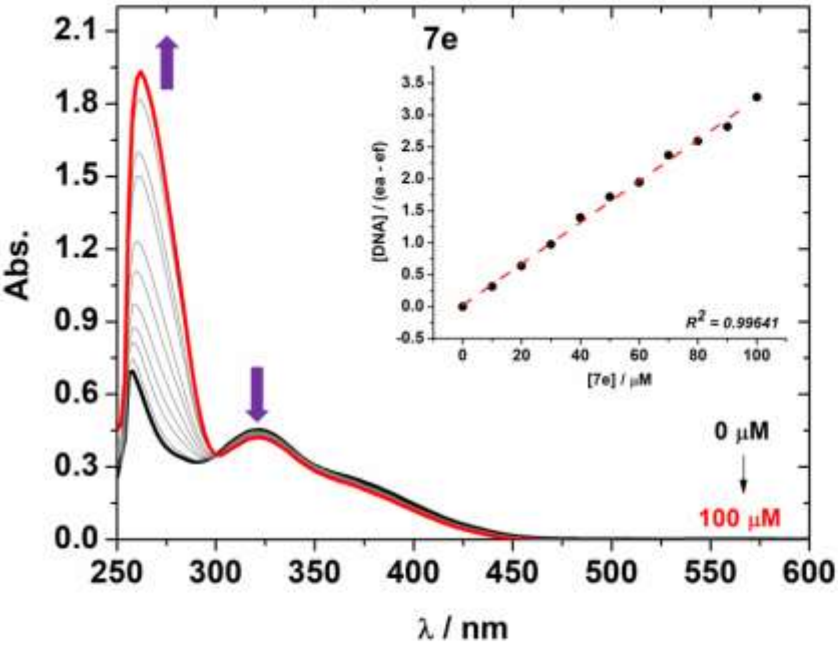

Figure S47. UV–Vis absorption spectra for compound **7e** and the effect of successive additions of CT-DNA solution in the presence of a fixed concentration of **7e**, in a DMSO(2%)/Tris-HCl buffer mixture (pH = 7.2). Insert graph shows the plot [DNA]/($\varepsilon_a - \varepsilon_f$) *versus* [DNA]. The concentration of CT-DNA ranged from 0 to 100 μM.

7. Competitive EB-DNA assays by emission fluorescence spectra

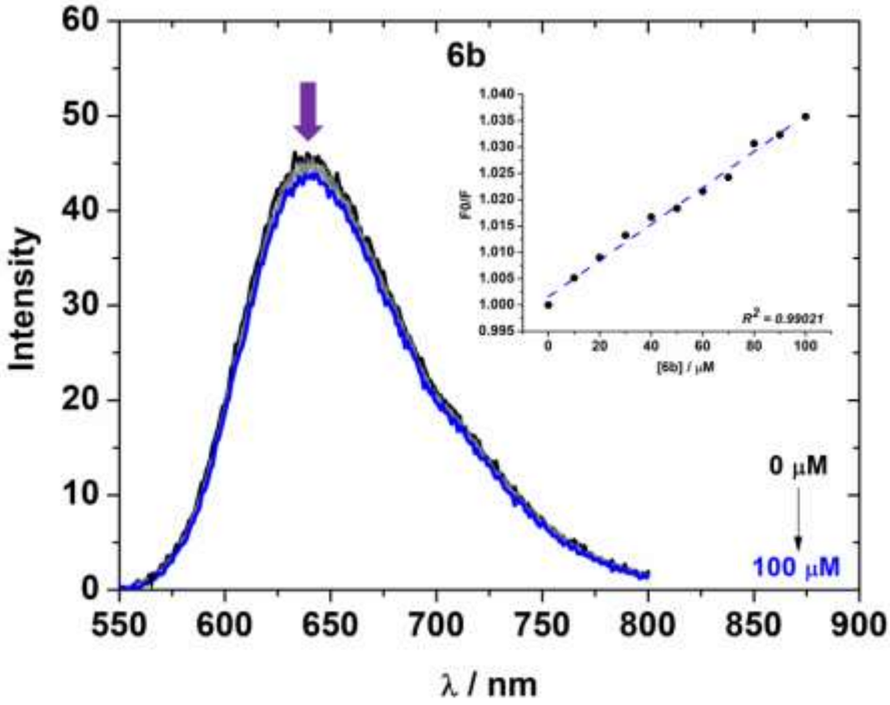

Figure S48. Fluorescence emission spectra of EB bound to CT-DNA in the presence of **6b** in a DMSO(2%)/Tris-HCl pH 7.2 mixture at λ_{exc}=510 nm. The inset shows the plot of F_0/F *versus* the concentration of compound **6b** according to the Stern-Volmer equation.

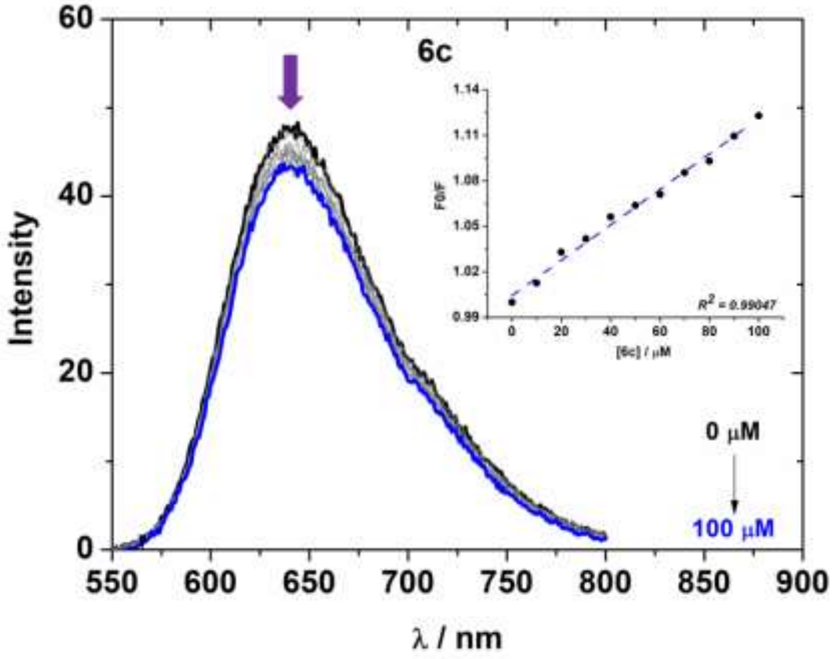

Figure S49. Fluorescence emission spectra of EB bound to CT-DNA in the presence of **6c** in a DMSO(2%)/Tris-HCl pH 7.2 mixture at λ_{exc}=510 nm. The inset shows the plot of F_0/F *versus* the concentration of compound **6c** according to the Stern-Volmer equation.

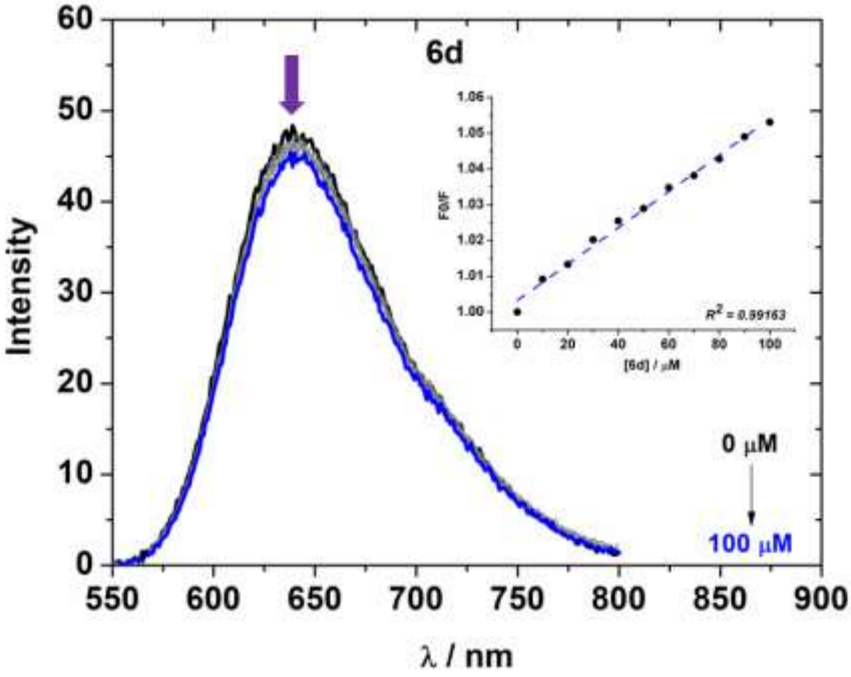

Figure S50. Fluorescence emission spectra of EB bound to CT-DNA in the presence of **6d** in a DMSO(2%)/Tris-HCl pH 7.2 mixture at λ_{exc}=510 nm. The inset shows the plot of F_0/F *versus* the concentration of compound **6d** according to the Stern-Volmer equation.

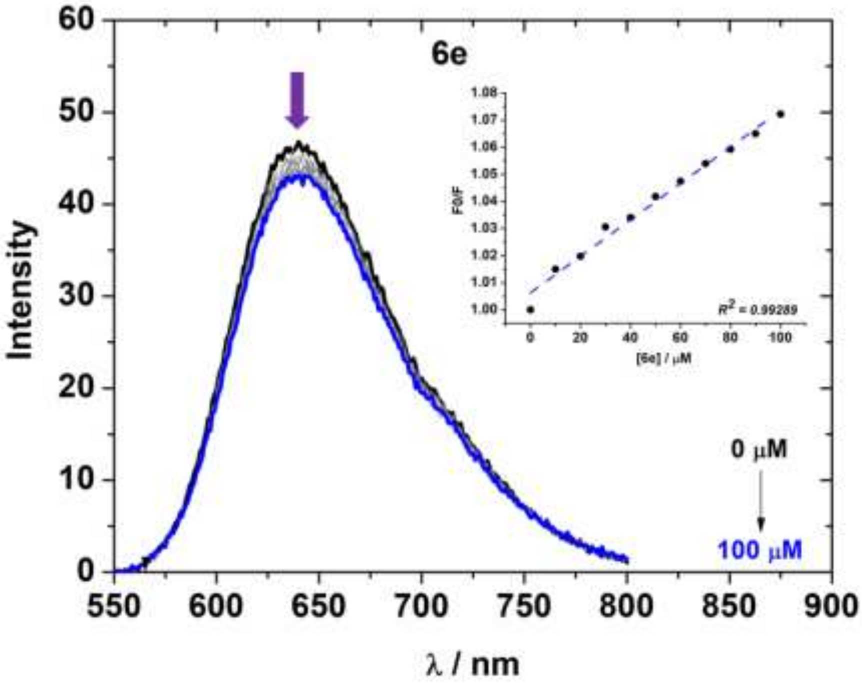

Figure S51. Fluorescence emission spectra of EB bound to CT-DNA in the presence of **6e** in a DMSO(2%)/Tris-HCl pH 7.2 mixture at λ_{exc}=510 nm. The inset shows the plot of F_0/F *versus* the concentration of compound **6e** according to the Stern-Volmer equation.

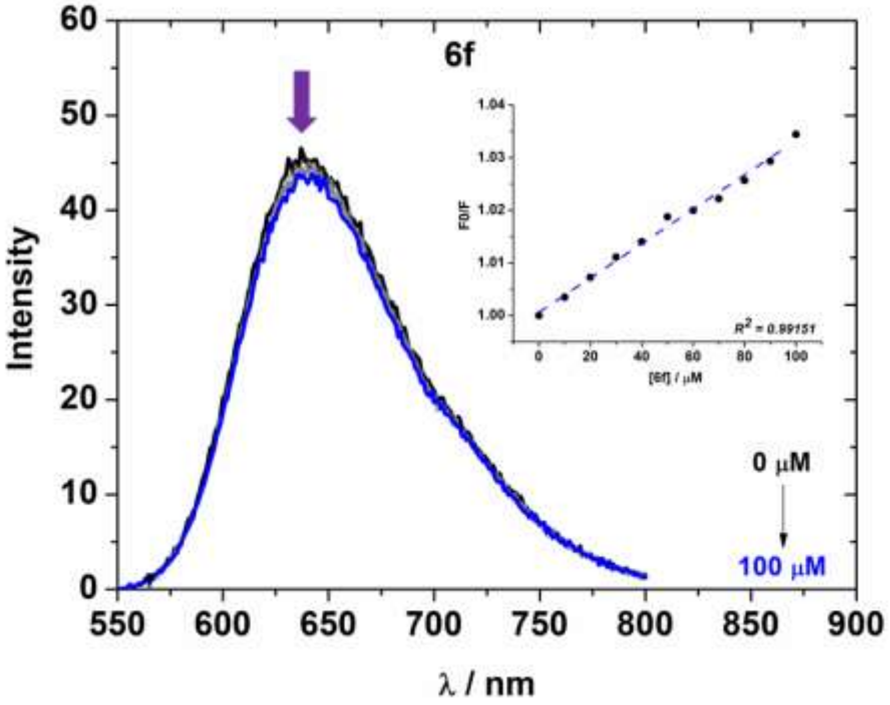

Figure S52. Fluorescence emission spectra of EB bound to CT-DNA in the presence of **6f** in a DMSO(2%)/Tris-HCl pH 7.2 mixture at λ_{exc}=510 nm. The inset shows the plot of F_0/F *versus* the concentration of compound **6f** according to the Stern-Volmer equation.

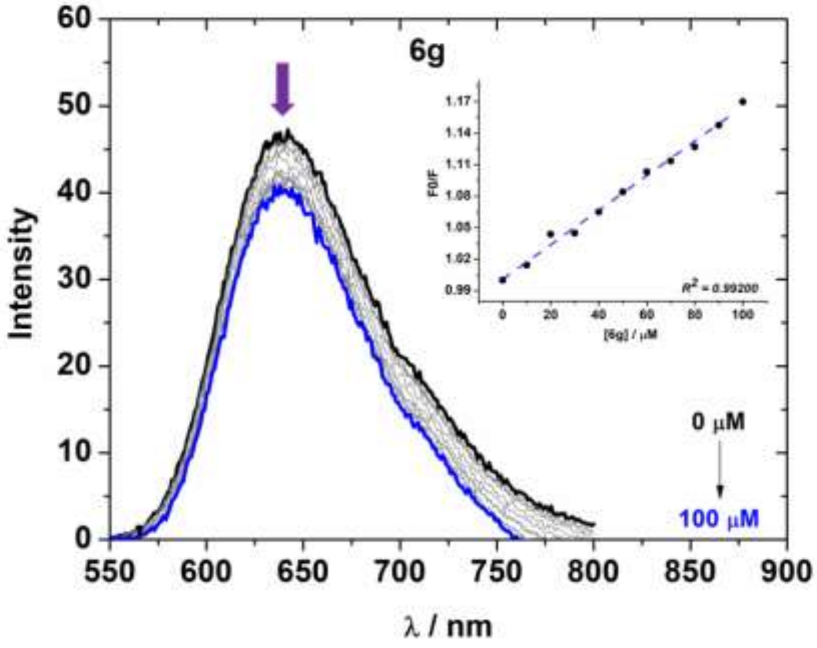

Figure S53. Fluorescence emission spectra of EB bound to CT-DNA in the presence of **6g** in a DMSO(2%)/Tris-HCl pH 7.2 mixture at λ_{exc}=510 nm. The inset shows the plot of F_0/F *versus* the concentration of compound **6g** according to the Stern-Volmer equation.

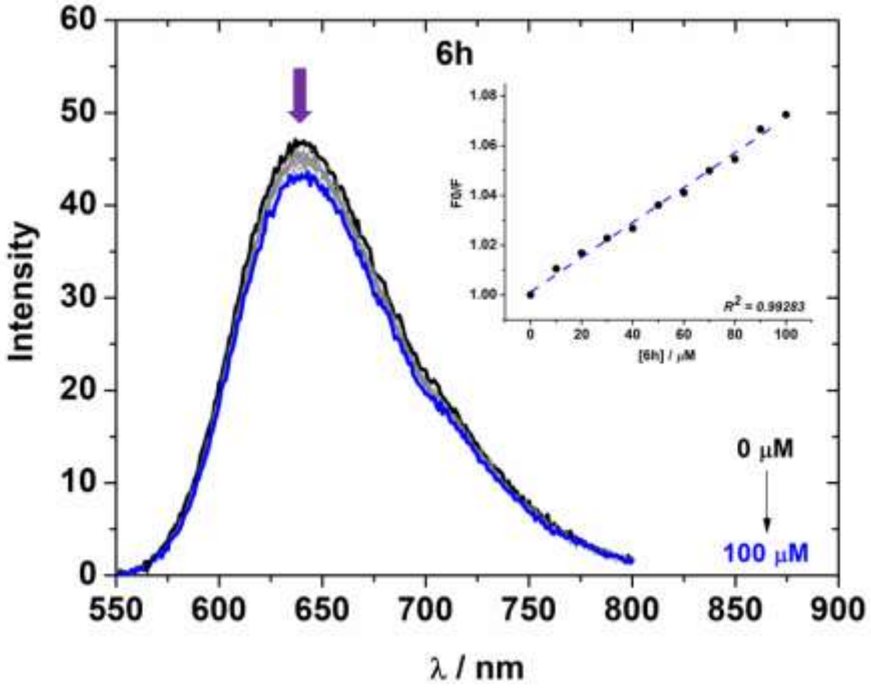

Figure S54. Fluorescence emission spectra of EB bound to CT-DNA in the presence of **6h** in a DMSO(2%)/Tris-HCl pH 7.2 mixture at λ_{exc}=510 nm. The inset shows the plot of F_0/F *versus* the concentration of compound **6h** according to the Stern-Volmer equation.

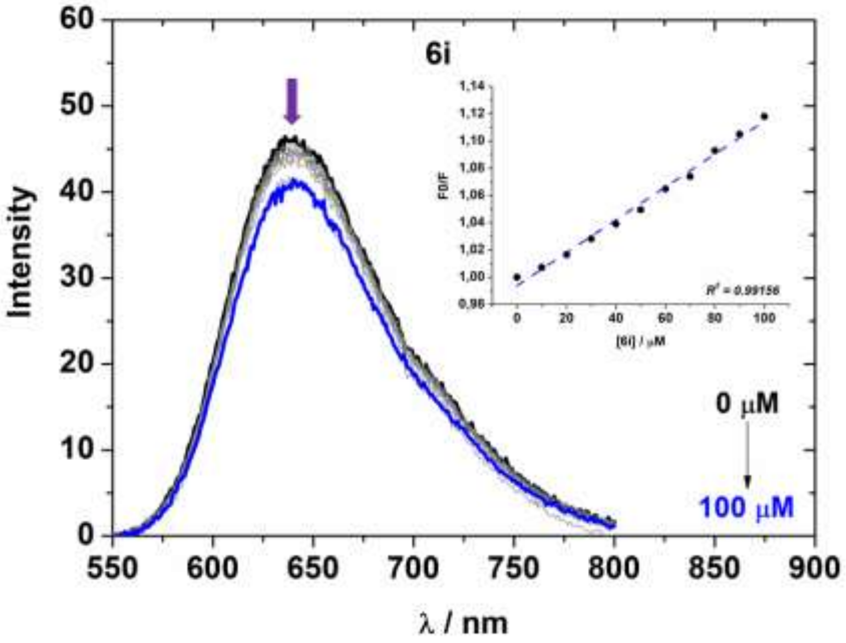

Figure S55. Fluorescence emission spectra of EB bound to CT-DNA in the presence of **6i** in a DMSO(2%)/Tris-HCl pH 7.2 mixture at λ_{exc}=510 nm. The inset shows the plot of F_0/F *versus* the concentration of compound **6i** according to the Stern-Volmer equation.

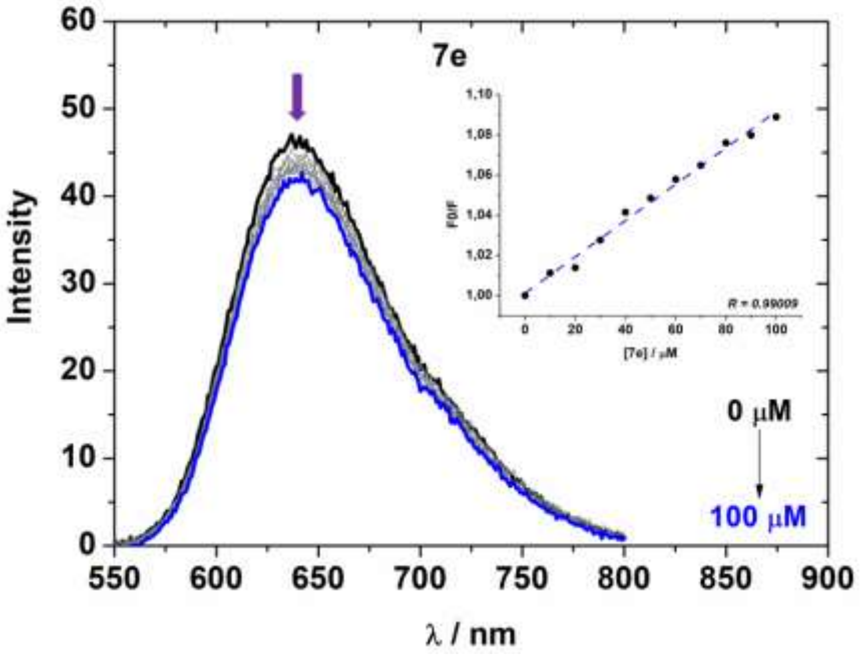

Figure S56. Fluorescence emission spectra of EB bound to CT-DNA in the presence of **7e** in a DMSO (2%)/Tris-HCl pH 7.2 mixture at λ_{exc}=510 nm. The inset shows the plot of F_0/F *versus* the concentration of compound **7e** according to the Stern-Volmer equation.

8. TD-DFT Calculations

All calculations were done using the Gaussian 09 package of program. All geometrical structures were optimized at the SCRF(PCM)-B3LYP/cc-pVTZ level of theory, with single point energies and molecular orbitals calculated at the same level of theory. The PCM model was used to account for the solvent effect. Harmonic frequency calculations were done in order to confirm that the geometries were at the minimum potential energy

Table S3. Excitation energy (E), wavelength of maximum absorbance (λ_{max}), and oscillator strengths (f) for HOMO-LUMO orbitals in CH_2Cl_2, DMSO and MeOH for compound **6a**. Calculated at the TD-DFT (SCRF(PCM))-B3LYP/cc-pVTZ level.

Solvente	Eletronic Transitions	Energy (eV)	λmax (nm)	Main Orbital Transition	*F*
CH_2Cl_2	$S_0 \rightarrow S_1$	4.0338	307.36	88-> 91 0.19059<br>89-> 91 0.59301<br>90-> 91 0.32649	0.1034
	$S_0 \rightarrow S_2$	4.1314	300.10	88-> 91 0.35302<br>89->91 -0.37669<br>90->91 0.48004	0.1260
	$S_0 \rightarrow S_3$	4.3134	287.44	88->91 0.57808<br>90->91 -0.39955	0.2549
DMSO	$S_0 \rightarrow S_1$	3.9833	311.26	88->91 0.18891<br>89->91 0.60689<br>90->91 0.30081	0.0806
	$S_0 \rightarrow S_2$	4.0828	303.67	88->91 0.38352<br>89->91 -0.35386<br>90->91 0.47411	0.0942
	$S_0 \rightarrow S_3$	4.3023	288.18	88->91 0.55882<br>90 ->91 -0.42599	0.3121
MeOH	$S_0 \rightarrow S_1$	3.9906	310.69	88->91 0.18796<br>89->91 0.61152<br>90->91 0.29179	0.0754
	$S_0 \rightarrow S_2$	4.0906	303.10	88->91 0.39485<br>89->91 -0.34559<br>90->91 0.47087	0.0914
	$S_0 \rightarrow S_3$	4.3110	287.60	88->91 0.55117<br>90->91 0.43574	0.3078

Figure S57. Calculated UV-Vis spectra for compound **6a** in DCM, DMSO and MeOH.

CH_2Cl_2

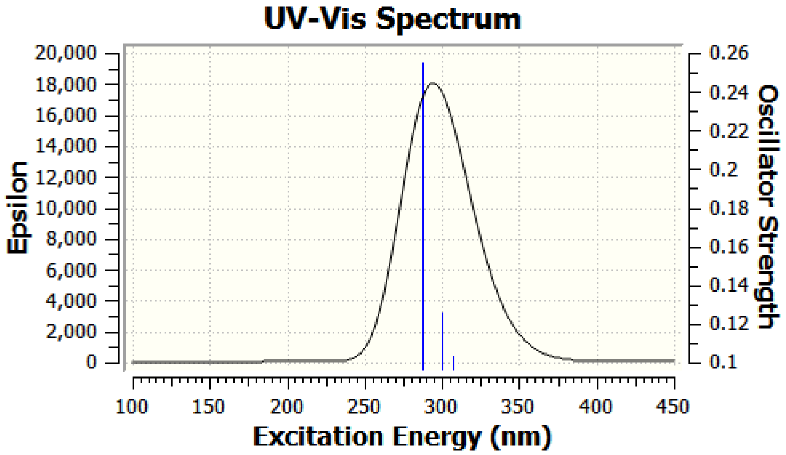

DMSO

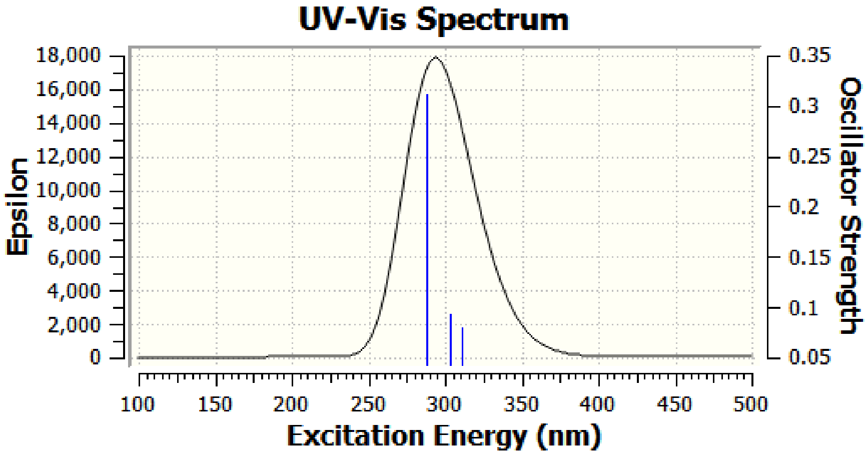

MeOH

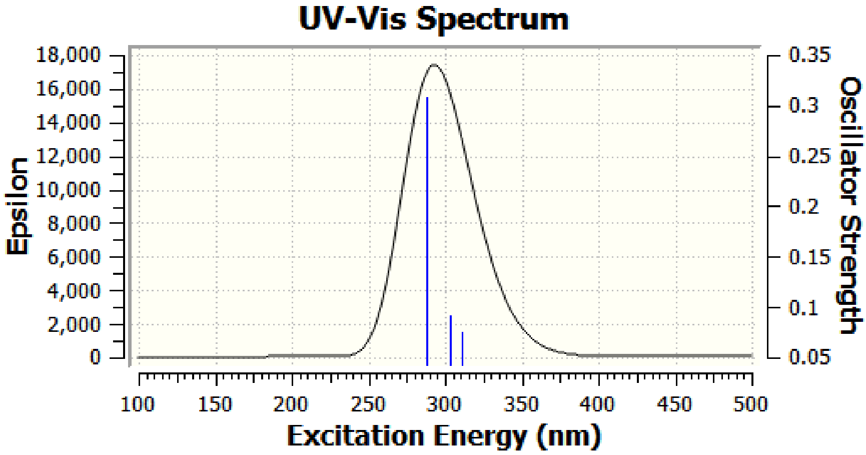

Figure S58.

CH_2Cl_2

HOMO LUMO

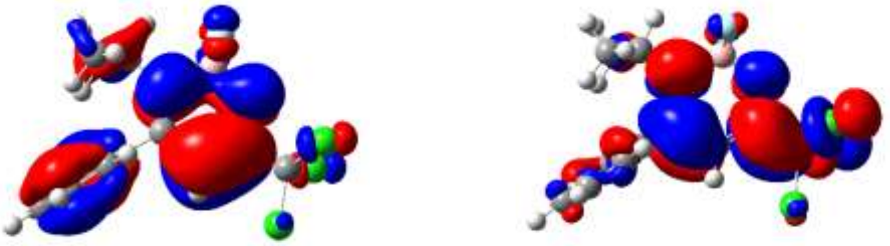

Figure S59.

DMSO

HOMO LUMO

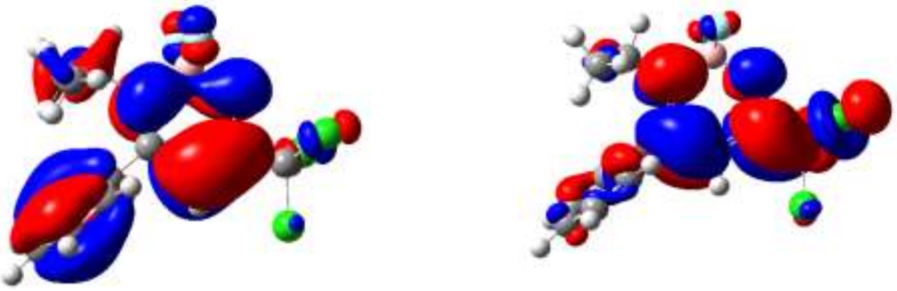

Figure S60.

MeOH

HOMO LUMO

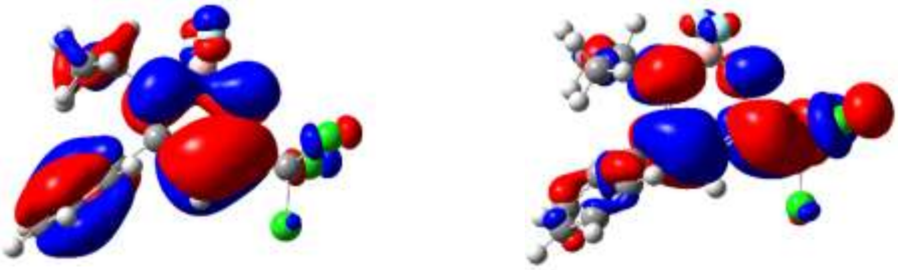

Table S4. Excitation energy (E), wavelength of maximum absorbance (λ_{max}), and oscillator strengths (*f*) for HOMO-LUMO orbitals in CH_2Cl_2, DMSO and MeOH for compound **6b**. Calculated at the TD-DFT (SCRF(PCM))-B3LYP/cc-pVTZ level.

Solvente	Eletronic Transitions	Energy (eV)	λmax (nm)	Main Orbital Transition	*F*
$CHCl_2$	$S_0 \rightarrow S_1$	4.0823	303.71	92 -> 95 -0.12387<br>93 -> 95 0.66122<br>94 -> 95 -0.20431	0.1641
	$S_0 \rightarrow S_2$	4.1422	299.32	92 -> 95 0.60112<br>94 -> 95 -0.36527	0.0607
	$S_0 \rightarrow S_3$	4.2024	295.03	92 -> 95 0.34355<br>93 -> 95 0.23748<br>94 -> 95 0.56656	0.3021
DMSO	$S_0 \rightarrow S_1$	4.0318	307.51	92 -> 95 -0.16215<br>93 -> 95 0.66880<br>94 -> 95 -0.14379	0.1332
	$S_0 \rightarrow S_2$	4.0857	303.46	92 -> 95 0.64716<br>93 -> 95 0.10211<br>94 -> 95 -0.25634	0.0175
	$S_0 \rightarrow S_3$	4.1981	295.34	92 -> 95 0.22337<br>93 -> 95 0.19010<br>94 -> 95 0.64012	0.3808
MeOH	$S_0 \rightarrow S_1$	4.0404	306.86	92 -> 95 -0.15707<br>93 -> 95 0.67160<br>94 -> 95 -0.13561	0.1246
	$S_0 \rightarrow S_2$	4.0930	302.92	92 -> 95 0.64880<br>93 -> 95 0.10109<br>94 -> 95 -0.25242	0.0169
	$S_0 \rightarrow S_3$	4.2072	294.69	92 -> 95 0.22208<br>93 -> 95 0.18016<br>94 -> 95 0.64343	0.3763

Figure S61. Calculated UV-Vis spectra for compound **6b** in DCM, DMSO and MeOH.

CH_2Cl_2

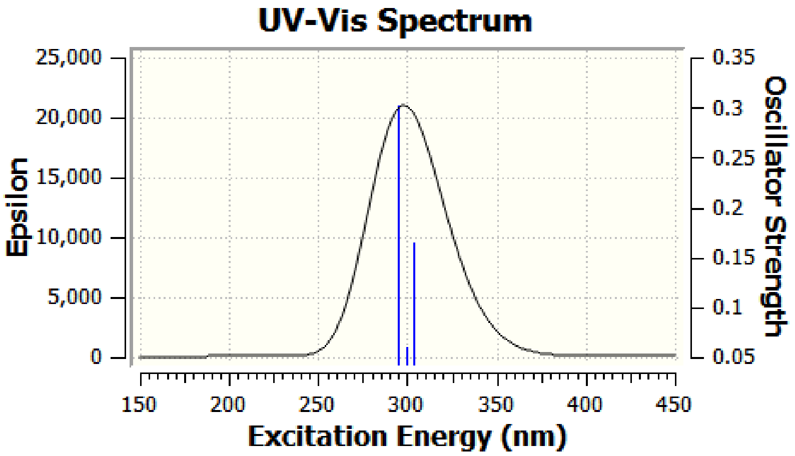

DMSO

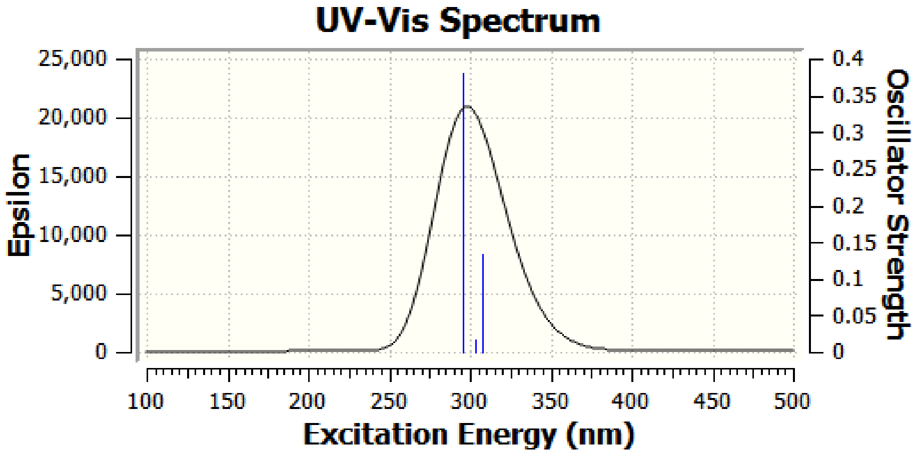

MeOH

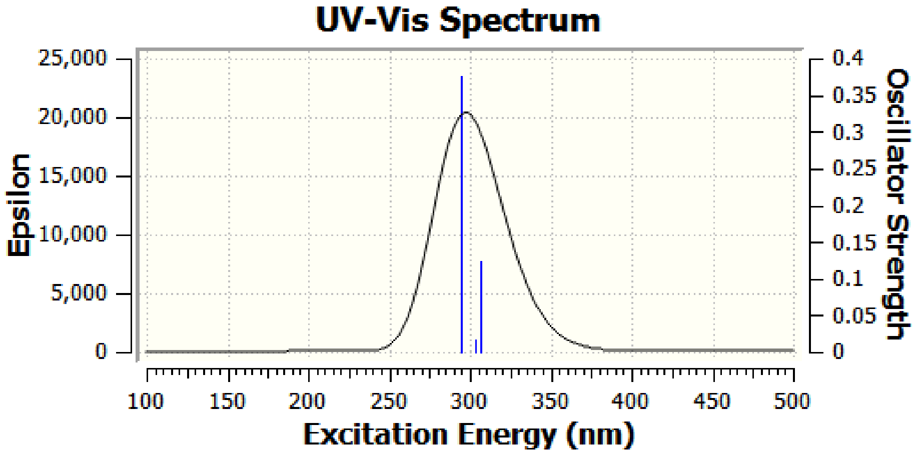

Figure S62.

CH_2Cl_2

HOMO LUMO

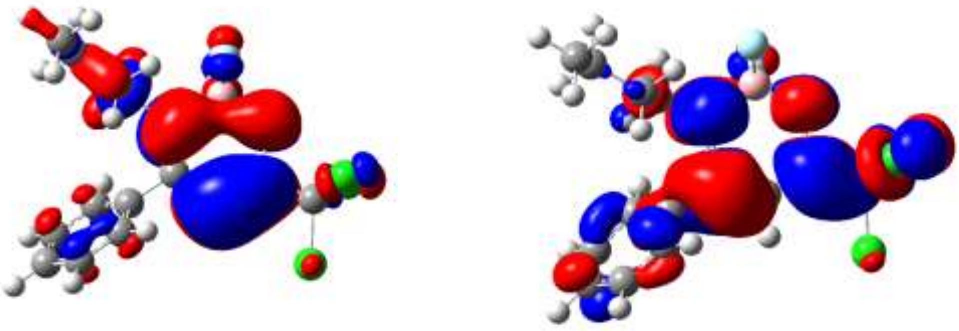

Figure S63.

DMSO

HOMO LUMO

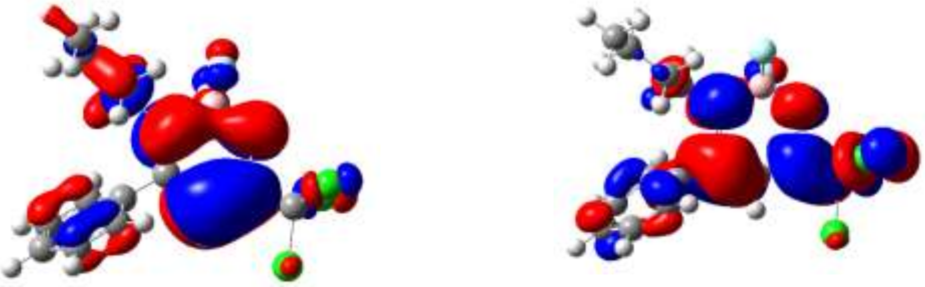

Figure S64.

MeOH

HOMO LUMO

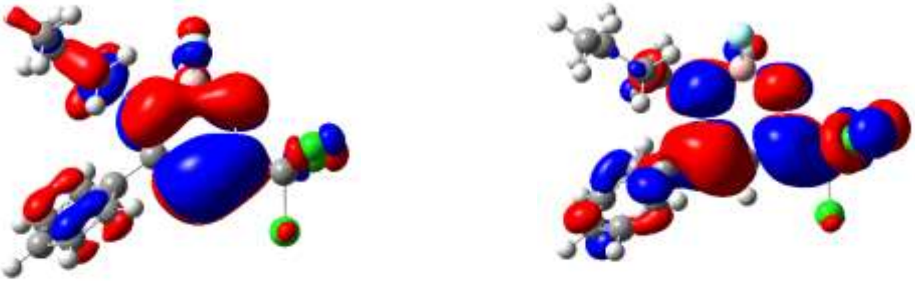

Table S5. Excitation energy (E), wavelength of maximum absorbance (λ_{max}), and oscillator strengths (*f*) for HOMO-LUMO orbitals in CH_2Cl_2, DMSO and MeOH for compound **6c**. Calculated at the TD-DFT (SCRF(PCM))-B3LYP/cc-pVTZ level.

Solvente	Eletronic Transitions	Energy (eV)	λmax (nm)	Main Orbital Transition	*F*
CH_2Cl_2	$S_0 \rightarrow S_1$	3.4565	358.70	98 -> 99 0.69241	0.1148
	$S_0 \rightarrow S_2$	3.8209	324.49	96 -> 99 0.23182<br>97 -> 99 0.66346	0.0752
	$S_0 \rightarrow S_3$	3.9770	311.75	95 -> 99 0.60326<br>96 -> 99 0.33090<br>97 -> 99 -0.10859	0.0506
DMSO	$S_0 \rightarrow S_1$	3.4611	358.22	98 -> 99 0.69353	0.1023
	$S_0 \rightarrow S_2$	3.8225	324.36	96 -> 99 -<br>0. 26777<br>97 -> 99<br>0. 64997	0.0792
	$S_0 \rightarrow S_3$	3.9366	314.95	95 -> 99 0.60022<br>96 -> 99 0.33641<br>97 -> 99 0.12037	0.0317
MeOH	$S_0 \rightarrow S_1$	3.4639	357.93	98 -> 99 0.69299	0.0995
	$S_0 \rightarrow S_2$	3.8241	324.22	96 -> 99 -0.27534<br>97 -> 99 0.64688	0.0693
	$S_0 \rightarrow S_3$	3.9423	314.50	95 -> 99 0.61191<br>96 -> 99 0.31674<br>97 -> 99 0.11425	0.0284

Figure S64. Calculated UV-Vis spectra for compound **6c** in DCM, DMSO and MeOH.

CH_2Cl_2

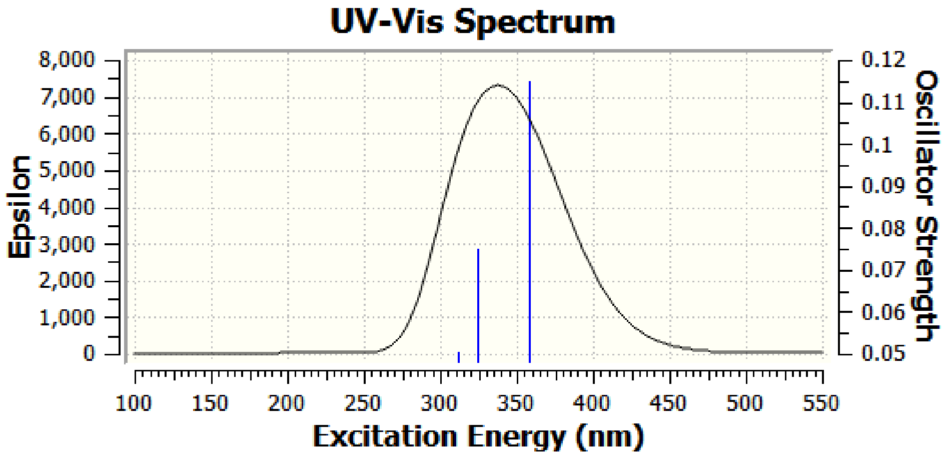

DMSO

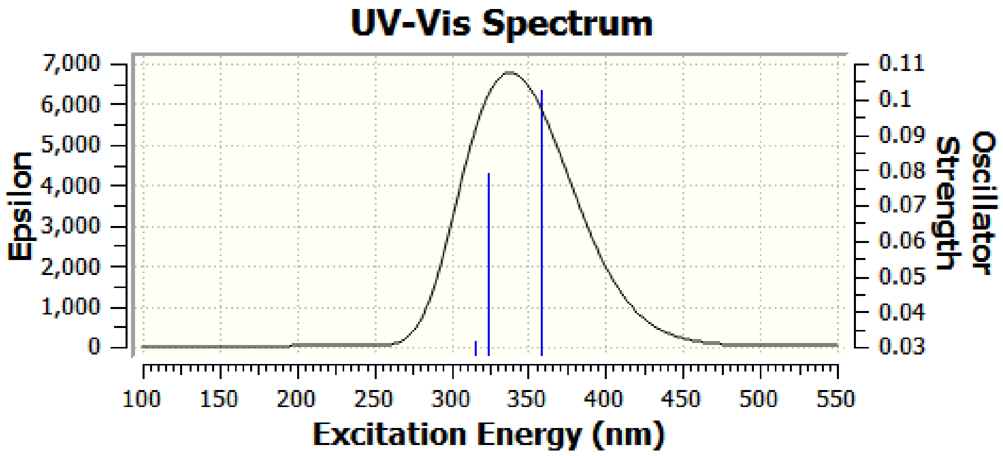

MeOH

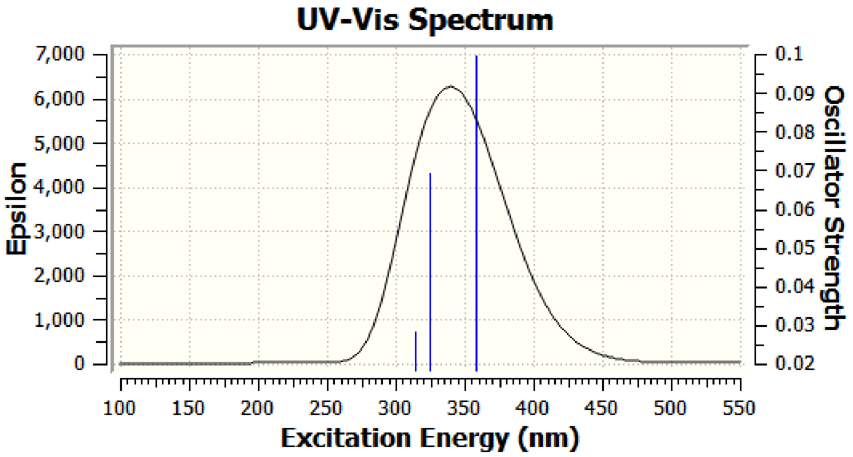

Figure S65.

CH_2Cl_2

HOMO **LUMO**

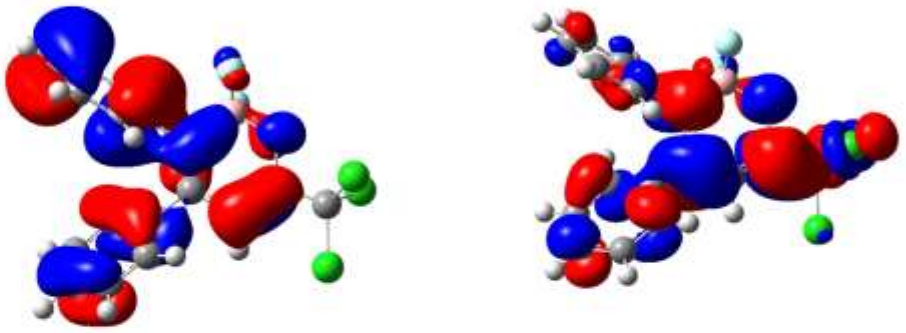

Figure S66.

DMSO

HOMO **LUMO**

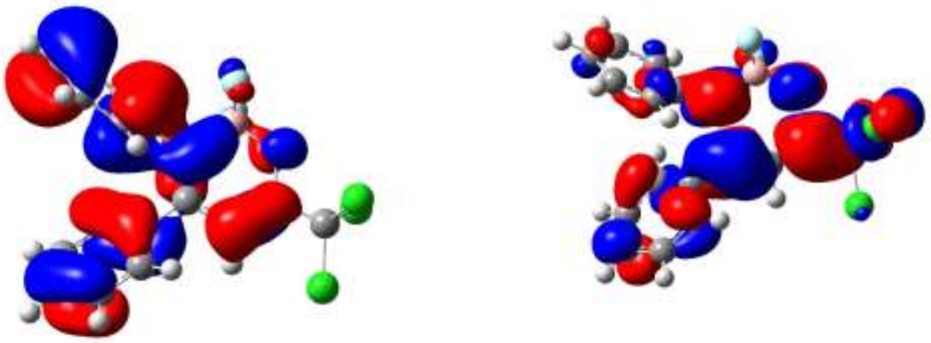

Figure S67.

MeOH

HOMO **LUMO**

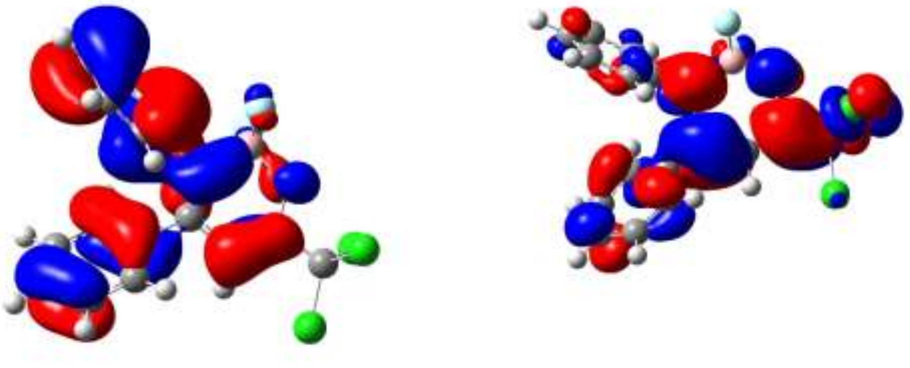

Table S6. Excitation energy (E), wavelength of maximum absorbance (λ_{max}), and oscillator strengths (*f*) for HOMO-LUMO orbitals in CH_2Cl_2, DMSO and MeOH for compound **6d**. Calculated at the TD-DFT (SCRF(PCM))-B3LYP/cc-pVTZ level.

Solvente	Eletronic Transitions	Energy (eV)	λmax (nm)	Main Orbital Transition	*F*
CH_2Cl_2	$S_0 \rightarrow S_1$	3.2544	380.98	102 ->103 0.69992	0.1387
	$S_0 \rightarrow S_2$	3.7778	328.19	100 ->103 -0.13860<br>101 ->103 0.69026	0.0550
	$S_0 \rightarrow S_3$	3.9832	311.27	99 ->103 0.59005<br>100 ->103 -0.36075	0.0647
DMSO	$S_0 \rightarrow S_1$	3.2558	380.81	102 ->103 0.70024	0.1263
	$S_0 \rightarrow S_2$	3.7751	328.43	100 ->103 -0. 14824 101 ->103 0. 68828	0.0605
	$S_0 \rightarrow S_3$	3.9394	314.73	99 ->103 0.62403<br>100 ->103 0.30944	0.0261
MeOH	$S_0 \rightarrow S_1$	3.2581	380.54	102 ->103 0.69998	0.1234
	$S_0 \rightarrow S_2$	3.7771	328.25	100 ->103 -0.15716<br>101 ->103 0.68630	0.0540
	$S_0 \rightarrow S_3$	3.9451	314.28	99 ->103 0.63143<br>100 ->103 -0.29398	0.0238

Figure S68. Calculated UV-Vis spectra for compound **6d** in DCM, DMSO and MeOH.

CH_2Cl_2

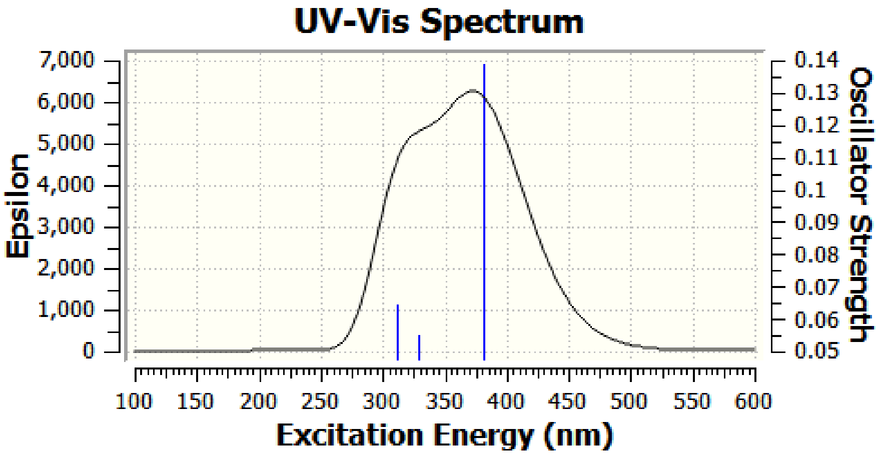

DMSO

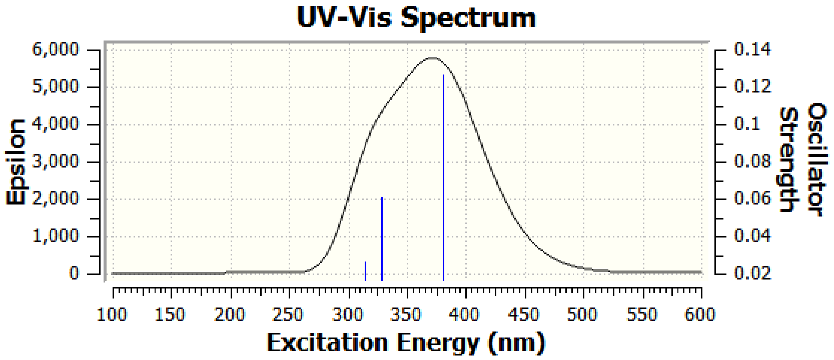

MeOH

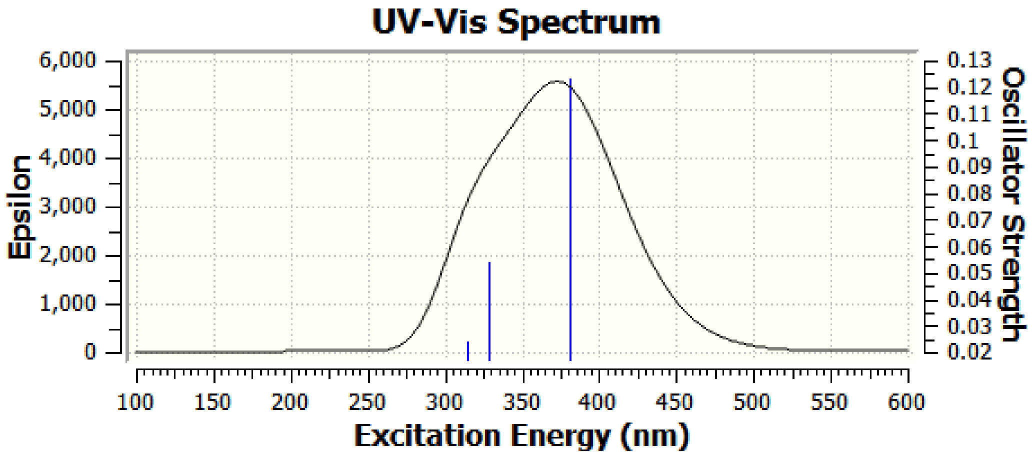

Figure S69

CH_2Cl_2

HOMO LUMO

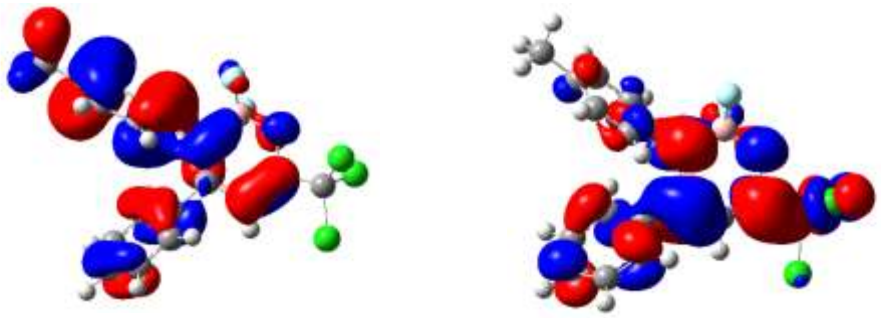

Figure S70.

DMSO

HOMO **LUMO**

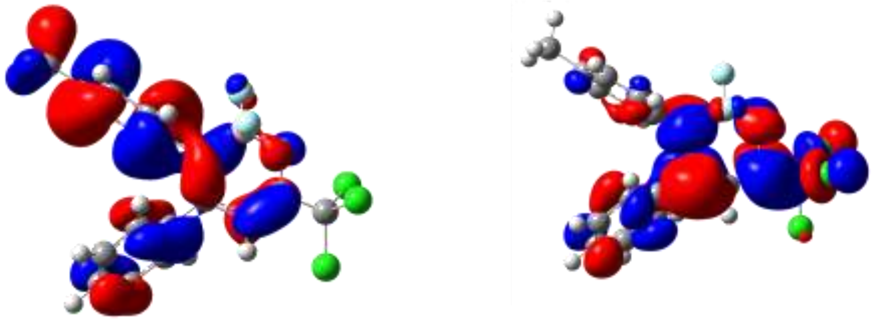

Figure S71.

MeOH

HOMO **LUMO**

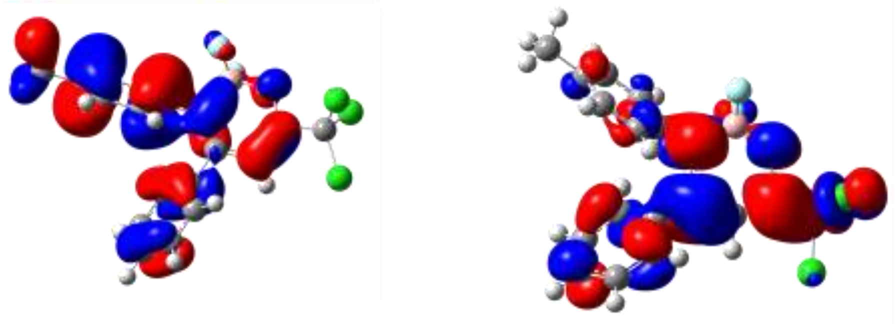

Table S7. Excitation energy (E), wavelength of maximum absorbance (λ_{max}), and oscillator strengths (f) for HOMO-LUMO orbitals in CH_2Cl_2, DMSO and MeOH for compound **6e**. Calculated at the TD-DFT (SCRF(PCM))-B3LYP/cc-pVTZ level.

Solvente	Eletronic Transitions	Energy (eV)	λmax (nm)	Main Orbital Transition	*F*
CH_2Cl_2	$S_0 \rightarrow S_1$	2.8912	428.83	106 ->107 0.70356	0.1437
	$S_0 \rightarrow S_2$	3.8371	323.12	104 ->107 −0. 18783<br>105 ->107 0. 67812	0.1100
	$S_0 \rightarrow S_3$	3.9917	310.61	103 ->107 0.66599<br>104 ->107 -0.20660	0.0170
DMSO	$S_0 \rightarrow S_1$	2.8904	428.95	106 ->107 0.70360	0.1327
	$S_0 \rightarrow S_2$	3.8281	323.88	104 ->107 −0. 17206<br>105 ->107 0. 68097	0.1277
	$S_0 \rightarrow S_3$	3.9450	314.28	103 ->107 0.61897<br>104 ->107 -0.32463	0.0141
MeOH	$S_0 \rightarrow S_1$	2.8936v	428.48	106 ->107 0.70350	0.1296
	$S_0 \rightarrow S_2$	3.8319	323.56	104 ->107 -0.18571<br>105 ->107 0.67745	0.1139
	$S_0 \rightarrow S_3$	3.9510	313.80	103 ->107 0.63214<br>104 ->107 -0.29783	0.0134

Figure S72. Calculated UV-Vis spectra for compound **6e** in DCM, DMSO and MeOH.

CH_2Cl_2

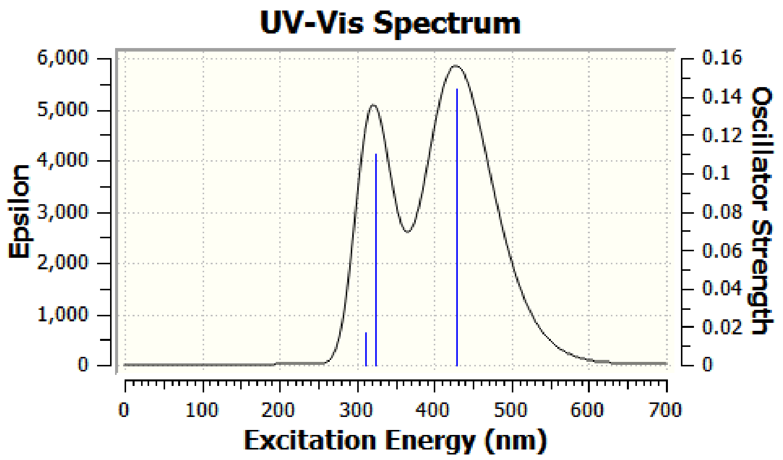

DMSO

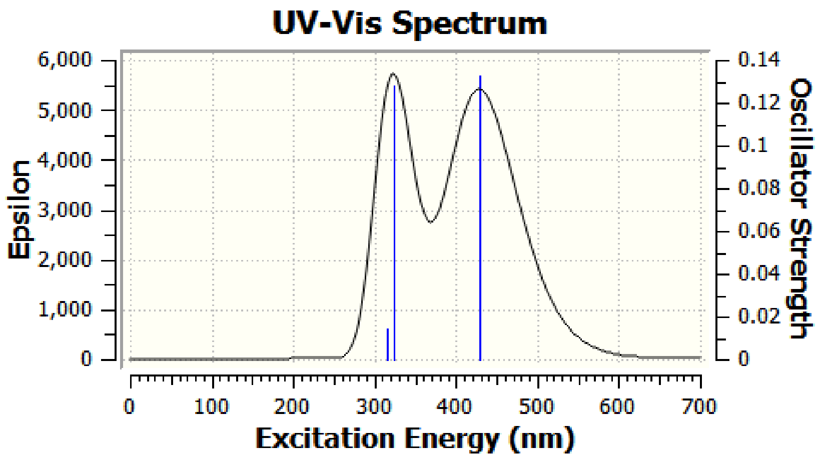

MeOH

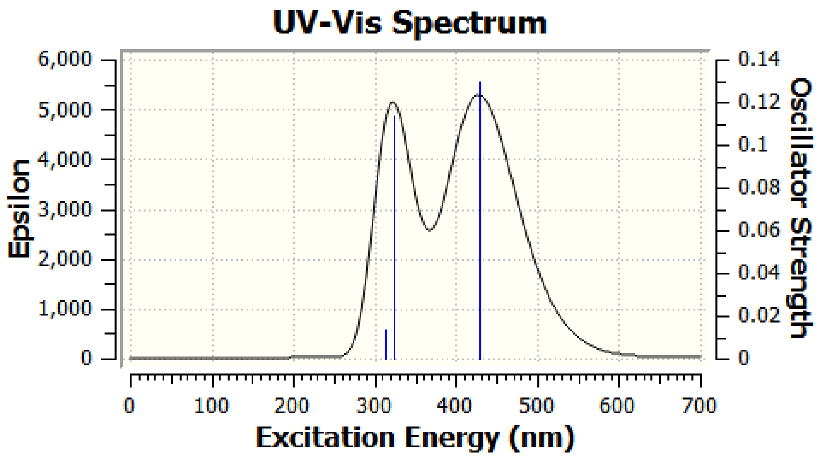

Figure S73.

CH_2Cl_2

HOMO **LUMO**

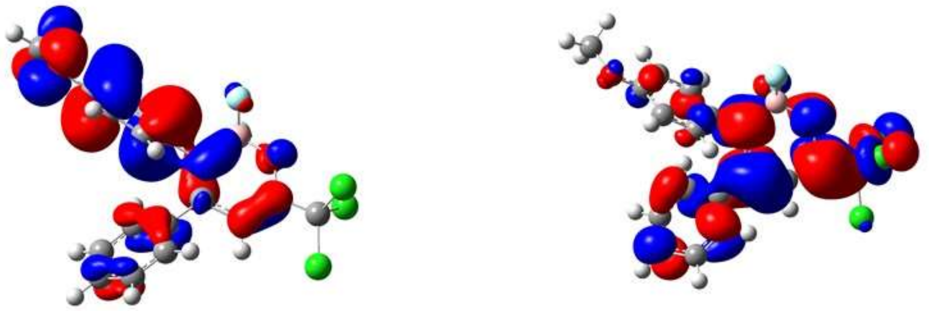

Figure S74.

DMSO

HOMO **LUMO**

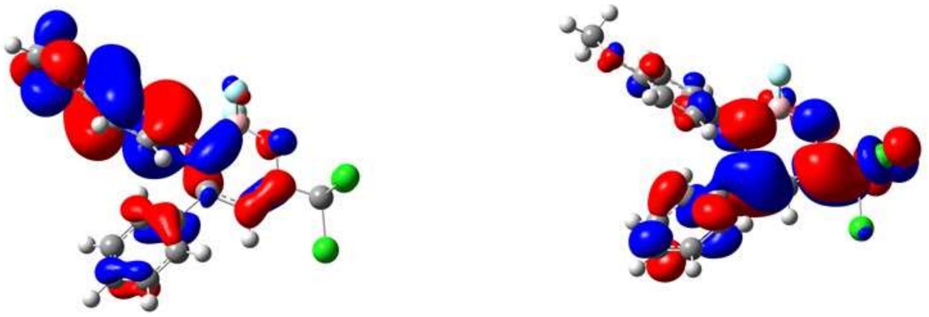

Figure S75.

MeOH

HOMO **LUMO**

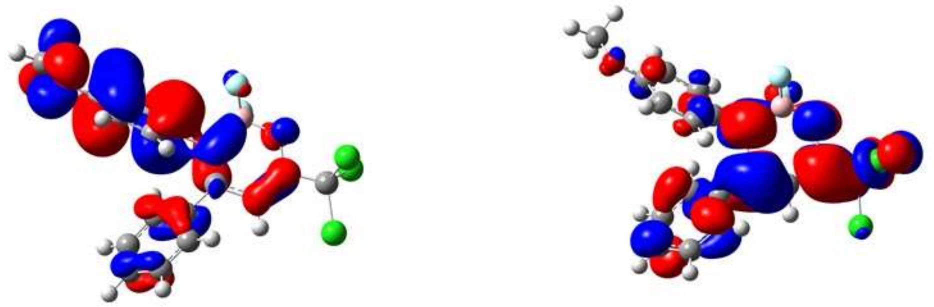

Table S8. Excitation energy (E), wavelength of maximum absorbance (λ_{max}), and oscillator strengths (*f*) for HOMO-LUMO orbitals in CH_2Cl_2, DMSO and MeOH for compound **6f**. Calculated at the TD-DFT (SCRF(PCM))-B3LYP/cc-pVTZ level.

Solvente	Eletronic Transitions	Energy (eV)	λmax (nm)	Main Orbital Transition	*F*
CH_2Cl_2	$S_0 \rightarrow S_1$	3.5466	349.59	108 ->110 0.17814<br>109 ->110 0.67653	0.2502
	$S_0 \rightarrow S_2$	3.7732	328.60	104 ->110 0.14865<br>104 ->111 0.15791<br>108 ->110 0.63710<br>109 ->110 –<br>0. 16808	0.4102
	$S_0 \rightarrow S_3$	3.7919	326.97	107 ->110 0.69116	0.0238
DMSO	$S_0 \rightarrow S_1$	3.5318	351.05	108 ->110 0.12826<br>109 ->110 0.68633	0.2227
	$S_0 \rightarrow S_2$	3.7419	331.34	107 ->110 0.69158<br>107 ->111 -0.10871	0.0227
	$S_0 \rightarrow S_3$	3.7631	329.47	104 ->110 0.13451<br>104 ->111 0.13661<br>108 ->110 0.65569<br>109 ->110 -0.12666	0.4408
MeOH	$S_0 \rightarrow S_1$	3.5390	350.34	108 ->110 0.13849<br>109 ->110 0.68407	0.2162
	$S_0 \rightarrow S_2$	3.7485	330.76	107 ->110 0.69267<br>107 ->111 -0.10681	0.0217
	$S_0 \rightarrow S_3$	3.7718	328.72	104 ->110 0.14919<br>104 ->111 0.15167<br>108 ->110 0.64870<br>109 ->110 -0.13342	0.4146

Figure S76. Calculated UV-Vis spectra for compound **6f** in DCM, DMSO and MeOH.

CH_2Cl_2

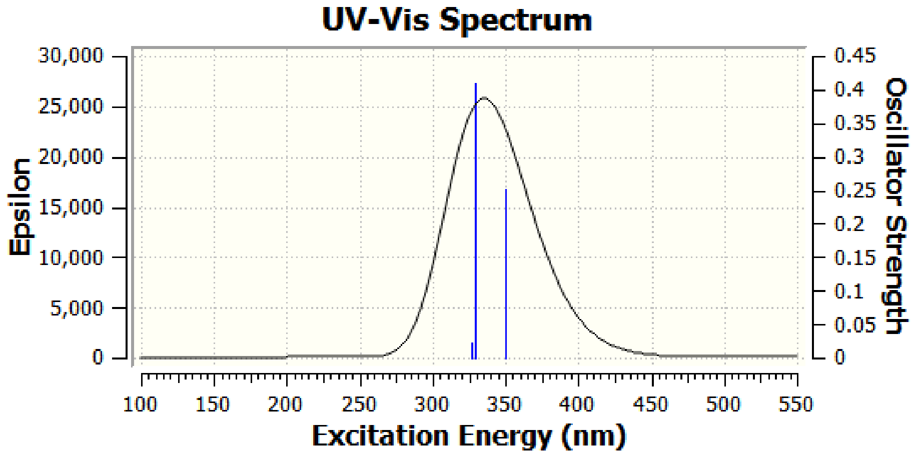

DMSO

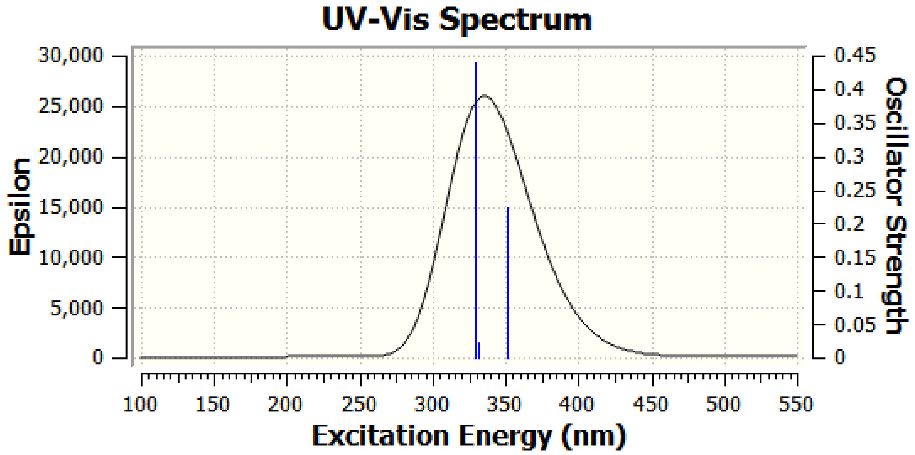

MeOH

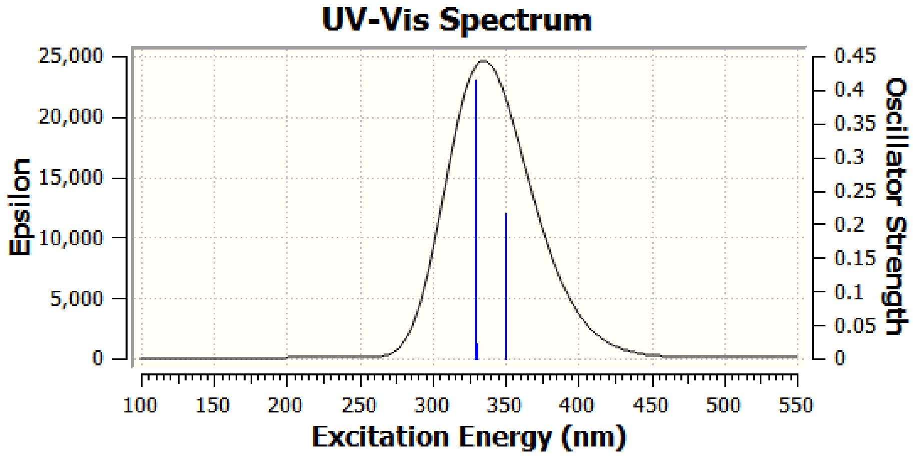

Figure S77.

CH_2Cl_2

HOMO LUMO

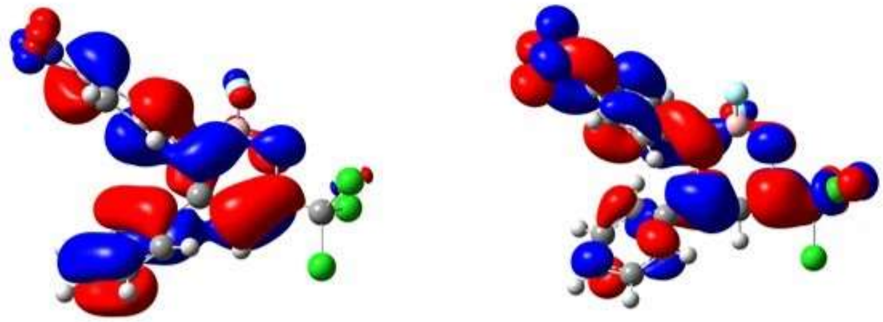

Figure S78.

DMSO

HOMO LUMO

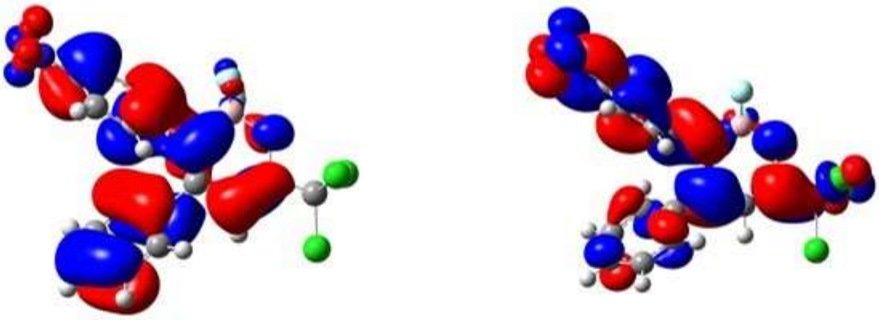

Figure S79.

MeOH

HOMO LUMO

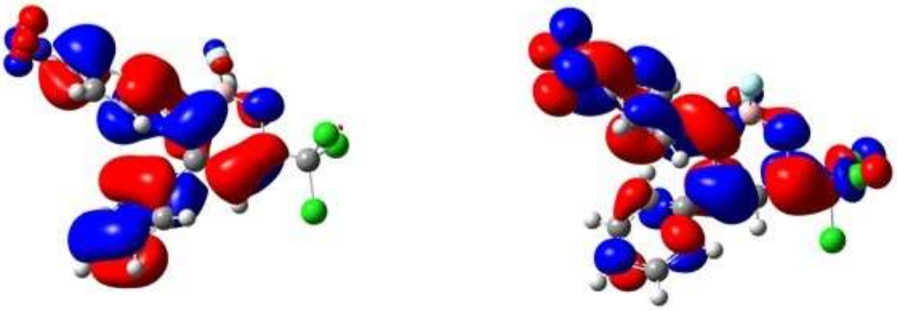

Table S9. Excitation energy (E), wavelength of maximum absorbance (λ_{max}), and oscillator strengths (*f*) for HOMO-LUMO orbitals in CH_2Cl_2, DMSO and MeOH for compound **6g**. Calculated at the TD-DFT (SCRF(PCM))-B3LYP/cc-pVTZ level.

Solvente	Eletronic Transitions	Energy (eV)	λmax (nm)	Main Orbital Transition	*F*
CH_2Cl_2	$S_0 \rightarrow S_1$	3.3706	367.84	102 ->103 0.69663	0.1218
	$S_0 \rightarrow S_2$	3.9233	316.02	99 ->103 0.14231<br>100 ->103 0.16043<br>101 ->103 0.66157	0.3557
	$S_0 \rightarrow S_3$	3.9650	312.69	100 ->103 0.67958<br>101 ->103 -0.13450	0.0223
DMSO	$S_0 \rightarrow S_1$	3.3773	367.11	102 ->103 0.69708	0.1092
	$S_0 \rightarrow S_2$	3.9085	317.21	100 ->103 0.45508<br>101 ->103 0.52444	0.2532
	$S_0 \rightarrow S_3$	3.9246	315.91	99 ->103 -0.13004<br>100 ->103 0.53220<br>101 ->103 -0.42768	0.1343
MeOH	$S_0 \rightarrow S_1$	3.3794	366.89	102 ->103 0.69670	0.1066
	$S_0 \rightarrow S_2$	3.9166	316.56	100 ->103 0.49542<br>101 ->103 0.48747	0.2142
	$S_0 \rightarrow S_3$	3.9309	315.41	99 ->103 -0.14734<br>100 ->103 0.49462<br>101 ->103 -0.46412	0.1517

Figure S80. Calculated UV-Vis spectra for compound **6g** in DCM, DMSO and MeOH.

CH_2Cl_2

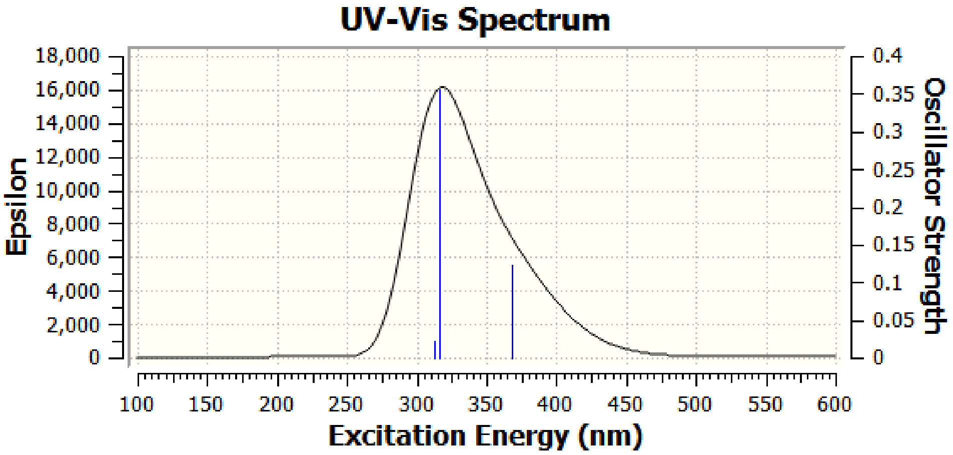

DMSO

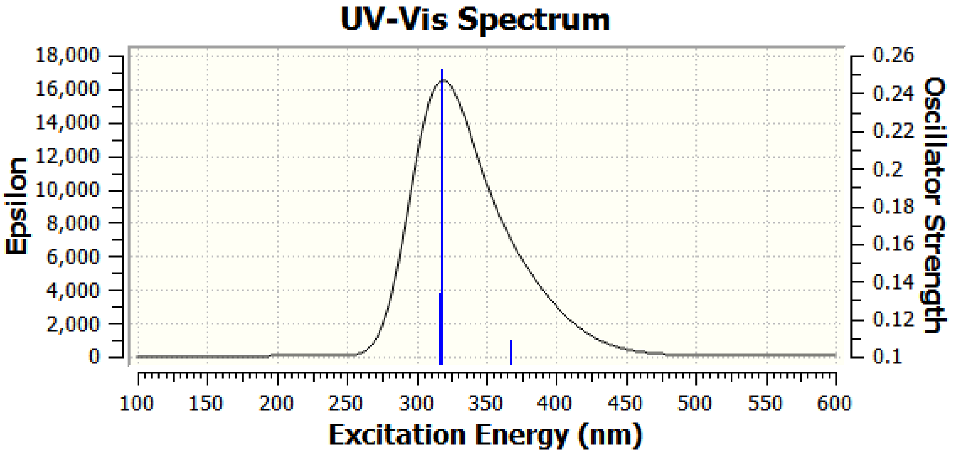

MeOH

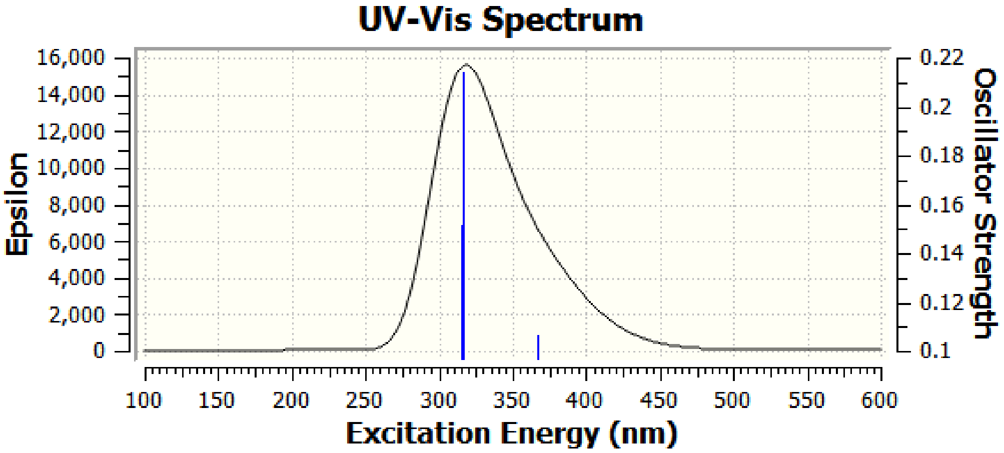

Figure S81.

CH_2Cl_2

HOMO LUMO

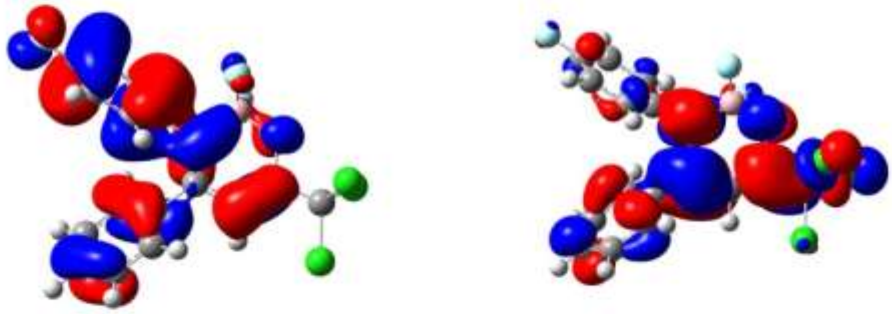

Figure S82.

DMSO

HOMO LUMO

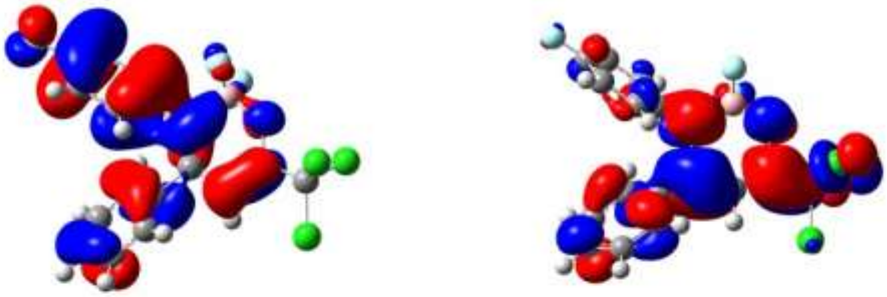

Figure S83.

MeOH

HOMO LUMO

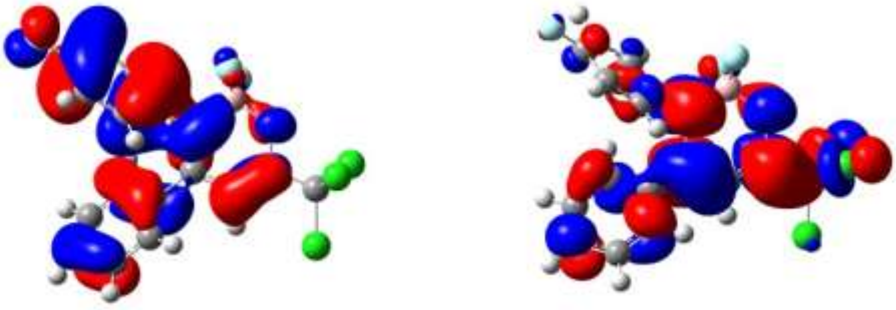

Table S10. Excitation energy (E), wavelength of maximum absorbance (λ_{max}), and oscillator strengths (*f*) for HOMO-LUMO orbitals in CH_2Cl_2, DMSO and MeOH for compound **6h.** Calculated at the TD-DFT (SCRF(PCM))-B3LYP/cc-pVTZ level.

Solvente	Eletronic Transitions	Energy (eV)	λmax (nm)	Main Orbital Transition	*F*
CH_2Cl_2	$S_0 \rightarrow S_1$	3.3140	374.12	115 ->116 0.69941	0.1570
	$S_0 \rightarrow S_2$	3.8941	318.39	112 ->116 -0.17641<br>114 ->116 0.66920	0.3588
	$S_0 \rightarrow S_3$	3.9371	314.91	113 ->116 0.69238	0.0165
DMSO	$S_0 \rightarrow S_1$	3.3232	373.08	115 ->116 0.69933	0.1443
	$S_0 \rightarrow S_2$	3.8804	319.51	112 ->116 -0.14436<br>113 ->116 0.24642<br>114 ->116 0.63880	0.3429
	$S_0 \rightarrow S_3$	3.8931	318.47	113 ->116 0.65601<br>114 ->116 -0.22534	0.0414
MeOH	$S_0 \rightarrow S_1$	3.3252	372.86	115 ->116 0.69914	0.1406
	$S_0 \rightarrow S_2$	3.8892	318.79	112 ->116 -0.15527<br>113 ->116 0.26336<br>114 ->116 0.62962	0.3194
	$S_0 \rightarrow S_3$	3.8987	318.01	112 ->116 0.10542<br>113 ->116 0.64922<br>114 ->116 -0.23948	0.0435

Figure S84. Calculated UV-Vis spectra for compound **6h** in DCM, DMSO and MeOH.

CH_2Cl_2

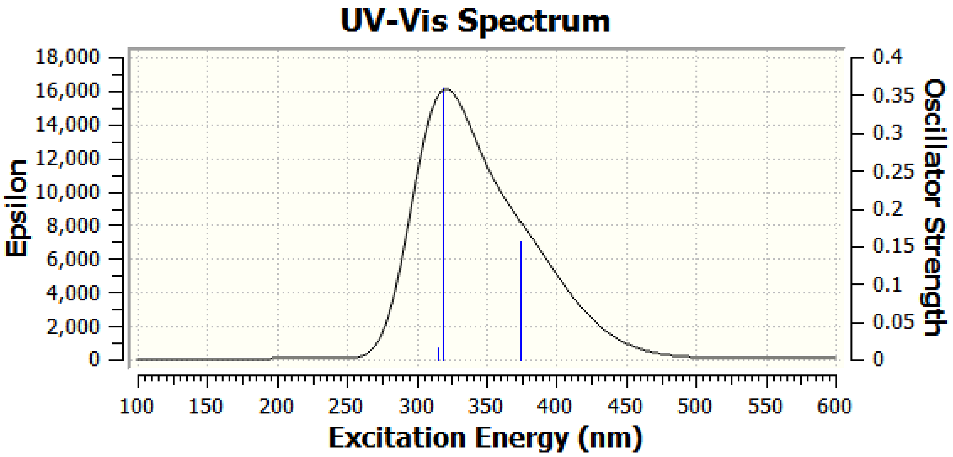

DMSO

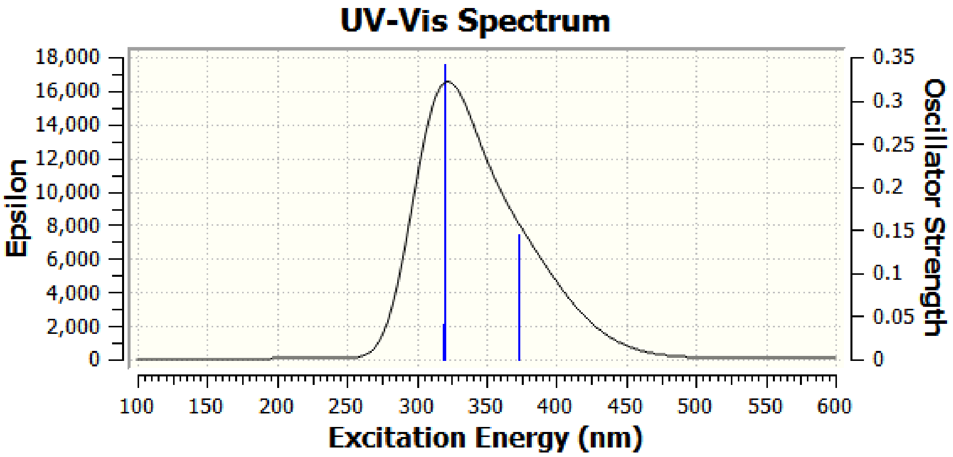

MeOH

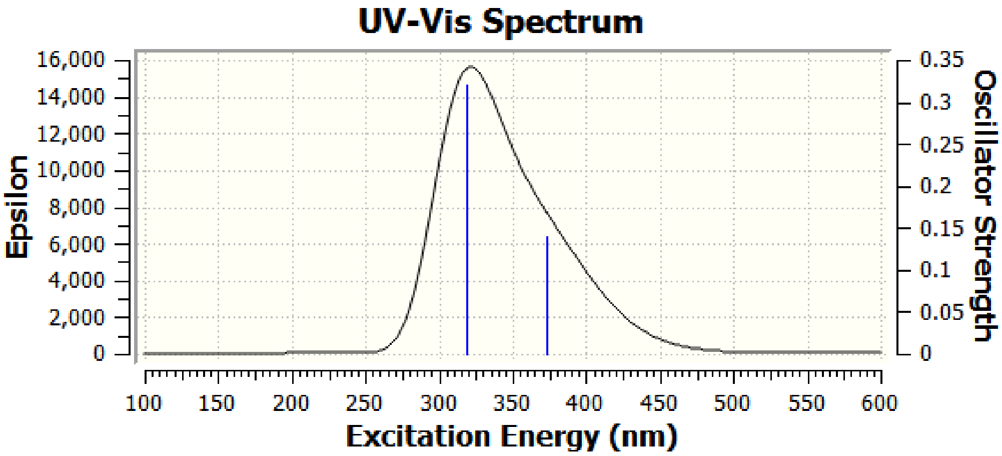

Figure S85.

CH_2Cl_2

HOMO **LUMO**

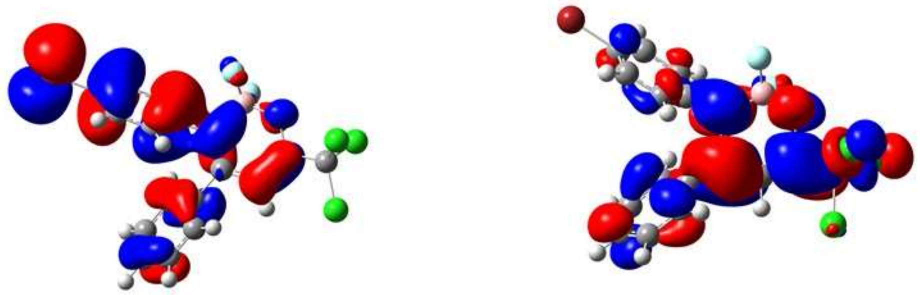

Figure S86.

DMSO

HOMO **LUMO**

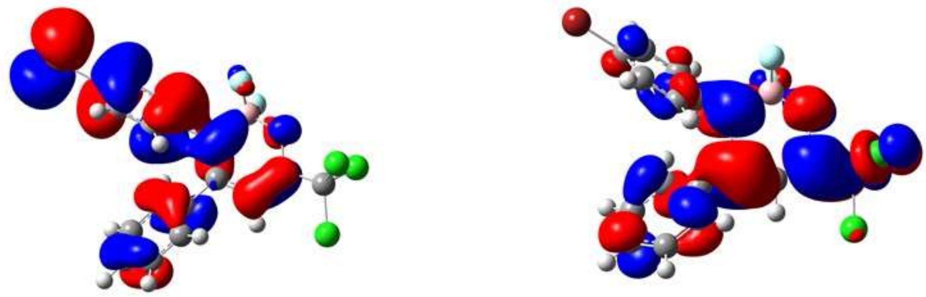

Figure S87.

MeOH

HOMO **LUMO**

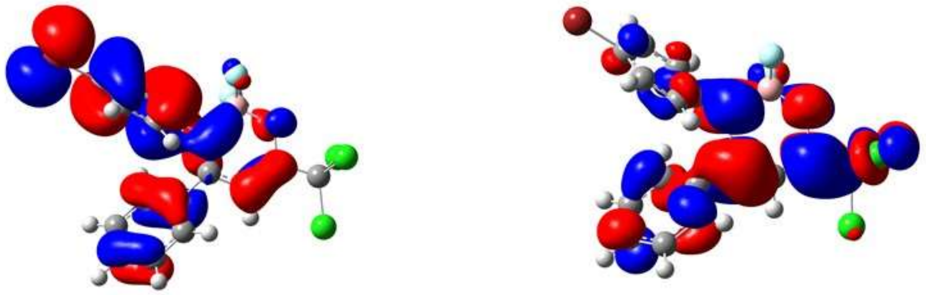

Table S11. Excitation energy (E), wavelength of maximum absorbance (λ_{max}), and oscillator strengths (*f*) for HOMO-LUMO orbitals in CH_2Cl_2, DMSO and MeOH for compound **6i.** Calculated at the TD-DFT (SCRF(PCM))-B3LYP/cc-pVTZ level.

Solvente	Eletronic Transitions	Energy (eV)	λmax (nm)	Main Orbital Transition	*F*
CH_2Cl_2	$S_0 \rightarrow S_1$	2.8648	432.79	111 -> 112 0.70382	0.0626
	$S_0 \rightarrow S_2$	3.4160	362.95	110 -> 112 0.69952	0.1202
	$S_0 \rightarrow S_3$	3.9523	313.70	107 -> 112 0.16602<br>108 -> 112 0.20495<br>109 -> 112 0.64650	0.4126
DMSO	$S_0 \rightarrow S_1$	2.8674	432.39	111 -> 112 0.70380	0.0577
	$S_0 \rightarrow S_2$	3.4161	362.94	110 -> 112 0.69961	0.1130
	$S_0 \rightarrow S_3$	3.9143	316.74	108 -> 112 0.67844<br>109 -> 112 0.17100	0.0258
MeOH	$S_0 \rightarrow S_1$	2.8681	432.28	111 -> 112 0.70379	0.0556
	$S_0 \rightarrow S_2$	3.4183	362.71	110 -> 112 0.69954	0.1097
	$S_0 \rightarrow S_3$	3.9204	316.26	108 -> 112 0.67860<br>109 -> 112 0.16929	0.0246

Figure S88. Calculated UV-Vis spectra for compound **6i** in DCM, DMSO and MeOH.

CH_2Cl_2

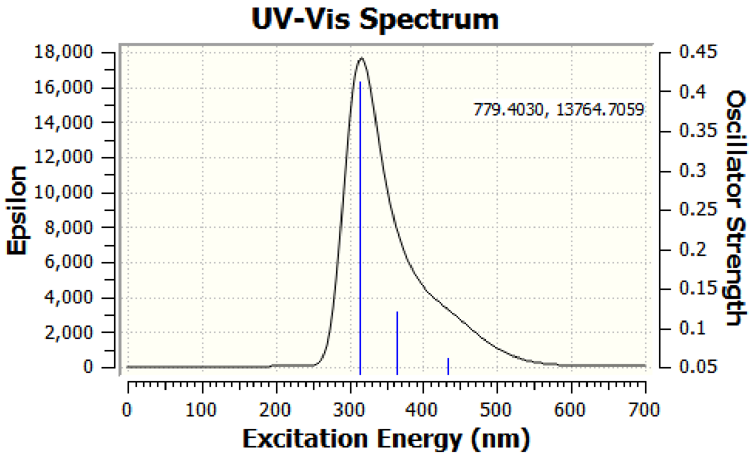

DMSO

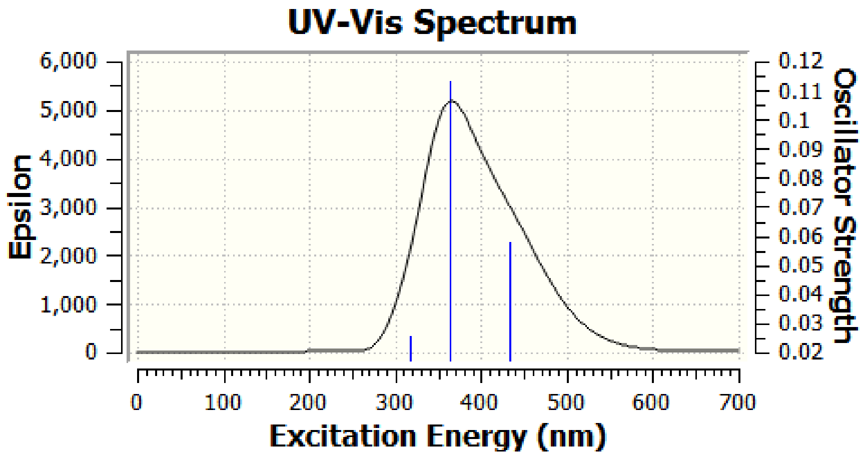

MeOH

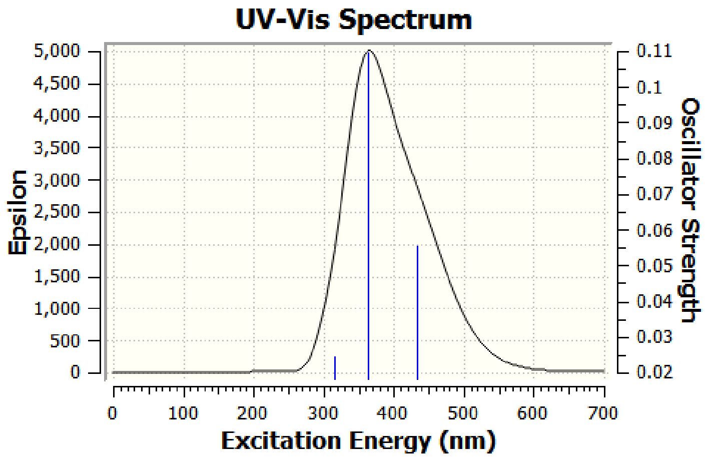

Figure S89

CH_2Cl_2

HOMO **LUMO**

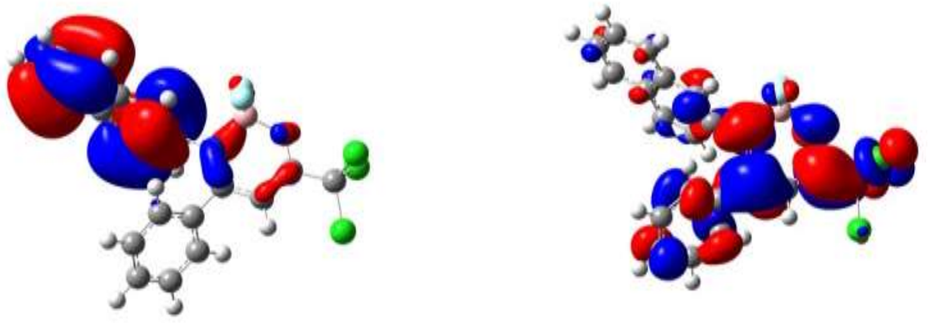

Figure S90.

DMSO

HOMO **LUMO**

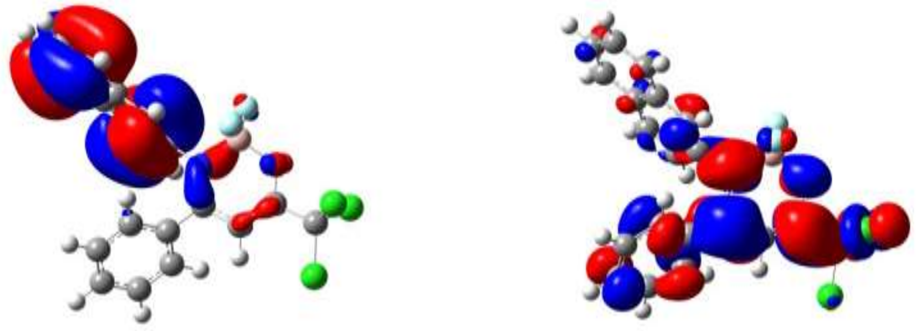

Figure S91.

MeOH

HOMO **LUMO**

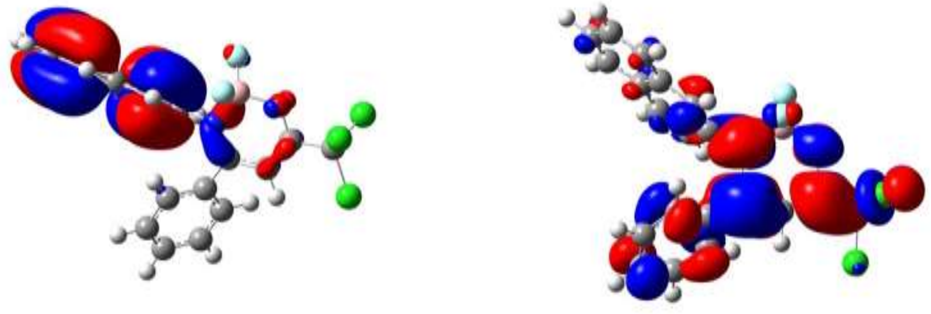

Table S12. Excitation energy (E), wavelength of maximum absorbance (λ_{max}), and oscillator strengths (*f*) for HOMO-LUMO orbitals in CH_2Cl_2, DMSO and MeOH for compound **7e.** Calculated at the TD-DFT (SCRF(PCM))-B3LYP/cc-pVTZ level.

Solvente	Eletronic Transitions	Energy (eV)	λmax (nm)	Main Orbital Transition	*F*
CH_2Cl_2	$S_0 \rightarrow S_1$	2.8972	427.95	110 -> 111 0.70357	0.1386
	$S_0 \rightarrow S_2$	3.7197	333.32	109 -> 111 0.69794	0.2851
	$S_0 \rightarrow S_3$	3.9445	314.32	107 -> 111 0.53902<br>108 -> 111 -0.44496	0.0171
DMSO	$S_0 \rightarrow S_1$	2.8937	428.46	110 -> 111 0.70364	0.1270
	$S_0 \rightarrow S_2$	3.6931	335.72	109 -> 111 0.69935	0.2910
	$S_0 \rightarrow S_3$	3.9084	317.23	108 -> 111 0.69697	0.0083
MeOH	$S_0 \rightarrow S_1$	2.8971	427.96	110 -> 111 0.70355	0.1242
	$S_0 \rightarrow S_2$	3.7017	334.94	109 -> 111 0.69816	0.2760
	$S_0 \rightarrow S_3$	3.9132	316.84	108 -> 111 0.69811	0.0078

Figure S92. Calculated UV-Vis spectra for compound **7e** in DCM, DMSO and MeOH.

CH_2Cl_2

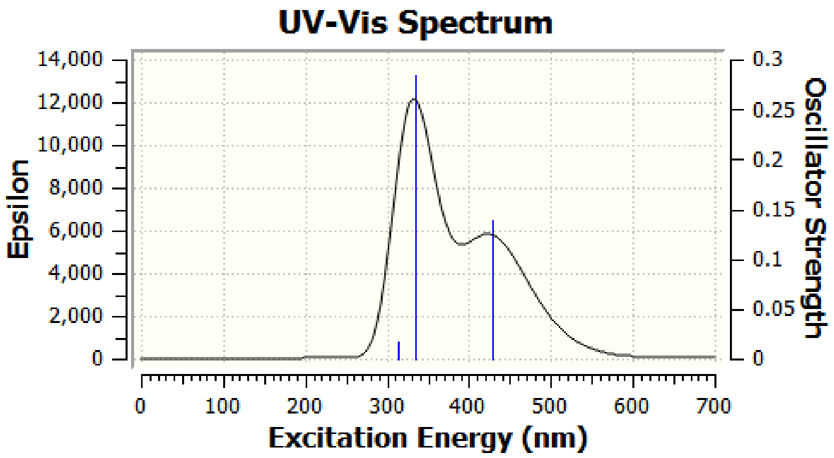

DMSO

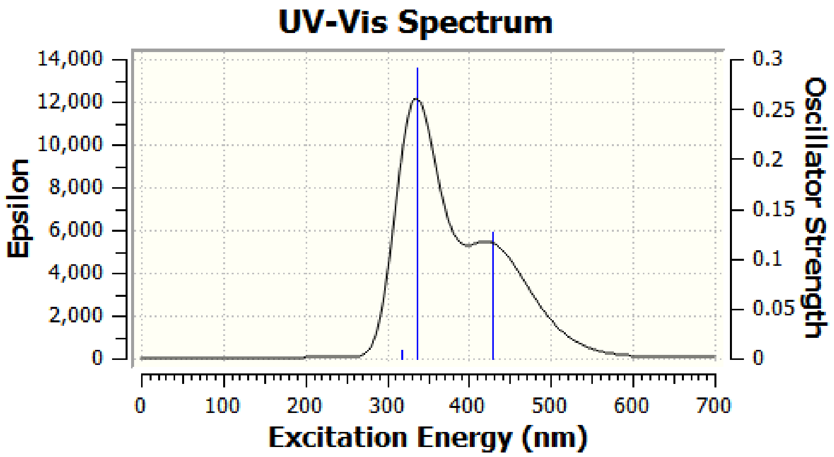

MeOH

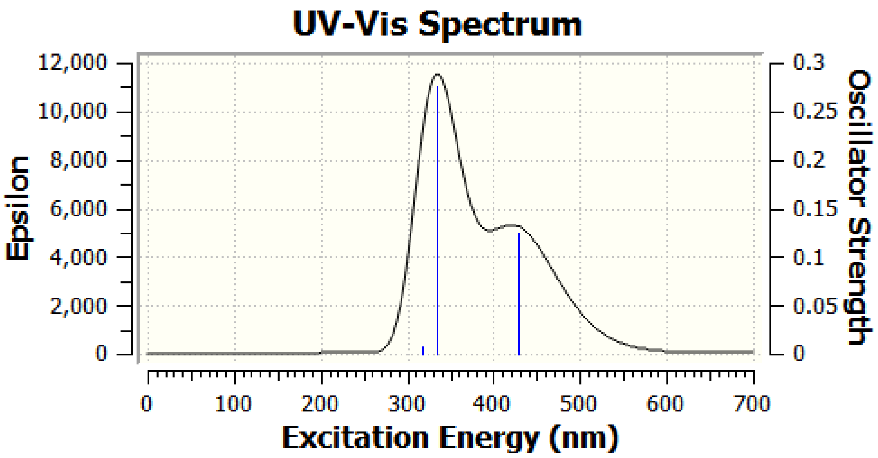

Figure S93.

CH_2Cl_2

HOMO LUMO

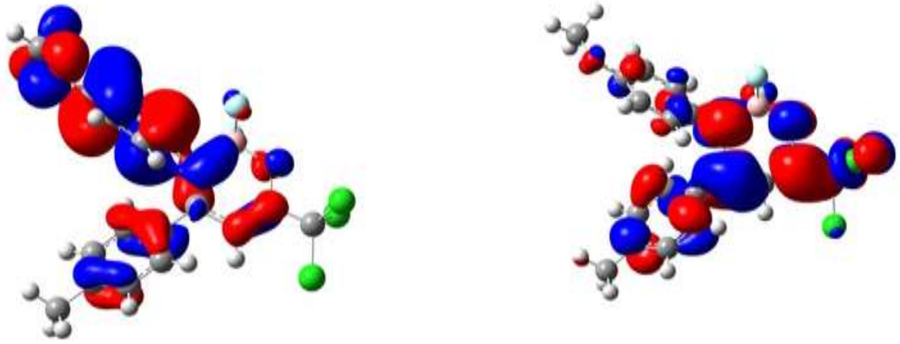

Figure S94

DMSO

HOMO LUMO

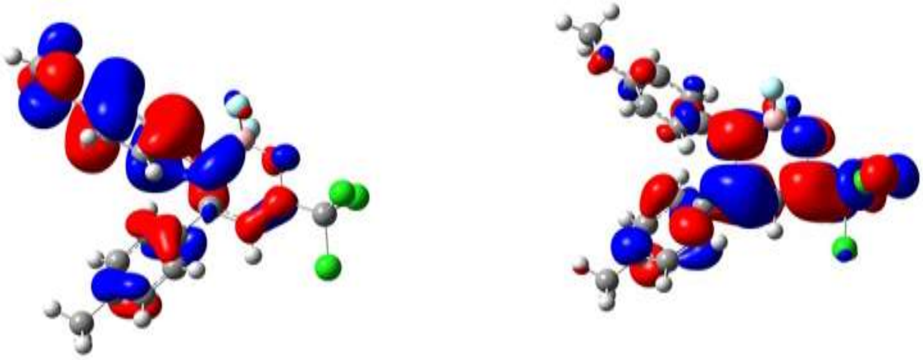

Figure S95.

MeOH

HOMO LUMO

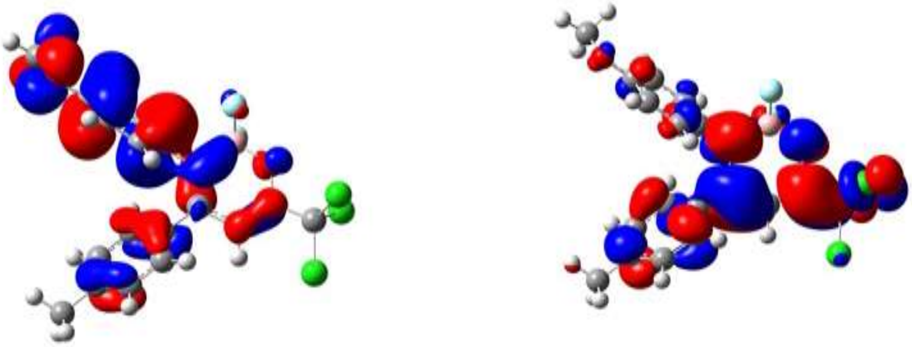

9. Antimicrobial assays

The *in vitro* antimicrobial activity of the β-enaminoketone and boron complex compounds was assessed against a panel of microorganisms including yeast-like fungi such as *Candida albicans* ATCC 44373, *Candida glabrata* ATCC 10231, *Candida tropicalis* ATCC 750, *Cryptococcus gatti* ATCC 28952 and *Saccharomyces cerevisiae* ATCC 2601. Filamentous fungi such as *Aspergillus flavus*, *Aspergillus fumigatus*, *Aspergillus niger*, *Aspergillus terreus*, as well as a clinical isolate of *Prototheca zopfii*, an alga of medicinal and veterinary importance, were also included. Among the bacteria, we included *Staphylococcus aureus* ATCC 25923, *Bacillus subtilis* ATCC 19659, *Klebsiella pneumoniae* clinical isolate, *Salmonella typhimurium* ATCC 14028, and *Pseudomonas aeruginosa* ATCC 9027.

The minimal inhibitory concentration (MIC) and the minimal fungicidal/bactericidal/algacidal concentrations were determined by broth microdilution methods according to CLSI standards.[1,2,3] Compounds were dissolved in DMSO and the work solutions were diluted in a culture medium. By further progressive dilutions with the test medium, the required concentrations (80, 40, 20, 10, 5, 2.5, 1.25, 0.62, 0.31 e 0.15 μg/mL) were obtained. The antimicrobial activities were evaluated based on the minimal inhibitory concentration (MIC) according to the CLSI M27-A3 procedures[1] for yeast-like fungi and the *P. zopfii*. The filamentous fungi were tested based on the CLSI M38-A2[2] procedures and for the bacteria, the procedures described in CLSI M7-A10,[3] were employed. Bacteria were initially inoculated into Mueller-Hinton agar and, after overnight growth, approximately five

[1] CLINICAL AND LABORATORY STANDARDS INSTITUTE (CLSI). **Document M07-A10. Reference Method for Dilution Antimicrobial Susceptibility Test of bacteria that grow aerobically; Approved Standard.** 10th ed. Clinical and Laboratory Standards Institute: Wayne, Pennsylvania, USA, 2015.

[2] CLINICAL AND LABORATORY STANDARDS INSTITUTE (CLSI). **Document M27-S4. Reference Method for Broth Dilution Antifungal Susceptibility Testing of yeasts; Approved Standard.** 4th ed. informational gsupplemetgl. Clinical and Laboratory Standards Institute: Wayne, Pennsylvania, USA, 2012.

[3] CLINICAL AND LABORATORY STANDARDS INSTITUTE (CLSI). **Document M38-A2. Reference Method for Broth Dilution Antifungal Susceptibility Testing of Filamentous Fungi; Approved Standard.** 2nd ed. Clinical and Laboratory Standards Institute: Wayne, Pennsylvania, USA, 2008.

colonies were directly suspended in saline solution until the turbidity matched the turbidity of the McFarland standard (approximately 10^8 cfu/mL). The suspensions were diluted to 1:100 in saline followed by a new dilution to 1:20 in Mueller-Hinton broth, resulting in a final inoculum concentration of $5x10^4$ cfu/mL per well. Yeasts and *P. zopfii* were inoculated on Sabouraud dextrose agar and the procedures of inoculum standardization were similar; the test medium was RPMI 1640 buffered with MOPS (3-(*N*-morpholino) propanesulfonic acid), pH 7.0. The filamentous fungi were initially inoculated on potato dextrose agar; after the time required for each species to induce conidium and sporangiospore formation, the inoculum standardization followed that described in the CLSI, M38-A2[2] protocol. Briefly, each well of the microdilution plate was filled with 100μL of compound diluted in 100μL of the inoculum. The plates were incubated at 35ºC/24h for the bacteria strains and the yeast required 48h of incubation (except the Cryptococcus strains that were incubated for 72 h). For all tests growth and negative (inoculum-free) controls were performed. Growth or a lack of growth in the wells containing the antimicrobial agent was determined by comparison with the growth control, indicated by turbidity. The lowest concentration that completely inhibited visible growth of the organism was recorded as the MIC. All tests were carried out in duplicate did accepted if coincident. When the test not coincident they were repeated in duplicate, again.

The minimal fungicidal, bactericidal and algacidal concentrations were determined by subculture of 20 μL of the content of each well that remained clear. The media employed were Sabouraud dextrose agar for fungi and *P. zopfii* and Mueller-Hinton agar for bacteria. The plates were incubated at 35 ºC during the same time periods for MIC determination and the lowest concentration required to demonstrate complete growth absence was named "cidal".

The interpretation of the results was based on fluconazole (yeasts) and amphotericin B (filamentous fungi and *P. zopfii*) breakpoints and based on imipenem for bacterial strains; all according to the CLSI M27-S4[1], M38-A2[2] and M7-A10[3] techniques, respectively.

10. Cytotoxicity assays

The cytotoxic effects of β-enaminoketone boron complex compounds were measured by tetrazolium salt MTT assay[4] using mouse fibroblast 3T3 cell line as a model. 3T3 cells were grown in DMEM (Dulbecco's Modified Eagle's medium) supplemented with 10% FBS (Fetal Bovine Serum) and antibiotics in a 5% CO_2 atmosphere at 37 °C. 3T3 cells were detached by trypsinization and seeded in 96-wells plates at a density of 1×10^5 cells/mL. After incubation for 24 h under 5% CO2 at 37 °C to allow cells to attach to the plates, the spent medium was replaced with 200 µL of fresh medium containing the compounds in concentrations ranging from 1 to 100 µg/mL, besides a negative control were only the vehicle (DMSO) was added to the medium. After 24 h of incubation, the supernatant was removed, and 100 µl of fresh medium containing MTT reagent (initially diluted in PBS at 5 mg/mL and then diluted 1:10 in medium without FBS) was then added to the cells. The plates were further incubated for 2 h, after which the medium was removed and 200 µL of DMSO were then added to each well to dissolve the purple formazan product. After 10 min shaking at room temperature, the absorbance of the resulting solutions was measured at 590 nm using a microplate reader (Bio-Rad Laboratories, Hercules, CA). The effect of each treatment was calculated as a percentage of cell viability inhibition against the control.

[4] Mosmann T. *J. Immunol. Methods.* **1983**, *65*, 55.

Table S13. In vitro antimicrobial and cytotoxic activities of β-enaminoketone **4a-i** and **5e** boron complex **6a-i** and **7e** against yeast, filamentous fungi, alga and bacteria (MIC/MFC/MBC, μg/mL):

	MIC[a]/MFC[b]										MIC[a]/MBC[b]					
	Yeast					Filamentous fungi				Alga	Bacteria					
											Gram-positive		Gram-negative			
Compounds	*C. albincans*	*C. glabrata*	*C. tropicalis*	*C. gatti*	*S. cerevisiae*	*A. niger*	*A. fumigatus*	*A. flavus*	*A. terreus*	*P. zopfii*	*S. aureus*	*B. subtilis*	*K. pneumoniae*	*S. typhimurium*	*P. aeruginosa*	Cytotoxicity
**4a**	-	-	-	20/40	-	10/20	-	-	-	0,62/0,62	-	-	-	-	-	>100
**4b**	-	-	-	20/40	-	10/10	-	-	-	0,62/0,62	-	-	-	-	-	25
**4c**	-	-	-	-	-	2,5/2,5	-	40/40	-	1,25/5	-	-	-	-	-	25
**4d**	-	-	-	-	-	2,5/40	-	80/80	-	0,31/0,31	-	-	-	-	-	5
**4e**	-	-	-	-	-	2,5/-	-	80/80	-	0,31/0,31	-	-	-	-	-	50
**4f**	-	-	-	-	-	10/10	-	-	-	2,5/2,5	-	-	-	-	-	5
**4g**	-	-	-	-	-	2,5/2,5	80/-	20/20	80/-	-	-	-	-	-	-	10
**4h**	-	-	-	-	-	2,5/2,5	-	-	-	1,25/1,25	-	-	-	-	-	25
**4i**	-	-	-	-	-	-	-	-	-	10/10	-	-	-	-	-	25
**5e**	-	-	-	80/80	-	-	-	-	-	40/40	-	-	-	-	-	1
**6a**	-	-	-	40/80	-	40/40	-	-	-	5/5	-	-	-	-	-	5
**6b**	-	-	-	20/40	-	10/-	-	-	-	1,25/1,25	-	-	-	-	-	5
**6c**	80/80	-	-	20/40	-	10/10	80/80	40/40	80/-	0,62/0,62	20/40	20/80	5/10	20/80	-	5
**6d**	-	-	-	20/20	-	5/5	-	-	-	1,25/1,25	-	80/-	80/-	80/-	80/-	5
**6e**	-	-	-	20/20	-	5/40	-	40/40	80/80	0,31/0,31	-	-	-	-	-	1
**6f**	-	-	-	20/20	-	10/20	-	40/40	-	0,31/0,31	-	-	-	-	-	10
**6g**	-	-	-	20/20	-	10/20	-	40/40	80/-	1,25/1,25	20/80	20/80	10/40	40/80	-	10
**6h**	-	-	-	40/40	-	5/5	-	-	-	0,62/0,62	-	80/-	-	80/-	80/-	5
**6i**	40/40	80/80	40/40	20/80	-	5/40	-	20/20	80/-	1,25/1,25	20/40	20/80	5/20	20/80	-	5
**7e**	-	-	-	-	-	-	-	-	-	40/40	-	-	-	-	-	1
**FLZ**	4,0	8,0	4,0	2,0	1,0											
**AmB**						1,0	1,0	1,0	2,0	0,5						
**IMP**											0,06	2,0	<4,0	≤1,0	2,0	

CIM/CFM, Minimal inhibitory concentration/Minimal fungicidal concentration; CIM/CBM, Minimal inhibitory concentration/Minimal bactericidal concentration; *C. albincan, Candida albicans ATCC 44373; C. glabrata, Candida glabrata ATCC 10231; C. tropicalis, Candida tropicalis* ATCC 750; *C. gatti, Cryptococcus gatti* ATCC 28952*; C. cerevisiae, Saccharomyces cerevisiae* ATCC 2601*; A. niger , Aspergillus niger (clinical isolate); A. fumigatus, Aspergillus fumigatus* (clinical isolate); *A. flavus, Aspergillus flavus* (clinical isolate); *A. terreus, Aspergillus terreus* (clinical isolate); *Prototheca zopfii* (clinical isolate); *S. aureus*, *Staphylococcus aureus* ATCC 25923; *B. subtilis, Bacillus subtilis* ATCC19659; *K. pneumoniae*, *Klebsiella pneumoniae* (clinical isolate); S. *typhimurium, Salmonela typhimurium* ATCC 14028; *P. aeruginosa*, *Pseudomonas aeruginosa* ATCC 9027; FLZ, Fluconazole; AmB, Anfotericin B; IMP, Imipenem; -, No activity.
